# Supplementary material for: Advances in biomineralization-inspired materials for hard tissue repair
Source: Int J Oral Sci. 2021 Dec 7;13:42. doi: 10.1038/s41368-021-00147-z (PMC8651686; doi:10.1038/s41368-021-00147-z)
Supplement: Supplementary file 1 — Summary of Figures and captions [file 41368_2021_147_MOESM1_ESM.docx]

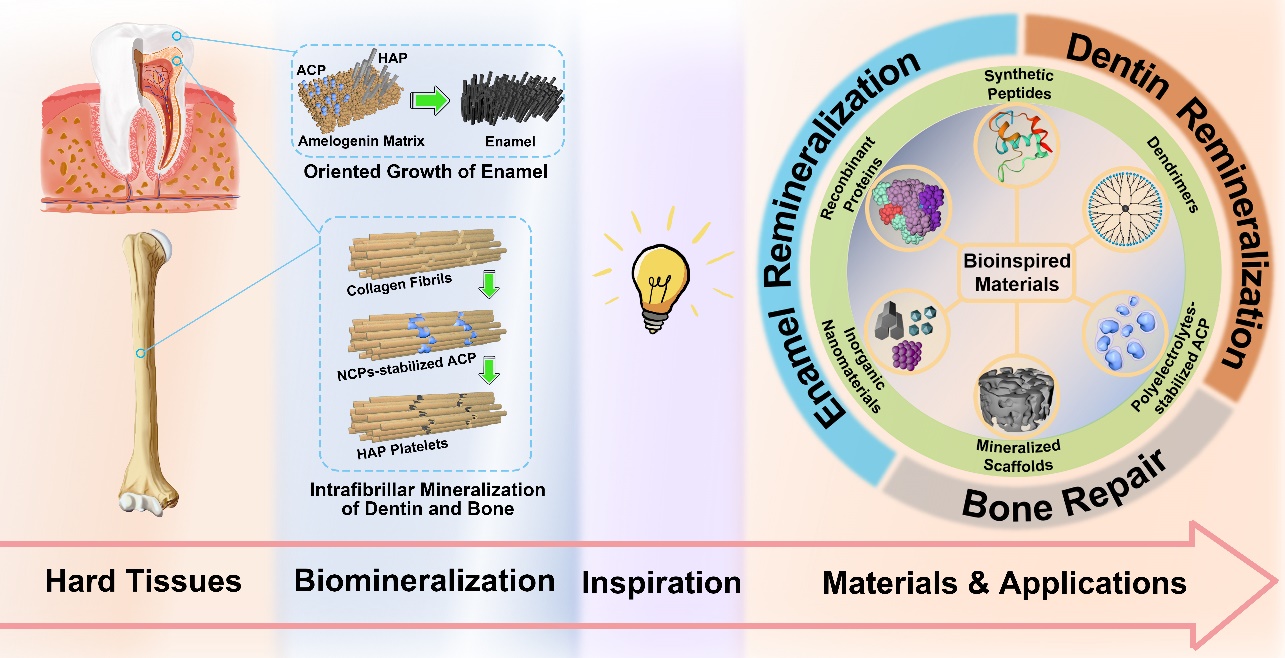


Fig. 1

Schematic of biomineralization-inspired materials for hard tissue repair. The formation processes and mechanisms of human hard tissues, including bones and teeth, have inspired the design and construction of various materials for hard tissue repair. These materials mainly include recombinant proteins, synthetic peptides, dendrimers, polyelectrolytes-stabilized mineral precursors, mineralized scaffolds, and inorganic materials. The applications of these materials are categorized into three parts to be introduced in this review: bone repair, dentin remineralization, and enamel remineralization.


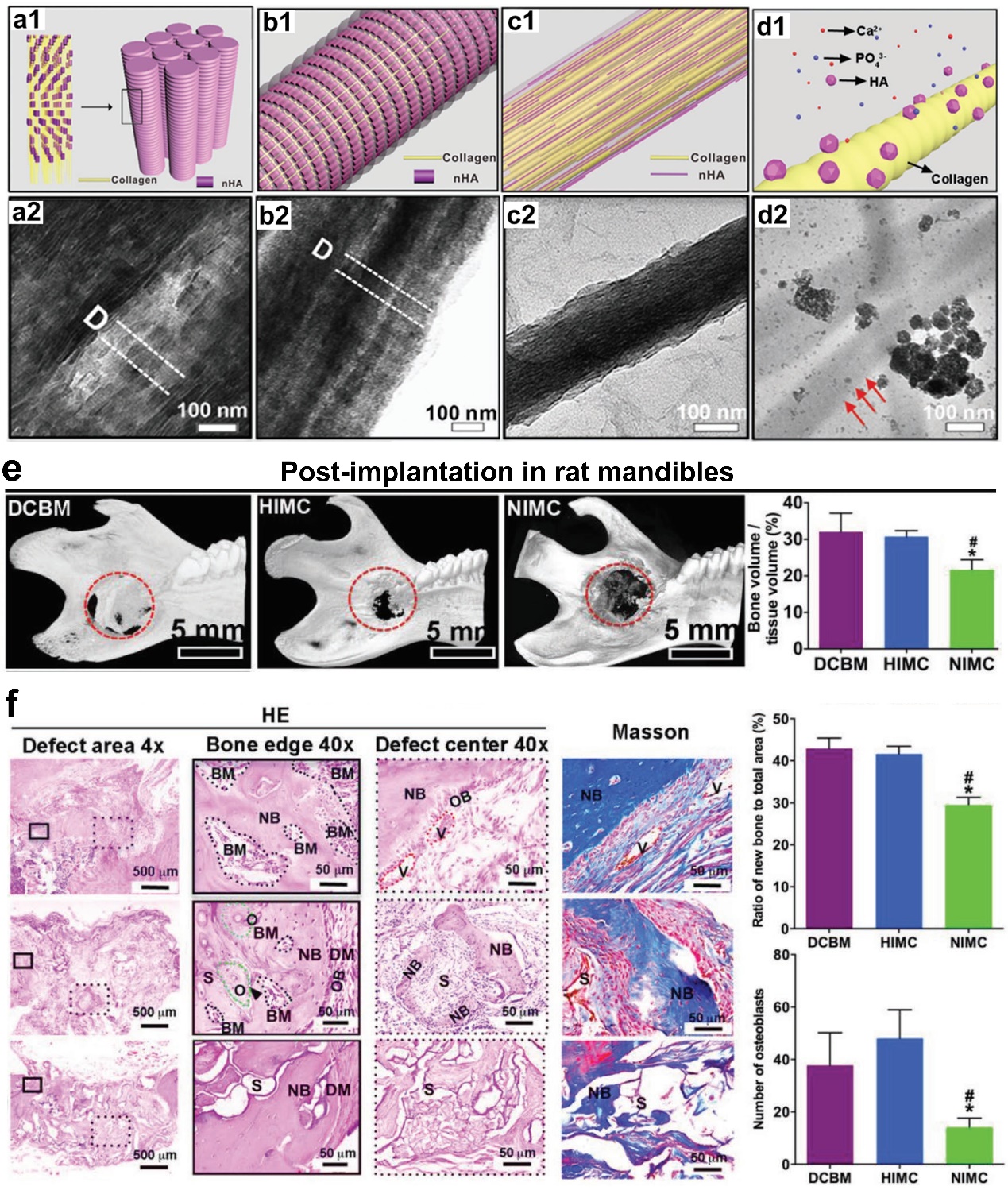


**Fig. 2** Schematics, characterizations, and *in vivo* effects of intrafibrillarly mineralized collagen scaffolds. Structural schematics of (a1) native bone, (b1) HIMC, (c1) NIMC, and (d1) EMC, respectively. TEM images of (a2) native bone, (b2) HIMC, (c2) NIMC, and (d2) EMC, respectively. The HIMC shows bone-like periodical nanoarchitectures, whereas the NIMC shows no periodicity. In the EMC, HAP clusters deposit randomly outside collagen fibrils. (e) Micro-CT images and bone volume of different scaffolds at 12 weeks post-transplantation in rat mandibles. The HIMC shows similar bone repairing effects with the DCBM. (f) H&E and Masson stainings, and semiquantitative analysis of regenerated bones in rat mandibles. The HIMC and the DCBM groups show abundant neo-bone formation and regeneration of osteoblasts and bone marrow, which are more than those of the NIMC group. Reproduced with permission from ref. [78](#_ENREF_78) 2019 Wiley-VCH.


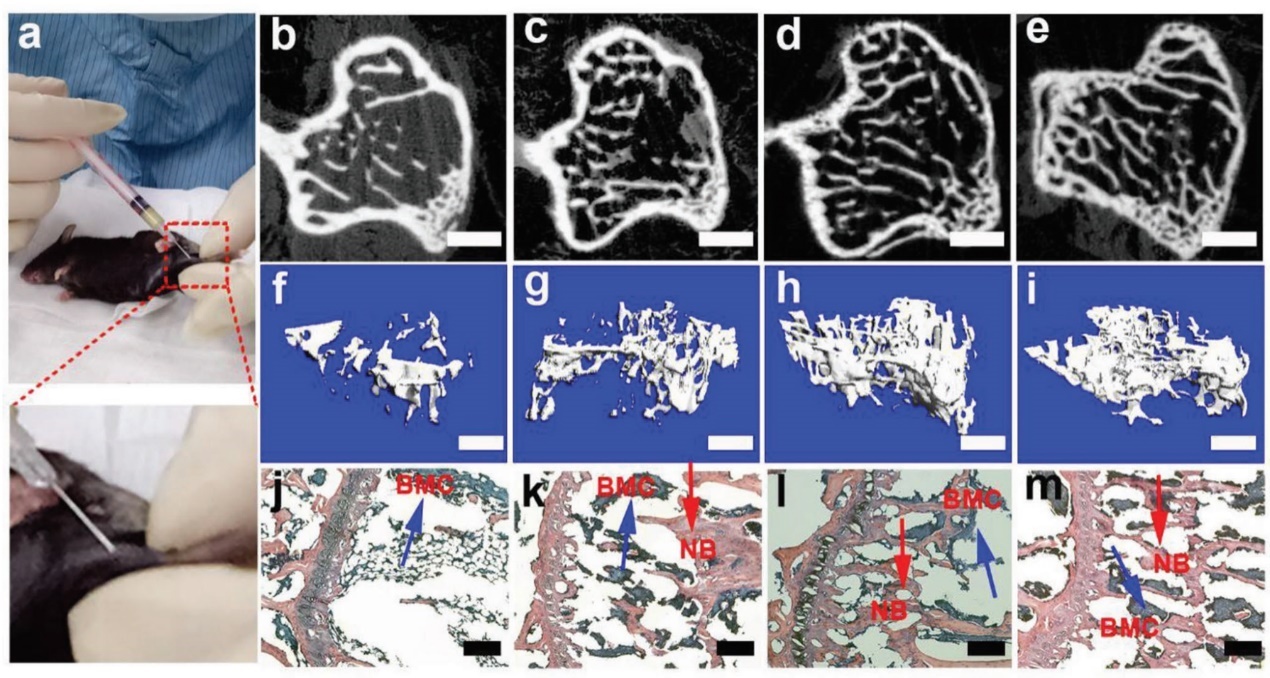


**Fig. 3** *In vivo* experiments of CaP-PILP on osteoporotic mouse. (a) Photographs of percutaneously injecting 30 μL of CaP-PILP into the osteoporotic tibia of mouse showing its mini-invasively injectability. (b-m) 2D, 3D micro-CT, and H&E staining of osteoporotic bone after the injection of CaP-PILP at (b, f, j) 0 weeks, (c, g, k) 4 weeks, (d, h, l) 8 weeks, and (e, i, m) 12 weeks. The CaP-PILP-recovered bone shows a significant new bone generation over time and it reaches the summit at 8 weeks. Scale bars: (b–e) 100 μm; (f–i) 300 μm; and (j–m) 200 μm. Reproduced from ref. 123 2019 Yao *et al*.


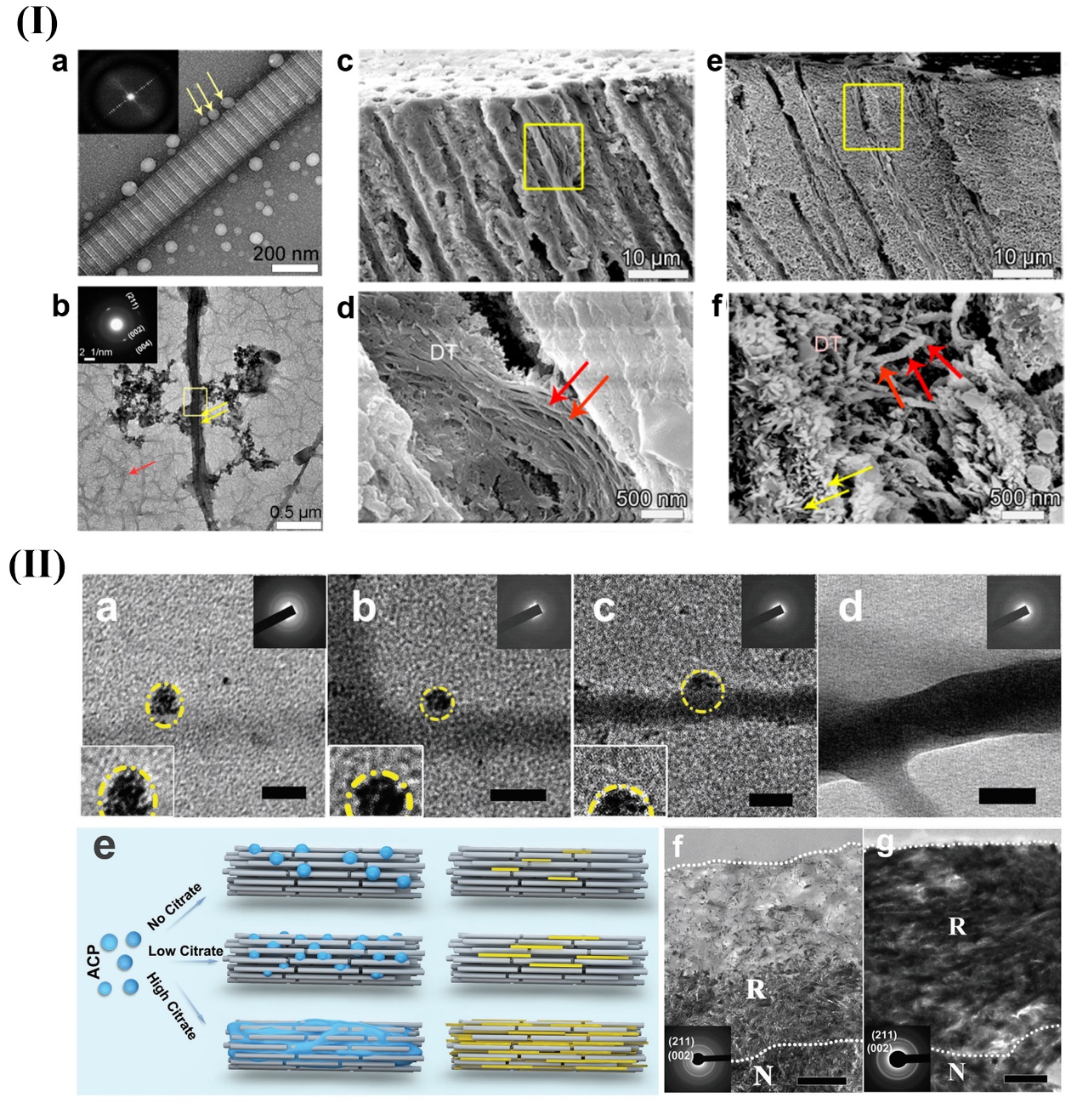


Fig. 4

Schematics and characterizations of biomineralization-inspired materials for dentin remineralization. (I) (a) TEM image of P26-collagen self-assembly. The assembled P26 peptides are dispersed nanospheres around the collagen fibril. (b) TEM image of collagen mineralization with P26 shows intrafibrillar mineralization and the inserted selected-area electron diffraction (SAED) image indicates the presence of HAP. Cross-sectional scanning electron microscopy (SEM) images of dentin after remineralization: (c, d) Control, and (e, f) with P26. By comparison, P26-treated dentin shows more apparent remineralization inside the dentinal tubules, which is reflected by a distinct string-of-beads morphology of the collagen fibrils. (II) (a-d) TEM images of ACP on collagen fibrils pretreated with different concentrations of citrate; (a) 0. (b) 25 × 10^–3^ _M_. (c) 50 × 10^–3^ _M_. (d) 100 × 10^–3^ _M_. These images indicate that citrate facilitates the infiltration of ACP into collagen fibrils. (e) Schematic of collagen mineralization *via* citrate pretreatment. Citrate decreases the contact angle and improves the wetting of ACP on collagen fibrils, and further promotes the degree of intrafibrillar mineralization. TEM images of remineralized dentin (f) without treatment and (g) with 100 × 10^–3^ _M_ citrate treatment. By contrast, citrate significantly promotes dentin remineralization. Scale bars: 50 nm (a-d), and 1 μm (f) and (g). (I) was reproduced with permission from ref. 145 2020 American Chemical Society. (II) was reproduced with permission from ref. 134 2018 Wiley-VCH


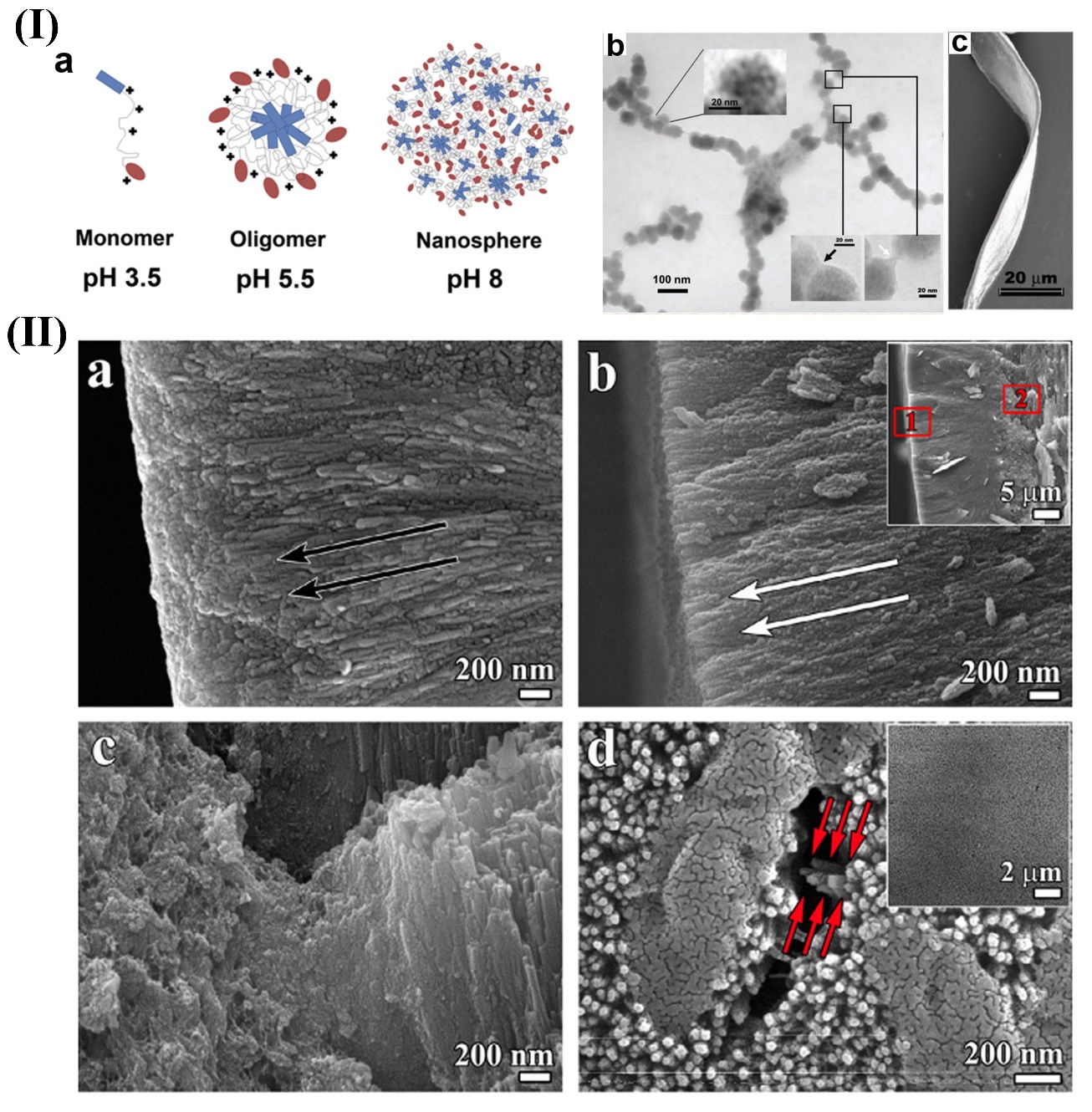


Fig. 5

Schematics and characterizations of amelogenin for enamel remineralization. (I) (a) Schematic of the formation of oligomer and nanosphere at different pH values. With the increase of pH, the amelogenin residues with positive charges are gradually deprotonated, thus the weak hydrophobic interactions lead to the formation of the nanospheres. (b) TEM images of the linear arrays of amelogenin nanospheres. (c) SEM image of a mature amelogenin ribbon showing well-defined edges. (II) (a) SEM images of native enamel, in which the arrows show the enamel orientation. (b-d) SEM images of newly formed crystal layer after the remineralization treatment with CS-AMEL hydrogel for 1 week. (b) Red rectangles 1 and 2 in the inserted image are selected regions in (b) and (c). White arrows exhibit the orientation of newly formed crystal layer. (c) The new layer is closely combined to the enamel surface. (d) Red arrows show the typical bundle of parallelly aligned crystals inside the new layer. The inserted image shows the homogenous surface of the new layer. (I) was reproduced with permissions from ref. 244 2011 American Society for Biochemistry and Molecular Biology, and ref. 214 2005 American Association for the Advancement of Science. (II) was reproduced with permission from ref. 264 2013 Elsevier.


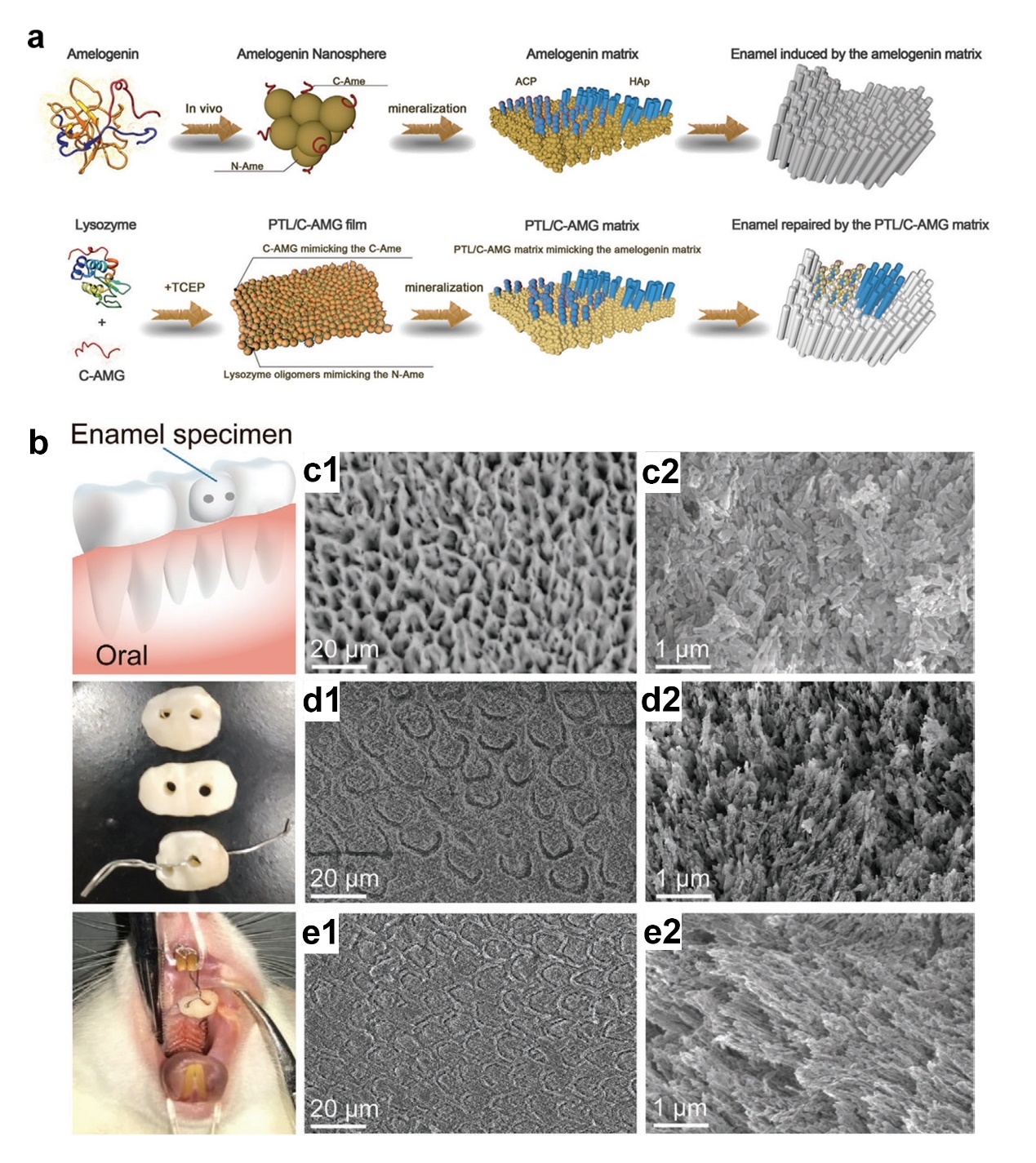


**Fig. 6** Schematic and *in vivo* experiments of PTL/C-AMG for enamel remineralization. (a) Schematic showing the similarity between amelogenin and PTL/C-AMG matrix in regulating the transition of ACP into HAP on enamel. (b) Schematic and photographs showing the process of fixing demineralized enamel slices in rat’s oral cavity. SEM images of (c) the untreated demineralized enamel, (d) demineralized enamel treated with fluoride, and (e) PTL/C-AMG film-treated demineralized enamel, after 2 weeks of remineralization in oral cavity. (c2), (d2), and (e2) are the corresponding high-magnification images of (c1), (d1), and (e1), respectively. The untreated group shows only incompact and irregular minerals. The fluoride group exhibits hollow cracks and irregular crystals. The PTL/C-AMG film group shows a “fish-scale” morphology that is similar to native enamel, and the newly formed crystals are highly oriented. Reproduced with permission from ref. 237 2020 Wiley-VCH


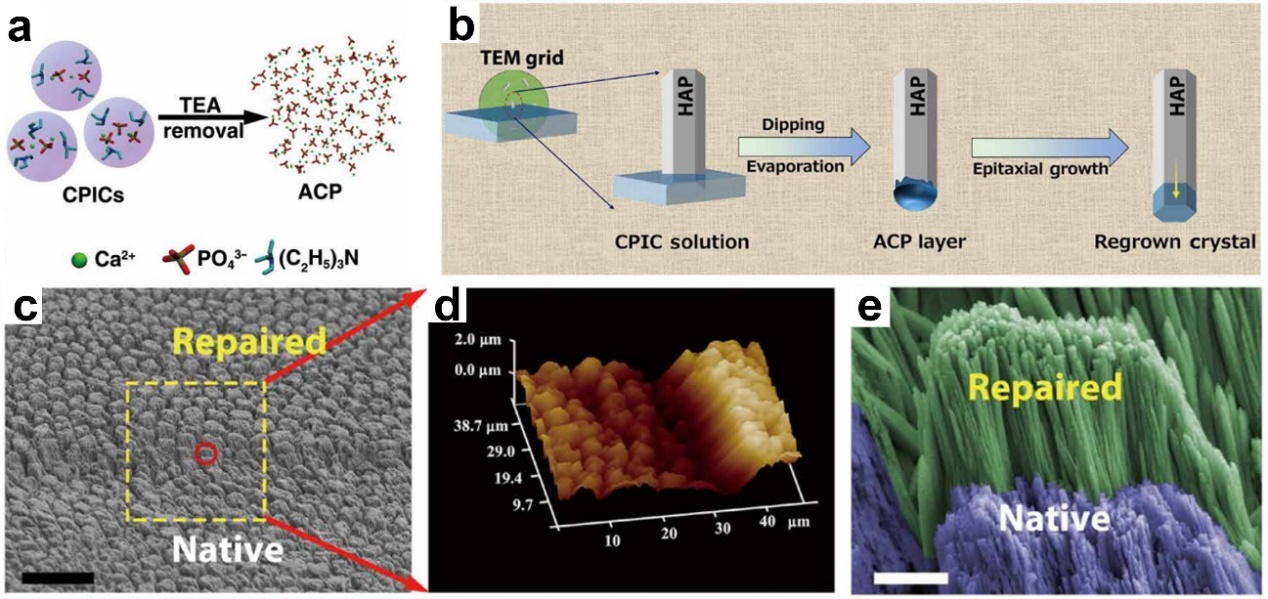


Fig. 7

Schematics and characterizations of CPICs for enamel remineralization. (a) Schematic of ACP formation: ACP is formed when the stabilizer (TEA) is removed from CPICs. (b) Schematic of the epitaxial growth of crystalline HAP. The coating CPICs solution transforms into an amorphous frontier on the HAP surface, and then transforms into HAP. (c) SEM image exhibiting CPICs-repaired enamel and acid-etched enamel. The repaired enamel shows a similar morphology to that of native enamel. (d) 3D atomic force microscopy (AFM) image of repaired enamel indicates the formation of a new HAP layer. (e) High-magnification SEM image of the red circle in (c), which shows the similar morphological texture between the repaired and native enamel. Scale bars: 20 μm (c), and 2 μm (e). Reproduced with permission from ref. 235 2019 American Association for the Advancement of Science.
