# Supplementary material for: Advances in biomineralization-inspired materials for hard tissue repair
Source: Int J Oral Sci. 2021 Dec 7;13:42. doi: 10.1038/s41368-021-00147-z (PMC8651686; doi:10.1038/s41368-021-00147-z)
Supplement: Supplementary file 2 — Summary of tables [file 41368_2021_147_MOESM2_ESM.docx]

**Table 1.** Representative biomineralization-inspired materials for bone repair

| Material | Approach | Characteristic | Model | Performance | Reference |
| --- | --- | --- | --- | --- | --- |
| Hierarchical intrafibrillarly mineralized collagen scaffold | PAA with a molecular weight of 2000 is added to regulate collagen mineralization | Native-bone like periodic nanoarchitectures | *In vivo* rat mandibular bone defect | Neo-bone formation, stem cell recruitment and differentiation, and regeneration of osteoblasts and bone marrow; M2 macrophage polarization | [^78^](#_ENREF_78)^,^[^79^](#_ENREF_79)^,^[^124^](#_ENREF_124) |
| Intrafibrillarly mineralized collagen scaffold | PAA and TPP are used to induce intrafibrillar mineralization; Fe^2+^ and Mn^2+^ are added. | Native-bone like periodic patterns; Fe/Mn-containing apatite | *In vitro* MC3T3 cell proliferation, and mouse BMSCs differentiation; *in vivo* mouse calvarial bone defect | Promote osteoblast adhesion and proliferation, and increase osteogenic-specific gene expression of BMSCs; bone regeneration, accumulation of osteoclasts in defect areas; high regeneration ratio and relative bone density | [^80^](#_ENREF_80) |
| Multilayered collagen scaffold | PAsp is used to induce intrafibrillar mineralization; layers are added by gelling collagen solution on the pre-formed mineralized/unmineralized collagen layer | Interconnected layers with sharp and well-defined interfaces, periodontium-like architecture | Not reported | Not reported | [^90^](#_ENREF_90) |
| Intrafibrillarly silicified collagen scaffold | PAH is used to induce intrafibrillar silicification | Intrafibrillar amorphous silica, ordered deposition of silica, mineralized banding patterns | *In vivo* subcutaneous implant in mouse; *in vivo* mouse calvarial bone defect; *in vivo* rat femoral bone defect; *in vitro* mice cell experiments | Biocompatible; promote *in situ* bone and vascular regeneration; promote monocytes differentiation and cytokines release to recruit BMSCs and EPCs | [^94^](#_ENREF_94)^,^[^95^](#_ENREF_95) |
| Biphasic silica/apatite co-mineralized collagen scaffold | Collagen scaffold is immersed in silicifying medium (containing PAH) and calcifying medium (containing PAsp), respectively in sequence | Intrafibrillar mineralization of both silica and apatite | *In vitro* cell experiments of mouse MSCs and macrophage-like RAW 264.7 cells | Enhance MSCs differentiation and inhibit  the differentiation of RAW 264.7 cells into osteoclasts | [^96^](#_ENREF_96) |
| Scaffold of collagen/apatite self-assembly | Slowly increase solution pH to induce the assembly of collagen molecules, and the apatite nucleation, simultaneously; requires neither NCPs nor their polymeric analogs | Intrafibrillar apatite | *In vivo* large-sized sheep cranial bone defect; *in vivo* rabbit rib defect; *in vitro* cell experiments of RAW 264.7 cells | Promote bone regeneration with notable osteoconductivity and osseointegration; enhance bone remodeling activity; promote M2 macrophage polarization | [^103-105^](#_ENREF_103) |
| CaP-PILP | PAA and PAsp are used to stabilize ACP | Injectable, moldable, and permeable | *In vivo* osteoporotic mouse tibia | Induce intrafibrillar mineralization, and promote osteoporotic bone recovery in a minimally invasive injection manner | [^123^](#_ENREF_123) |

*PAA* poly(acrylic acid), *TPP* sodium tripolyphosphate, *BMSCs* bone marrow mesenchymal stem cells, *PAsp* poly(aspartic acid), *PAH* poly(allylamine) hydrochloride, *EPCs* endothelial progenitor cells, *MSCs* mesenchymal stem cells, *CaP-PILP* calcium phosphate polymer-induced liquid-precursor

**Table 2.** Representative biomineralization-inspired materials for dentin remineralization

| Material | | Demineralization | Approach | Model | Performance | Reference |
| --- | --- | --- | --- | --- | --- | --- |
| Peptides | 8DSS peptide | 37% phosphoric acid, 2 min | 8DSS is coated on demineralized dentin by adding its solution; 1 mg/mL, 1 h | *In vitro* remineralization in artificial saliva for 3 weeks; *in vitro* cell experiment of human dental pulp cell | Good biocompatibility; good binding strength to dentin collagen; promote mineral regeneration and improve mechanical properties of demineralized dentin | [^132^](#_ENREF_132) |
|  |  | 37% phosphoric acid, 15 s |  | *In vitro* remineralization in artificial saliva for 4 weeks | Decrease dentin permeability; dentinal tubule occlusion | [^138^](#_ENREF_138) |
|  | DMP1-inspired peptides | 14% EDTA, 10 d; removal of non-collagenous proteins by the treatment of HCl and trypsin-EDTA | Immersion treatment in peptide solutions; 4%, 15 h | *In vitro* remineralization in wells supplied with calcium and phosphate buffer, for 2 weeks | Bind to demineralized human dentin; stabilize nucleation clusters; promote remineralization in collagenase-challenged dentin matrices | [^141^](#_ENREF_141) |
|  | Amelogenin-inspired peptide | Demineralizing solution (2 mM CaCl_2_·2H_2_O, 2 mM KH_2_PO_4_, 50 mM sodium acetate, and 0.05 M acetic acid), 3 d | Immersion treatment in peptide solution; 0.5 mg/mL, overnight | *In vitro* remineralization in artificial saliva for 10 days | Increase mineral density, promote tensile strength, hardness, and modulus of remineralized dentin | [^145^](#_ENREF_145) |
| PAMAM dendrimers | PAMAM–COOH | 37% phosphoric acid, 15 s; or 0.5 M EDTA, 30 min, 4 M guanidine chloride, 1 h | Immersion treatment in PAMAM–COOH solution; 10,000 ppm, 12 h | *In vitro* remineralization in artificial saliva; *in vitro* collagen mineralization; *in vivo* remineralization in the oral cavity of rats for 2 weeks | Promote intrafibrillar mineralization of demineralized dentin and collagen fibrils; induce remineralization in oral cavity, and promote the morphology and compactivity of newly generated minerals | [^133^](#_ENREF_133) |
|  | PAMAM–PO_3_H_2_ | 0.5 M EDTA, 30 min, 4 M guanidine chloride, 1 h | Immersion treatment in PAMAM–PO_3_H_2_ solution; 1,000 ppm, 12 h | *In vitro* cell experiments of HepG2 cells; *in vitro* remineralization in artificial saliva; *in vivo* remineralization in the oral cavity of rats | Low cell cytotoxicity; promote mineral regeneration *in vivo* and in oral cavity; promote surface microhardness recovery | [^158^](#_ENREF_158) |
| Polyelectrolytes-stabilized ACP | CaP-PILP | 37% phosphoric acid, 20 s | CaP-PILP is added into remineralization solution | *In vitro* remineralization in remineralization solution for 10 days | Induce both intrafibrillar and extrafibrillar remineralization | [^181^](#_ENREF_181) |
|  | PAH-ACP | 15% phosphoric acid, 15 s | PAH-ACP loaded mesoporous silica nanoparticles are sprinkled onto dentin surface and embedded with a resin | *In vitro* remineralization for 3 months; *in vitro* cell experiment on osteogenic differentiation of hMSCs | Induce heavily mineralization; promote osteogenesis of hMSCs | [^202^](#_ENREF_202) |

*8DSS* eight repetitive sequences of aspartic acid-serine-serine, *DPP* dentin phosphoprotein, *DMP1* dentin matrix protein 1, *EDTA* ethylenediaminetetraacetic acid, *PAMAM–COOH* carboxyl-terminated poly(amidoamine), *PAMAM–PO_3_H_2_* phosphate-terminated poly(amidoamine), *CaP-PILP* calcium phosphate polymer-induced liquid-precursor, *PAH-ACP* poly(allylamine) hydrochloride-stabilized amorphous calcium phosphate, *hMSCs* human mesenchymal stem cells

**Table 3.** Representative biomineralization-inspired materials for enamel remineralization

| Material | | Demineralization | Approach | Model | Performance | Reference |
| --- | --- | --- | --- | --- | --- | --- |
| Proteins and peptides | Amelogenin-containing chitosan hydrogel | 30% phosphoric acid, 30 s | The hydrogel is applied to enamel surface | *In vitro* remineralization in artificial saliva for 7 days | Stabilize calcium phosphate clusters, induce needle-like crystals formation, and improve the bonding between enamel and newly grown layer | [^264^](#_ENREF_264) |
|  | shADP5 peptide | White spot lesion: daily cycling between demineralization and neutral solutions for 6 and 17.5 h, respectively | Immersion treatment in peptide solution; 0.8 mM, 10 min | *In vitro* remineralization in Ca^2+^/PO_4_^3–^ solution for 1 h | Facilitate the formation of dense layer of HAP crystals, and incorporate fluoride ions into the remineralized layer | [^274^](#_ENREF_274) |
|  | Peptide-7 | 37% phosphoric acid, 30 s | The peptide-7 solution is dropped on enamel surface; 2.5 mg/mL, 10 min | *In vitro* remineralization in artificial saliva for 8 days; *in vivo* remineralization in rats with caries | Strong affinity to HAP; induce the formation of compact crystal layer; excellent cariogenic prevention effect comparable to fluoride | [^294^](#_ENREF_294) |
|  | Oligopeptide amphiphile | 37% phosphoric acid, 60 s | The oligopeptide amphiphile is added into mineralization solution; 15 μg/mL | *In vitro* remineralization in mineralization solution for 1 day or 20 days (1 mg/L NaF is contained in the mineralization solution) | Induce the formation of ACP nanoparticles; improve the packing density of newly formed crystal layer of remineralized enamel | [^308^](#_ENREF_308) |
|  | PTL/C-AMG | 37% phosphoric acid, 50 s, or 5 min to remove the outermost prism-like enamel crystals | *In vitro*: immersion treatment in a PTL/C-AMG buffer; 10 min. *In vivo*: PTL/C-AMG buffer is injected into the oral cavity of rats | *In vitro* remineralization in artificial saliva for 1 week; *in vivo* remineralization in rats’ oral cavity for 14 days | Induce regularly arranged enamel-like crystals with identical orientations, and restore mechanical strength to the level of natural enamel; induce enamel-like prisms in rats’ oral cavity | [^237^](#_ENREF_237) |
| PAMAM dendrimers | ALN–PAMAM–COOH | 37% phosphoric acid, 45 s | ALN–PAMAM–COOH is added onto enamel surface; 4 mg/mL | *In vitro* remineralization in artificial saliva for different periods; *in vitro* cell experiments of HepG2 cells, and L929 cells; *in vivo* remineralization in rats’ oral cavity | Low cytotoxicity; strong binding on enamel and facilitate nanorod-like crystal formation; promote enamel remineralization in rats’ oral cavity | [^219^](#_ENREF_219) |
|  | PAMAM–PO_3_H_2_ |  | PAMAM–PO_3_H_2_ is added onto enamel surface; 1 mg/mL |  |  | [^232^](#_ENREF_232) |
| Inorganic materials | Calcium phosphate ion clusters | 37% phosphoric acid, 30 s or 10 min to remove the prism-less enamel | CPIC ethanol solution is dropped onto enamel surface; 2 mg/mL | *In vitro* remineralization in modified simulated oral fluid for 48 h (15 ppm F^–^ is involved in the mineralization solution) | Induce epitaxial growth of enamel apatite, and recover hierarchical structure and mechanical properties to those of natural enamel | [^235^](#_ENREF_235) |
|  | Amorphous ZrO_2_ | 35% phosphoric acid gel, 20 s | Amorphous ZrO_2_ layer is coated on enamel through *in situ* growth | N/A | Recover mechanical properties; prevent bacterial adhesion and proliferation | [^228^](#_ENREF_228) |

*SAP* salivary acquired pellicle, *PTL/C-AMG* phase-transited lysozyme/C-terminus of the amelogenin peptide, *ALN–PAMAM–COOH* carboxyl-terminated PAMAM–alendronate conjugate, *PAMAM–PO_3_H_2_* phosphate-terminated poly(amidoamine), *CPIC* calcium phosphate ion cluster
