# Supplementary material for: Advances in biomineralization-inspired materials for hard tissue repair
Source: Int J Oral Sci. 2021 Dec 7;13:42. doi: 10.1038/s41368-021-00147-z (PMC8651686; doi:10.1038/s41368-021-00147-z)
Supplement: Supplementary file 3 — Manuscipt_Marked-up version [file 41368_2021_147_MOESM3_ESM.docx]

Advances in biomineralization-inspired materials for hard tissue repair

Running title: Biomineralization-inspired materials

Shuxian Tang^1^, Zhiyun Dong^1^, Xiang Ke^1^, Jun Luo^1,*^, and Jianshu Li^1, 2,*^

^1^College of Polymer Science and Engineering, State Key Laboratory of Polymer Materials Engineering, Sichuan University, Chengdu 610065, P.R. China.

^2^Med-X Center for Materials, Sichuan University, Chengdu 610065, P.R. China.

Correspondence: Jun Luo (luojuncd@scu.edu.cn) and Jianshu Li (jianshu_li@scu.edu.cn)

**Abstract**

Biomineralization is the process by which organisms form mineralized tissues with hierarchical structures and excellent properties, including the bones and teeth in vertebrates. The underlying mechanisms and pathways of biomineralization provide inspiration for designing and constructing materials to repair hard tissues. In particular, the formation processes of minerals can be partly replicated by utilizing bioinspired artificial materials to mimic the functions of biomolecules or stabilize intermediate mineral phases involved in biomineralization. Here, we review recent advances in biomineralization-inspired materials developed for hard tissue repair. Biomineralization-inspired materials are categorized into different types based on their specific applications, which include bone repair, dentin remineralization, and enamel remineralization. Finally, the advantages and limitations of these materials are summarized, and several perspectives on future directions are discussed.

1. Introduction

In living organisms, biomineralization produces materials with intricate structures and astonishing properties.[^1^](#_ENREF_1) The bones and teeth of vertebrates are hierarchically ordered at multiscale levels and possess remarkable mechanical properties,[^2^](#_ENREF_2) playing important roles in protection, movement support, and food mastication.[^3^](#_ENREF_3)^,^[^4^](#_ENREF_4) However, the hard tissues in human body (including bones, dentin, and enamel) have finite self-repair capabilities, especially when subjected to severe injuries and diseases such as large bone defects and dental caries.[^5^](#_ENREF_5)^,^[^6^](#_ENREF_6) In case of such health issues, various types of synthetic materials (including metals, ceramics, polymers, and composites) are used for clinical applications, although these markedly differ from biogenic hard tissues in some facets, such as composition, mechanical properties, bioactivities, and degradation behaviors.[^7-9^](#_ENREF_7) Despite native hard tissues inspire the development of advanced materials for hard tissue repair,[^10^](#_ENREF_10) the structures and functions of the native prototypes are too sophisticated to be directly realized *via* artificial synthesis.[^5^](#_ENREF_5)^,^[^11-13^](#_ENREF_11)

An in-depth understanding of the processes and mechanisms underlying biomineralization may provide novel approaches for hard tissue repair.[^4^](#_ENREF_4)^,^[^9^](#_ENREF_9)^,^[^14^](#_ENREF_14) Biomineralization is defined as the forming process of inorganic minerals by living organisms under strict biological control.[^12^](#_ENREF_12)^,^[^15^](#_ENREF_15) Different from mineralization, the concept of biomineralization emphasizes on the organisms’ regulation effects on the formation of biominerals. Through evolution, nature has established robust pathways and mechanisms to produce precisely controlled biominerals, which are distinct from minerals found in non-biological systems.[^16-18^](#_ENREF_16) Biomineral formation is a complex process involving frequent participation of cells and interactions between mineral forming crystals and various biomolecules, as well as ions.[^9^](#_ENREF_9)^,^[^17^](#_ENREF_17)^,^[^19-21^](#_ENREF_19) Many organic matrices also play crucial roles in regulating crystal nucleation, hierarchical growth, and morphogenesis.[^9^](#_ENREF_9)^,^[^12^](#_ENREF_12)^,^[^22^](#_ENREF_22) For example, the morphology of mineral crystals during enamel development is tightly regulated by matrix proteins;[^23^](#_ENREF_23) during collagen mineralization of dentin and bone, the negatively charged domains of non-collagenous proteins stabilize crystal precursors, while the self-assembled collagen fibrils act as templates to guide crystal growth.[^12^](#_ENREF_12)^,^[^24^](#_ENREF_24)^,^[^25^](#_ENREF_25)

Accumulating knowledge on biomineralization has facilitated the designing of strategies and materials for hard tissue repair. Notably, natural structure-forming processes can be partly realized *in vitro* by following the identified underlying mechanisms of biomineralization.[^26^](#_ENREF_26) Bioprocess-inspired fabrication strategies provide operable alternatives to most conventional biomimetic strategies, which only focus on directly mimicking the intricate structures or remarkable properties of natural hard tissues through artificial synthesis under harsh conditions.[^13^](#_ENREF_13)^,^[^27^](#_ENREF_27) Since several important proteins are intimately involved in the biomineralization of hard tissues, the mechanisms of their interactions with minerals and mineral precursors are widely studied. Using protein-mediated processes as inspiration, organics with special functions similar to those of the proteins have been artificially engineered to induce bioinspired mineralization; such organics include recombinant proteins, synthetic peptides, and polymers.[^13^](#_ENREF_13)^,^[^17^](#_ENREF_17)^,^[^28^](#_ENREF_28)^,^[^29^](#_ENREF_29) More importantly, laboratory evidence of *in vitro* bioinspired systems can contribute to the fundamental knowledge of biomineralization, such as the identification of amorphous precursors in vertebrate bones.[^10^](#_ENREF_10)

This review summarizes recent advances in the development of biomineralization-inspired materials for hard tissue repair (Fig. 1). To limit our focus, only existing studies on the mimicry of major hard tissue biomineralization processes are discussed in this review. To this end, we start with a brief introduction to the biomineralization of hard tissues in humans. Then, we propose the key requirements for biomineralization-inspired materials for hard tissue repair. Subsequently, we highlight bioinspired materials designed and utilized for bone repair, dentin remineralization, and enamel remineralization, and categorize them based on material types. Finally, we conclude the advantages and limitations of these materials, and discuss several perspectives on future research directions in the context of biomineralization.

2. Bone repair

Bone is responsible for the maintenance of mineral homeostasis, structural support, and protection of the human body.[^30^](#_ENREF_30) Its superb mechanical properties are conferred through hierarchical organization, which ranges from nanoscale structures to macroscopic physiological structures.[^31^](#_ENREF_31)^,^[^32^](#_ENREF_32) The building blocks of bone are mineralized collagen fibrils, which are known for the complicated nanostructures.

Synthetic bone grafts with bone-like structures and properties are difficult to prepare. Nevertheless, the biomineralization of bone has provided inspirations for material design and construction. The review by Habibovic *et al.*[^10^](#_ENREF_10) systematically elaborates bioinspired materials mimicking the extracellular matrix of bone, and the authors focus on the discussion and comparison of several important physiochemical properties of the materials, such as the mineral content and the homogeneity of mineral distribution, in scaffolds.

In contrast, the subject of our review focuses on the relationship between the mechanisms of biomineralization and the design and construction of materials for hard tissue repair. Therefore, this section starts with the backgrounds of biomineralization of bone, which involve frequently mentioned mechanisms and processes during biomineralization. Then, several key requirements for biomineralization-inspired materials for bone repair are presented by analyzing advantages and disadvantages of conventional materials, especially for mineralized collagen. Finally, we summarize the advances in biomineralization-inspired materials for bone repair, and some important information of representative materials, including construction approach, characteristics, and biological performances, are listed in Table 1.

2.1. Biomineralization of bone

Biomineralization of bone is defined as the formation of mineral crystals in the extracellular matrix of bone tissues. There are several specialized reviews about biomineralization of bone.[^19^](#_ENREF_19)^,^[^33^](#_ENREF_33)^,^[^34^](#_ENREF_34) Differently, the focus of this section is to emphasize the relationships between the design and construction of materials for bone repair and several main processes and mechanisms involved in biomineralization. The content includes collagen fibrils and nanocrystals, amorphous precursor, and intrafibrillar mineralization. By analyzing and sorting out the underlying mechanisms of these biological events, we hope to provide guiding principles and suggestions for material design and construction.

2.1.1. Collagen fibrils and nanocrystals

In bone, there is an apparent contrast between the minerals inside and outside the collagen fibrils. The intrafibrillar minerals are plate-shaped nanocrystals with their *c*-axes oriented along the longitudinal axes of the fibrils; while the extrafibrillar minerals are crystal aggregates without preferential orientation.[^35^](#_ENREF_35) Such differences derive from the spatial confinement effect of assembled collagen fibrils on the growth of intrafibrillar minerals. Before mineralization, the fully assembled three-dimensional collagen fibrils possess a regular series of gaps or channels due to the staggered arrangement of adjacent collagen molecules.[^36^](#_ENREF_36) The mineral crystals initially nucleate in the holes of these gap zones and then grow into plate-shaped nanocrystals with typical dimensions of 50 × 25 × 3 nm.[^5^](#_ENREF_5)^,^[^37^](#_ENREF_37) The ordered fibrillar structure and intrafibrillar nanocrystals contribute prominently to the remarkable mechanical properties of bone.[^38^](#_ENREF_38)

Therefore, it is of crucial importance to replicate the fibrillar template structure of the naturally assembled collagen molecules when designing and constructing materials for bone repair; besides, the regulation of mineral size and location should be considered carefully.

2.1.2. Amorphous precursor

The nucleation and growth of mineral crystals in bone do not follow the classical crystallization pathway of ion addition; instead, there is a transformation from amorphous calcium phosphate (ACP) to the final crystalline hydroxyapatite (HAP).[^39-43^](#_ENREF_39) Since mechanisms about initiation, stabilization, and transformation of the amorphous precursor have been well presented and discussed by several illuminative reviews,[^9^](#_ENREF_9)^,^[^44-46^](#_ENREF_44) the focus here will be given to the features of amorphous phase which can shed light on material design and construction.

One question may be proposed at first: what are the prominent advantages of the amorphous phase strategy taken by numerous organisms during evolution. It can be answered from three aspects. First, the highly hydrated amorphous phase possesses liquid-like fluidity and moldability, which are believed to be the foundations of structuring sophisticated minerals, especially in extremely tiny compartments.[^47^](#_ENREF_47) Second, compared with ion solutions, ACP is a concentrated phase of calcium and phosphate, which is more efficient in mineralization and more convenient in delivery.[^43^](#_ENREF_43) Third, ACP is more soluble than HAP, thus having more flexible and controllable properties in reorganization and fusion.[^14^](#_ENREF_14)^,^[^48^](#_ENREF_48)

These advantages of distinct aspects can be transplanted separately or as a whole, into materials, depending on the main aims of material construction. More importantly, knowing the significance of implementing the amorphous precursor strategy in a specific material is necessary, rather than following research hot spots blindly, since some studies with ambiguous significances and unclear logics have been reported in material field.

2.1.3. Intrafibrillar mineralization

Over the last decade, mechanisms on the intrafibrillar mineralization of bone, especially the explanations of how minerals enter the interstices of collagen fibrils, have been continuously proposed. However, most of them are based on *in vitro* models due to the limited applicability of truly biological systems. In a landmark review, Tay *et al.*[^49^](#_ENREF_49) critically commented several well-known mechanisms, including capillary forces, electrostatic interaction, size exclusion, and self-assembly of collagen and apatite; later in 2016, they discovered a novel mechanism on intrafibrillar mineralization from the perspective of Gibbs-Donnan equilibrium.[^50^](#_ENREF_50) Very recently, With *et al.*[^51^](#_ENREF_51) thoroughly summarized various approaches of *in vitro* mineralization of collagen in their detailed review; besides, many experimental parameters and effects of these approaches were kindly listed in tables. These two reviews are highly recommended to readers who expect a comprehensive overview of intrafibrillar mineralization.

The realization methods of intrafibrillar mineralization are constantly innovating. However, there are contradictory explanations among the mechanisms these methods rely on, which may cause confusion to researchers trying to develop novel mineralized collagen materials.[^52^](#_ENREF_52)^,^[^53^](#_ENREF_53) Therefore, the essence underlying diverse methods should be reminded; here, we will give a brief analysis. All the methods can be roughly categorized into two types. One is to form a transiently stable mineral precursor to achieve its infiltration into collagen fibrils, and subsequently the precursor transforms into the crystalline phase to complete mineralization.[^42^](#_ENREF_42) Adding polyelectrolytes to stabilize ACP is the most common method to form a mineral precursor. In the earliest studies, anionic polyelectrolytes such as poly(aspartic acid) (PAsp) were utilized to simulate the regulation capability of non-collagenous proteins (NCPs) on minerals.[^54-56^](#_ENREF_54) NCPs are rich in acidic residues of amino acid exhibiting strong affinity towards calcium ion.[^5^](#_ENREF_5)^,^[^57^](#_ENREF_57) Later, cationic polyelectrolyte, poly(allylamine) hydrochloride (PAH) was also demonstrated to be able to stabilize ACP.[^50^](#_ENREF_50)^,^[^58^](#_ENREF_58) In addition to adding polyelectrolytes, the recently reported method of applying periodic fluid shear stress is also based on the principle of forming and stabilizing ACP.[^59^](#_ENREF_59)^,^[^60^](#_ENREF_60) Another type of method relies on the simultaneous occurrence of apatite growth and assembly of collagen molecules.[^61-63^](#_ENREF_61) In fact, the common requirement for using in vitro methods is the necessity for the mineral phase to enter the assembled collagen fibrils before it grows too large and sets in shape.^[19](#_ENREF_19)^

Researchers are better not to be limited to the guidance of certain mechanism when designing mineralized collagen materials, because the current mechanisms are not always perfect and still under debate.[^49^](#_ENREF_49) Instead, researchers should follow the essence of achieving intrafibrillar mineralization, carefully refer to various mechanisms, and constantly pursue methodological innovation and effect enhancement, which in turn will contribute to the improvement of mechanisms.[^64^](#_ENREF_64)^,^[^65^](#_ENREF_65)

2.2. Requirements for biomineralization-inspired materials for bone repair

Bone grafts are usually utilized to treat bone defects or guide new bone generation.[^66-68^](#_ENREF_66) Although natural bone autografts and allografts are the gold-standard treatment, they are limited to problems such as additional donor-site morbidity and disease transmission.[^69^](#_ENREF_69)^,^[^70^](#_ENREF_70) Synthetic grafts based on polymers, bioceramics, metals, and carbon materials are also unsatisfactory in some aspects, such as mechanical properties, degradation, and bioactivities.[^71^](#_ENREF_71) Advantages and disadvantages of these diverse materials for bone tissue engineering are available in the review by Mikos *et al*.[^7^](#_ENREF_7) Here, we will focus on discussing mineralized collagen scaffolds, which are closely related to the biomineralization subject of this review.

Among a variety of synthetic bone grafts, mineralized collagen scaffolds have the most similar composition with the extracellular matrix of native bone.[^72^](#_ENREF_72) Two conventional methods are usually applied to fabricate composite scaffolds of collagen and minerals. The first is to directly blend minerals and collagen solution; the second is to immerse preformed collagen scaffolds in the solutions of mineral ions.[^73-75^](#_ENREF_73) However, these collagen scaffolds are characterized by extrafibrillar mineral deposition and the lack of intrafibrillar mineral, thus failing to replicate the nanostructure of native bone.[^69^](#_ENREF_69)^,^[^76^](#_ENREF_76)^,^[^77^](#_ENREF_77) Consequently, these scaffolds usually provide insufficient mechanical strength, variable degradation rates, and limited osteoinductivity.[^78-80^](#_ENREF_78) It is not sufficient to simulate only the bone composition, since the structures of bone count a lot.^[81-83](#_ENREF_81)^

Biomineralization of vertebrates involves ingenious combinations of collagen fibrils and apatite with fine geometry and architecture, which inspires researchers to take similar mineralization methods to recapitulate bone structures in materials for improved properties.^[13](#_ENREF_13)^ However, the practicability of construction methods depends on their feasibility and the overall properties of the obtained materials. Therefore, there are several key requirements for biomineralization-inspired materials for bone repair need to be considered. First, biocompatibility of the materials. The fabrication process may involve chemical agents, whose fate after the implantation of materials in bone defect site has an impact on the overall biocompatibility.[^84^](#_ENREF_84)^,^[^85^](#_ENREF_85) Biocompatible agents are more preferable when constructing the materials. Second, structural similarity. The materials should try to replicate the structure of bone, in particular, the intrafibrillar mineralization. The regulation on material structures is likely to change the distribution and content of minerals, and their interactions with scaffold matrix, thereby greatly influencing the final properties.[^31^](#_ENREF_31)^,^[^32^](#_ENREF_32)^,^[^86^](#_ENREF_86) Characterizations on material structures should be always conducted to determine their mineralization patterns. Third, the construction method of recreating bone nanostructures in materials should be easy, and the cost should be as low as possible. Such method is very promising for the scalable production of bone repair materials.

2.3. Biomineralization-inspired materials for bone repair

2.3.1. Intrafibrillarly mineralized collagen scaffolds

Since Gower *et al.*[^87^](#_ENREF_87)^,^[^88^](#_ENREF_88) first realized the intrafibrillar mineralization of calcium carbonate in collagen sponges by using poly(acrylic acid) (PAA) in 2003, the concept of utilizing anionic polyelectrolytes to simulate the regulation of NCPs on minerals for collagen mineralization has been widely developed. Later, in the same way, calcium phosphate was demonstrated to achieve the intrafibrillar mineralization of collagen sponges, which indicates the invention of a bone-like material that is similar to bone in both composition and nanostructures.[^42^](#_ENREF_42)^,^[^85^](#_ENREF_85) Subsequently in 2008, Tay *et al.*[^56^](#_ENREF_56) reported the intrafibrillar remineralization of demineralized dentin, which can be seen as a three-dimensional collagen network scaffold. In brief, as the additive during mineralization process, polyelectrolytes play the role of stabilizing amorphous mineral precursors, which then are capable of penetrating into the gaps inside collagen fibrils and transforming into nanocrystals.[^44^](#_ENREF_44)^,^[^89^](#_ENREF_89)

Until recently, biomineralization-inspired analogs of NCPs have been utilized to construct intrafibrillarly mineralized collagen scaffolds for bone repair. For instance, a hierarchical intrafibrillarly mineralized collagen (HIMC) scaffold was prepared by precisely controlling the molecular weight of added poly(acrylic acid) (PAA); PAA was utilized as the NCPs analog for facilitating intrafibrillar mineralization.[^79^](#_ENREF_79) HIMC had a staggered nanostructure with light- and dark-contrast zones, as observed in transmission electron microscopy (TEM) images, similar to those in native bone; however, such zones could not be observed in non-hierarchical intrafibrillarly mineralized collagen (NIMC) and extrafibrillarly mineralized collagen (EMC) (Fig. 2: a-d). *In vitro* cell experiments showed that HIMC scaffold established a suitable microenvironment for multidifferentiation and cell-homing, similar to that in natural decellularized bone matrix (DCBM) scaffold; this was not observed in case of NIMC scaffold. Indeed, the bone-like staggered architecture and the nanometer-scale topography of HIMC scaffold facilitated cell-homing and multidifferentiation. Further, rat critical-sized mandibular bone defects were established as an *in vivo* model to evaluate and compare the bone repair efficacies of HIMC, NIMC and DCBM scaffolds (Fig. 2: e and f). After twelve weeks of implantation, bone defects were almost entirely repaired and neo-bone formation and bone marrow regeneration were observed in HIMC group, comparable to those in DCBM group. HIMC recruited bone marrow mesenchymal stem cells, whose differentiation may promote osteogenesis and angiogenesis of neo-bone. A further study showed that the impact of the molecular weight of PAA on the resulted mineralization patterns may be related to the Gibbs free energy of the PAA-Ca^2+^ intermediates.[^78^](#_ENREF_78) However, this inference is merely based on theoretical calculations and more experimental data are required to support it. The studies[^78^](#_ENREF_78)^,^[^79^](#_ENREF_79) on HIMC scaffold provide an idea for finely tuning the nanostructures of intrafibrillarly mineralized collagen scaffolds, and demonstrate its treatment potential as a bone repair scaffold. However, it should be noted that the preparation process of HIMC scaffold requires a long time, which may be a hurdle to its practical application.

In addition, multilayered collagen scaffolds can be prepared in a similar way. Sone *et al.*[^90^](#_ENREF_90) introduced poly(aspartic acid) (PAsp) into the mineralization medium to stabilize ACP and achieved intrafibrillar mineralization of bulk collagen gels. Based on a hypermineralized collagen gel layer, a trilayered collagen scaffold was fabricated by incorporating an unmineralized intermediate layer and a thin mineralized top layer. The layers were interconnected, and the degree of mineralization could be controlled by the mineralization duration. The trilayered scaffold was intended to mimic the complex architecture of the periodontium, which is composed of bone, periodontal ligament, and cementum. Although this study achieved layer-dependent intrafibrillar mineralization in an integrate multilayered scaffold for the first time, further studies are needed to evaluate the *in vivo* effects on periodontium regeneration of this scaffold.

In addition to apatite, silica is an inorganic phase that can infiltrate collagen fibrils. Tay *et al.* first reported the achievement of intrafibrillar silicification of collagen.[^91-93^](#_ENREF_91) A precursor phase of amorphous silica induced by poly(allylamine) hydrochloride (PAH) was highly similar to fluidic liquids, facilitating the entry and filling of the interstices within collagen fibrils. The cross-banding patterns and microfibrillar architecture of native fibrillar collagen were well replicated in silicified collagen. Moreover, the mechanical strength of the silicified collagen sponge was remarkably enhanced over the highly compliant non-silicified sponge.[^91^](#_ENREF_91) In recent years, the strategy of intrafibrillar silicification has been harnessed to prepare mineralized collagen scaffolds for bone regeneration.[^94-97^](#_ENREF_94) For example, Tay *et al.* prepared an intrafibrillarly silicified collagen scaffold that promoted osteogenesis and angiogenesis through monocyte immunomodulation.[^95^](#_ENREF_95) The silicic acid released from the silicified collagen scaffold was able to stimulate monocytes differentiation to improve the expression of several cytokines, thus promoting the homing of bone marrow mesenchymal stem cells (BMSCs) and endothelial progenitor cells. Studies on intrafibrillarly silicified collagen scaffold indicate that calcium phosphate is not the only choice of mineral phase, despite calcium phosphate is a main component of bone. Intrafibrillar mineralization of calcium carbonate[^88^](#_ENREF_88), biphasic silica/apatite[^92^](#_ENREF_92)^,^[^96^](#_ENREF_96), and yttria-stabilized zirconia[^98^](#_ENREF_98), towards collagen scaffolds has been reported, while biological effects on bone repair of these scaffolds are generally not studied. Owing to the different bioactivities derived from different minerals,[^7^](#_ENREF_7)^,^[^80^](#_ENREF_80) the intrafibrillar mineralization of multiple mixed minerals may be a future direction of realizing multifunctional bone repair scaffolds; however, controls over the distribution and content of diverse minerals inside collagen fibrils remain huge challenges.

Another strategy to fabricate mineralized collagen scaffolds is based on the collagen/apatite self-assembly.[^61-63^](#_ENREF_61)^,^[^99^](#_ENREF_99)^,^[^100^](#_ENREF_100) In brief, increased pH initiates fibrillogenesis in acidic collagen solution, which leads to crystal nucleation in the gap zones.[^61^](#_ENREF_61) The self-assembled collagen fibrils act as templates guiding the growth and orientation of apatite crystals, resulting in nano-sized bone-like crystals that are uniformly embedded in collagen matrices.[^62^](#_ENREF_62) Unlike the strategies described previously for intrafibrillar mineralization, the method of collagen/apatite self-assembly requires neither NCPs nor their polymeric analogs. Moreover, this method allows the use of a much higher concentration of collagen solution, making it suitable for preparing mineralized scaffolds for bone tissue engineering.[^49^](#_ENREF_49)^,^[^61^](#_ENREF_61) Cui *et al.* systematically studied the physiochemical properties, biodegradability, biocompatibility, and clinical effects of the mineralized collagen scaffold, prepared *via* the collagen/apatite self-assembly method.[^101-107^](#_ENREF_101) They found that the composition and microstructure of the mineralized collagen scaffold were similar to those of the natural bone matrix. Furthermore, they expanded the mineralized collagen scaffold by incorporating it with polymers,[^108-112^](#_ENREF_108) minerals,[^113^](#_ENREF_113) growth factors[^114^](#_ENREF_114) or cells[^115^](#_ENREF_115) to improve its mechanical properties or bioactivities. The excellent biocompatibility and osteogenic ability of the mineralized collagen scaffold enabled its outstanding clinical efficacy in the treatment of bone defects and diseases caused by various factors.[^101^](#_ENREF_101)^,^[^116-118^](#_ENREF_116)

2.3.2. Intrafibrillarly mineralized non-collagenous composites

Developing non-collagenous polymers that can serve as templates for intrafibrillar-like mineralization is a potential strategy to obtain novel bone graft substitutes.[^10^](#_ENREF_10) Using this approach, the nanostructure and mechanical properties of native bone can be partly replicated, meanwhile, the replacement of collagen with certain polymers can avoid the immunogenicity of allogenic and xenogenic collagen products as well as remarkably reduce the cost.

Recently, several polymers[^77^](#_ENREF_77)^,^[^119-122^](#_ENREF_119) have been developed with the assistance of bioinspired mineralization methods to replicate the nanoscale organic-inorganic component organization in bone. For instance, Aparicio *et al.*[^120^](#_ENREF_120) achieved intrafibrillar mineralization of self-assembled elastin-like recombinamers (ELRs) utilizing a bioinspired polymer-induced liquid precursor mineralization method. ELRs were disordered at low temperatures, while self-assembled into nanofibrils when the temperature exceeded the inverse transition point. Fluid-like PAsp-stabilized ACP was able to infiltrate self-assembled ELR fibrils, and transform into HAP nanocrystals with highly oriented alignment guided by ELR fibrils, resembling that in bone. Similarly, Tang *et al.*[^122^](#_ENREF_122) reported a biomineralization-inspired technique for the synthesis of functional polyvinyl alcohol/sodium alginate/hydroxyapatite (PVA/Alg/HAP) macrofiber. Ultrasmall amorphous CaP nanocluster precursors were dispersed in the hybrid film of PVA and alginate, and then transformed into HAP through oriented crystallization during the process of uniaxial wet drawing. A hierarchical order was formed owing to the adjusted orientation and configuration of the polymer chains and HAP nanocrystals after adaptation to the stretching stress. The bone-like structure of the PVA/Alg/HAP macrofiber endowed it with spider silk-like super-toughness.

The studies on intrafibrillarly mineralized non-collagenous composites indicate that fibrillar polymers have a template function similar to collagen fibrils, and these fibrillar polymers are capable of inducing *in vitro* mineralization to construct bone-like materials. Follow the same method, other fibrillar polymers and other minerals may also be able to form intrafibrillarly mineralized composites, which can be a future pathway to increase the diversity and functionality of bone scaffolds. However, current studies mainly focus on the characterization of materials and are lack of biological researches, especially on the bone repair effects.

2.3.3. Polymer-induced liquid precursor of calcium phosphate

Unlike large bone defects, osteoporosis is a chronic disease characteristic of the shortage of minerals in bones. Polymer-induced liquid precursor (PILP) of calcium phosphate is injectable and contains mineral precursors, making it suitable for directly mineralizing osteoporotic bone *in vivo*. Tang *et al.*[^123^](#_ENREF_123) improved the conventional method of PILP by increasing the concentration of mineral ions and polymeric additives, resulting in a free-flowing calcium phosphate PILP material (CaP-PILP) that was viscous and transparent on a large scale. Plenty of uniformly sized ACP nanoclusters were observed in CaP-PILP. The good fluidity of CaP-PILP and the tiny sizes and high contents of its mineral precursors made it efficient in infiltrating and mineralizing osteoporotic bone. *In vivo* experiments (Fig. 3) performed in ovariectomized osteoporotic mouse tibia revealed that the bone recovered by CaP-PILP exhibited satisfactory mechanical strength comparable to that of healthy bone. In addition, the fluid-like properties of CaP-PILP enable it to be injected in a minimally invasive manner, thus avoiding surgical incision.

CaP-PILP provides a new concept for the treatment of osteoporosis. However, when the volume of the osteoporotic site is large, it is not clear whether CaP-PILP can penetrate the deep interior is still unclear, and larger animal model is required to demonstrate this point. Besides, whether the high concentrations of polymeric additives will be toxic to bone tissue cells remains to be determined.

3. Dentin remineralization

Human dentin is mainly composed of collagen and minerals. The progression of caries lesions into dentin in pathological conditions causes the mineral phase of dentin to be quickly dissolved by acids.[^124^](#_ENREF_124) After the mineral loss of dentin, collagen fibers are exposed to and degraded by endogenous enzymes, which remarkably impairs the mechanical properties of dentin.[^125^](#_ENREF_125) According to their location, the minerals can be categorized as intrafibrillar and extrafibrillar. Intrafibrillar mineralization determines the mechanical properties of dentin at the nanoscale level.[^38^](#_ENREF_38)^,^[^126^](#_ENREF_126) Bioinspired materials have made breakthroughs in inducing intrafibrillar mineralization of dentin by drawing lessons from dentin biomineralization. Efforts have been made to mimic NCPs that are intimately involved in the regulation of mineral formation and precursor stabilization during biomineralization. In this section, we describe the biomineralization of dentin, and the requirements for biomineralization-materials for dentin remineralization are proposed. Subsequently, we overview the bioinspired materials utilized as analogs of NCPs for dentin remineralization, including protein-inspired peptides, poly(amido amine) dendrimers, and amorphous precursors stabilized by polyelectrolytes. The biological performances of some representative materials are listed in Table 2.

3.1. Biomineralization of dentin

Dentin formation involves a series of sequentially synchronized biological events, including odontoblast differentiation, formation of mantle dentin, mineralization of primary dentin, and secretion of secondary and tertiary dentin.[^127^](#_ENREF_127) Odontoblasts are responsible for the production and secretion of unmineralized collagen, proteoglycans, and NCPs,[^128^](#_ENREF_128) including dentin sialophosphoprotein, and dentin matrix protein 1.[^129^](#_ENREF_129) Owing to the abundant carboxylic acid and phosphate functional groups in NCPs, they serve as preferential sites for mineral nucleation and subsequent crystallization.[^125^](#_ENREF_125) Although they account for less than 10% of the organic matrix of dentin, NCPs actively promote and regulate intrafibrillar mineralization of collagen fibrils.[^130^](#_ENREF_130)

The toughness of dentin is attributed to its structure, which is very similar to that of bone at the nanoscale and is characterized by the highly organized arrangement of nanosized minerals within collagen fibrils.[^32^](#_ENREF_32) Although the minerals in dentin can be categorized as intrafibrillar and extrafibrillar according to their location, it should be noted that the intrafibrillar minerals contribute prominently to the mechanical properties of dentin.[^38^](#_ENREF_38)^,^[^126^](#_ENREF_126) In addition, intrafibrillar mineralization protects collagen molecules from external challenges.[^125^](#_ENREF_125)

The main components of dentin and bone are very similar, and the biomineralization processes of them are similar, too. Introduction and analyses of the main mechanisms of collagen biomineralization can be referred to the previous content, “*Biomineralization of bone*”.

3.2. Requirements for biomineralization-inspired materials for dentin remineralization

Remineralization of demineralized dentin is more complex and less effective than that of enamel because of the limited availability of residual crystal seeds, especially when considering completely demineralized dentin.[^124^](#_ENREF_124)^,^[^131^](#_ENREF_131) Traditional remineralization methods focus on the supplementation of calcium and phosphate ions to deposit large crystals on the collagen surface, which are based on the classical theory of ion-mediated crystallization.[^125^](#_ENREF_125)^,^[^126^](#_ENREF_126)^,^[^129^](#_ENREF_129) However, these attempts neglect the crucial roles of NCPs during biomineralization and fail to reproduce the deposition of intrafibrillar minerals within dentin collagen fibrils.[^129^](#_ENREF_129) By contrast, biomineralization-inspired strategies based on nonclassical crystallization pathway have achieved great successes of inducing intrafibrillar mineralization of demineralized dentin.[^49^](#_ENREF_49) Many unprecedented NCPs analogs and novel remineralization methods have been developed. Therefore, in order to provide some suggestions for subsequent related studies, several key requirements for biomineralization-inspired materials for dentin remineralization are proposed here. (1) Good biocompatibility. Many of these materials are prepared *via* chemical syntheses, so their biocompatibility needs to be strictly evaluated.[^132^](#_ENREF_132) (2) Realization of both intrafibrillar and extrafibrillar mineralization. Since the mechanical properties and anti-degradability of dentin mainly depends on the degree and pattern of mineralization, remineralized dentin possessing similar structures with native dentin is highly desired.[^56^](#_ENREF_56)^,^[^133^](#_ENREF_133) (3) Fast remineralization process in oral conditions.[^134^](#_ENREF_134)

3.3. Biomineralization-inspired materials for dentin remineralization

3.3.1. 8DSS peptide: inspired by dentin phosphoprotein

Dentin phosphoprotein (DPP) accounts for more than 50% of the non-collagenous extracellular matrix proteins found in dentin, and is highly acidic due to the abundance of aspartic acid (40%) and serine/phosphoserine (50%) residues in it.[^135^](#_ENREF_135) The ability of DPP to regulate the nucleation and growth of HAP is believed to be principally due to its numerous repetitive units of Asp–Ser–Ser (DSS).[^136^](#_ENREF_136) In 2010, Yarbrough *et al.*[^136^](#_ENREF_136) firstly investigated the effects of the length of DSS peptides on the binding affinity towards defined HAP substrates and found that the affinity increased proportionally to the length, until the number of repetitive units reached up to 6 (18 amino acids); thereafter, the increase was very little, despite further increasing the number to 8. Therefore, 8DSS peptide was determined to be the most promising DPP peptide for promoting mineralization.[^129^](#_ENREF_129)

In 2015, Li *et al.*[^131^](#_ENREF_131) utilized 8DSS peptide to remineralize completely demineralized dentin. 8DSS could bind firmly with dentin collagen and induce mineral precipitation within the dentinal tubules and on the surfaces. 8DSS treatment remarkably enhanced the elastic modulus and hardness of demineralized dentin. In contrast, few crystals and no promotion of the mechanical properties were observed in the absence of 8DSS. In a subsequent study,[^137^](#_ENREF_137) 8DSS was found to effectively induce strong dentinal tubule occlusion and the dentin permeability was significantly reduced. Despite the great potential of 8DSS for restoring dentin demineralization and alleviating dentin hypersensitivity, intrafibrillar mineralization of collagen fibrils has not yet been achieved by 8DSS. To demonstrate this possibility, the stabilization effect of 8DSS on amorphous precursor of mineral may need to be studied in the future.

3.3.2. Peptides inspired by dentin matrix protein 1

Dentin matrix protein 1 (DMP1), an acidic phosphoprotein found in dentin and bone,[^138^](#_ENREF_138) plays an active role in regulating HAP nucleation and growth during dentin and bone biomineralization.[^139^](#_ENREF_139) Owing to the difficulties in extracting and purifying natural DMP1, several studies have resorted to the use of small peptides inspired by DMP1 for dentin remineralization.[^138^](#_ENREF_138)^,^[^140-142^](#_ENREF_140) In particular, the DSESSEEDR sequence is widely reserved in these peptides, according to its strong collagen binding ability. For instance, George *et al.*[^140^](#_ENREF_140) combined the collagen-binding domain derived from the C-terminus of DMP1 with two unique calcium-binding domains found in endogenous DMP1, obtaining two small peptides. The two synthetic peptides were able to bind demineralized dentin and promote remineralization of native and collagenase-challenged dentin matrices.

These studies indicate that the collagen-binding sequence derived from DMP1 can be combined with different functional peptide sequences, such as calcium-binding peptides. More possible functional peptides can be introduced in such way to expand the application range of DMP1-inspired peptides in collagen mineralization.

3.3.3. Other peptides

Amelogenin and collagen are both present at the dentin-enamel boundary (DEB), where the crystals’ growth and structural alignment are regulated by the interactions between amelogenin and collagen.[^143^](#_ENREF_143) An *in vitro* study[^143^](#_ENREF_143) has demonstrated that collagen fibrils are capable of inducing amelogenin assembly, and guiding oriented deposition of ACP particles. Based on these findings, Moradian-Oldak *et al.*[^144^](#_ENREF_144) utilized an amelogenin-inspired peptide, P26, to stimulate the interactions between collagen fibrils and amelogenin and restore demineralized dentin. P26 peptide consists of two functional domains of amelogenin, and appeared as spherical assemblies in the close vicinity of collagen fibrils (Fig. 4(I)). More importantly, P26 promoted the penetration of ACP nanoparticles into collagen fibrils. Compared with the control group, the exposed collagen fibrils within the dentinal tubules were remineralized *via* P26 treatment. In addition, the mineral density, and mechanical properties of the remineralized dentin were significantly improved by P26.

Cementum protein 1 (CEMP1) regulates the collagen mineralization of cementum, mainly through its polar residues and phosphoserine. Inspired by the polyelectrolyte nature of CEMP1, Sun *et al.*[^145^](#_ENREF_145) fabricated a novel amphiphilic oligopeptide consisting of an alkane tail and a hydrophilic peptide inspired by CEMP1. Calcium ions could trigger the self-assembly of the oligopeptide, which then induces the formation of an amorphous precursor and intrafibrillar mineralization of reconstituted collagen fibrils. In addition, a 30 mm-thick remineralized layer and occlusion of deep dentinal tubules were observed after treatment with self-assembled oligopeptide.

3.3.4. Poly(amido amine) dendrimers

Dendrimers are a class of synthetic, well-defined, and regularly branched macromolecules with mono-dispersity.[^146^](#_ENREF_146) The distinct molecular structure of dendrimers typically consists of three different domains: a central core, branches, and terminal functional groups.[^147^](#_ENREF_147) Dendrimers have been extensively studied because of their close resemblance to globular proteins.[^148^](#_ENREF_148)^,^[^149^](#_ENREF_149) Poly(amido amine) (PAMAM) is the earliest dendrimer type being synthesized, characterized, and commercialized.[^150^](#_ENREF_150)

In the early 2000s, PAMAM dendrimers have been reported to regulate the crystallization behavior of calcium carbonate and calcium phosphate.[^151^](#_ENREF_151)^,^[^152^](#_ENREF_152) Over the last decade, PAMAM dendrimers have been utilized to mimic the functions of natural NCPs to induce bioinspired mineralization.[^132^](#_ENREF_132)^,^[^153^](#_ENREF_153) Since PAMAM dendrimers can be modified at their molecular peripheries, PAMAM dendrimers with different terminal functional groups have been synthesized to promote dentin remineralization;[^154^](#_ENREF_154) these include PAMAM–COOH,[^132^](#_ENREF_132)^,^[^155^](#_ENREF_155)^,^[^156^](#_ENREF_156) PAMAM–PO_3_H_2_,[^157-159^](#_ENREF_157) PAMAM–NH_2_,[^160-164^](#_ENREF_160) and PAMAM–OH.[^165^](#_ENREF_165) For example, our group[^132^](#_ENREF_132) reported that *in situ* remineralization of human dentin and intrafibrillar mineralization within collagen fibrils were successfully achieved by the treatment of 4th generation PAMAM–COOH, which integrated sequestration and templating effects of dentin NCPs together. Furthermore, hydrophobic drugs such as apigenin and triclosan can be loaded into the inner cavity of PAMAM dendrimers to provide antibacterial activity besides mineralization.[^155^](#_ENREF_155)^,^[^158^](#_ENREF_158) Recently, nanoparticles of ACP,[^166-172^](#_ENREF_166) HAP,[^173^](#_ENREF_173) and bioactive glass[^174^](#_ENREF_174) have been combined with PAMAM dendrimers to achieve synergistic effects on dentin remineralization.[^175^](#_ENREF_175) These studies demonstrate the great potentials of PAMAM dendrimers in promoting dentin remineralization.

Despite the above-mentioned efforts, the interactions between PAMAM dendrimers and collagen fibrils, and the regulation behavior of the immobilized dendrimers on mineral nucleation and growth in solution, are still poorly understood. Especially considering that the generation and terminal functional groups of PAMAM dendrimers can be changed, more systematic studies are needed to illustrate the specific role of PAMAM dendrimers in inducing dentin remineralization.

3.3.5. Amorphous calcium phosphate precursor stabilized by polyelectrolytes

Accumulating evidence indicates that amorphous calcium phosphate (ACP) precursor is a transient phase during the formation of mineralized collagen in bone and dentin.[^43^](#_ENREF_43) Without additives, ACP is unstable and quickly turns into the more thermodynamically stable HAP. Although the exact mechanism of ACP infiltration into collagen fibrils remains largely unknown, several hypotheses based on *in vitro* models have been proposed, including electrostatic interaction,[^176^](#_ENREF_176) capillary force,[^42^](#_ENREF_42) and the balance between electroneutrality and osmotic equilibrium[^50^](#_ENREF_50) models. These hypotheses emphasize the importance of the ACP precursor in facilitating intrafibrillar mineralization.[^49^](#_ENREF_49) Owing to their highly acidic nature, NCPs capture calcium ions and prevent the premature crystallization of ACP.[^10^](#_ENREF_10) Therefore, anionic and cationic polyelectrolytes capable of stabilizing ACP have been studied as analogs of NCPs for inducing intrafibrillar mineralization.

Since many NCPs are enriched in acidic residues, particularly aspartic acid and phosphoserine,[^42^](#_ENREF_42) anionic polyelectrolytes rich in carboxylate groups are often employed as simple analogs of NCPs to stabilize ACP precursors and promote collagen mineralization. In 2007, Gower *et al.*[^42^](#_ENREF_42) reported the achievement of *in vitro* intrafibrillar mineralization of type-I collagen through the proposed polymer-induced liquid precursor (PILP) process for the first time. They utilized poly(aspartic acid) (PAsp) to stabilize ACP, and an amorphous liquid precursor was induced and absorbed into the voids inside collagen fibrils through capillary force and translated into HAP upon loss of water. Based on this pioneering study, later studies have exploited the concept of ACP precursors stabilized by anionic polyelectrolytes for remineralization of dentin.[^177-180^](#_ENREF_177) The most frequently used anionic polyelectrolytes are PAsp and poly(acrylic acid) (PAA). For instance, Marshall *et al.*[^177^](#_ENREF_177) applied the PILP system induced by PAsp on partially demineralized dentin, and recovered the mechanical properties with progressive intra- and extra-fibrillar mineralization. These studies represent an important step of the application of polyelectrolytes-stabilized ACP, from *in vitro* model of collagen mineralization towards the remineralization of truly demineralized tooth dentin.

Over the last few years, mineralization systems based on ACP precursors have been further expanded. To promote the effects of dentin remineralization, several molecules, including _L_-glutamic acid,[^181^](#_ENREF_181) aspartic acid,[^182^](#_ENREF_182) glutaraldehyde,[^183^](#_ENREF_183) citrate,[^133^](#_ENREF_133) chondroitin sulfate[^184^](#_ENREF_184) and polydopamine[^185^](#_ENREF_185) have been introduced to accelerate the crystallization kinetics or facilitate the intrafibrillar penetration of the ACP precursor. For example, Tang *et al.*[^133^](#_ENREF_133) treated collagen fibrils with citrate and enhanced the wetting effect of ACP precursor towards collagen fibrils by remarkably reducing the interfacial energy between them (Fig. 4(II): a-e). The citrate treatment efficiently promoted the remineralization of demineralized dentin, as indicated by complete remineralization within four days (Fig. 4(II): g). This study realizes the promotion effects of biological molecules on intrafibrillar mineralization through wetting management, which is an innovative step forward toward the discovery of the functions of other biomolecules. In addition to solution-based systems, anionic polyelectrolyte-stabilized ACP can be loaded in mesoporous silica[^186^](#_ENREF_186)^,^[^187^](#_ENREF_187) or self-etch adhesive,[^188^](#_ENREF_188)^,^[^189^](#_ENREF_189) fabricating carrier systems of mineral precursors that can be delivered to induce intrafibrillar mineralization.

Another strategy for intrafibrillar mineralization emphasizes dual simulations of both calcium phosphate-binding (sequestration motif: stabilizing ACP precursor) and collagen-binding (templating motif: attracting ACP precursor and initiating nucleation at specific sites) of NCPs.[^56^](#_ENREF_56)^,^[^89^](#_ENREF_89) Tay *et al.*[^56^](#_ENREF_56) reported the first dual-analogue bioinspired system consisting of poly(vinylphosphonic acid) (PVPA) and PAA for remineralization of partially demineralized dentin. PVPA was used as an analogue of phosphoproteins, which could bind specific sites of collagen and further recruit ACP precursor stabilized by PAA into the collagen fibrils. Highly ordered alignment of intrafibrillar apatite crystallites inside dentin collagen was observed after remineralization for eight weeks in the presence of both PVPA and PAA. Although PAA alone was able to achieve intrafibrillar mineralization, Tay *et al.*[^89^](#_ENREF_89) demonstrated that such mineralized collagen lacked the hierarchy of apatite assembly in natural bone and dentin. The dual-analogue system of PVPA and PAA has also been applied to remineralize demineralized resin-bonded dentin in subsequent studies, demonstrating its potential as an efficient delivery system in future clinical use.[^190-194^](#_ENREF_190) In addition to PVPA, sodium trimetaphosphate,[^195-197^](#_ENREF_195) sodium tripolyphosphate[^198^](#_ENREF_198) and phosphorylated chitosan[^199^](#_ENREF_199) have also been utilized as one of the dual analogues for templating the deposition of intrafibrillar crystals.[^57^](#_ENREF_57) Despite many reports on using dual simulations, the simultaneous requirement for two agents makes the preparation procedure complicated. Therefore, it is worth trying to develop novel molecules that combining the two functions in a single molecule.

In addition to the widely used anionic polyelectrolytes in bioinspired mineralization, poly(allylamine) hydrochloride (PAH), a cationic polyelectrolyte, is also capable of stabilizing crystallization precursor.[^200^](#_ENREF_200) For the first time, Tay *et al.*[^50^](#_ENREF_50) achieved the cationic polyelectrolyte-directed intrafibrillar mineralization of collagen using PAH-stabilized ACP (PAH-ACP) and obtained identical results for collagen mineralization *via* PAsp-stabilized ACP (PAsp-ACP). Additional cationic and anionic collagen models have been established to examine the mechanisms of intrafibrillar uptake of polyelectrolyte-stabilized ACP. The traditional hypothesis based on electrostatic interactions is inadequate for explaining polyelectrolyte-directed intrafibrillar mineralization. Thus, a new hypothesis of simultaneously balancing electroneutrality and osmotic equilibrium to follow the Gibbs–Donnan equilibrium has been proposed to supplement the existing mechanisms of intrafibrillar mineralization of collagen. As a relatively new paradigm of collagen mineralization, both PAH-ACP in solution[^49^](#_ENREF_49) or loaded in mesoporous silica[^201^](#_ENREF_201) have shown promotive effects on dentin remineralization. Moreover, further research is necessary to the discovery of other cationic polyelectrolytes on the stabilization of ACP, which may expand the application scope of cationic polyelectrolytes-stabilized ACP.

4. Enamel remineralization

As the hardest tissue in the human body, enamel performs important functions of food mastication in daily life. However, mature enamel is unable to self-regenerate when subjected to substantial mineral loss, such as dental caries and erosion. Despite commercially available products and conventional methods for enamel loss treatment, regenerating the natural structure of enamel remains challenging. By learning from the pathways and mechanisms of enamel biomineralization, some bioinspired materials have achieved outstanding effects on remineralization and structure regeneration of enamel. In this section, we firstly provide an introduction to the biomineralization of enamel. Then, several key requirements for biomineralization-inspired materials for enamel remineralization are discussed. Finally, we focus on the advances of biomineralization-inspired materials for enamel remineralization, especially recombinant amelogenin, protein-inspired synthetic peptides, poly(amido amine) dendrimers, and inorganic materials. The biological performances of some representative materials are listed in Table 3.

4.1. Biomineralization of enamel

Biomineralization of tooth enamel or amelogenesis is a biologically programmed sequence of several consecutive stages, including two major functional stages: secretory and maturation.[^202-204^](#_ENREF_202) During the secretory stage, ameloblasts are mainly responsible for producing enamel matrix proteins and proteinases, which control the shape and arrangement of initially formed enamel crystals.[^19^](#_ENREF_19) At the maturation stage, the thickness and width of enamel crystals greatly expand, accompanied by a decrease in protein abundance, and eventually form a highly mineralized tissue consisting of approximately 96 wt.% of mineral and a small amount of residual biomacromolecules and water.[^205^](#_ENREF_205) Herein, we introduce two key issues involved in the biomineralization of enamel, which are important to the structure formation and degree of mineralization of enamel.

4.1.1. Amelogenin and enamel formation

The excellent mechanical properties of enamel mainly arise from its exquisite hierarchical structures.[^206^](#_ENREF_206) Among them, the most attractive structure is the organized crystals of enamel rods.[^207^](#_ENREF_207)^,^[^208^](#_ENREF_208) The formation of enamel rods with strict shapes is closely related to enamel matrix proteins, especially amelogenin.[^19^](#_ENREF_19) Amelogenin is the most important and abundant enamel matrix protein.[^5^](#_ENREF_5)^,^[^19^](#_ENREF_19) During enamel formation, amelogenin is highly processed by enzymes upon its secretion by ameloblasts,[^209^](#_ENREF_209) and its proteolytic products regulate apatite mineralization.[^8^](#_ENREF_8)^,^[^210-212^](#_ENREF_210) They form nanoribbons through self-assembly, and these nanoribbon scaffolds template the oriented growth of apatite fibers to form enamel rods.[^23^](#_ENREF_23)^,^[^25^](#_ENREF_25)^,^[^213-215^](#_ENREF_213)

4.1.2. Proteins removal and enamel maturation

At the maturation stage of enamel, the cleavage and degradation of the proteinaceous matrix *via* proteolytic processes are crucial for the formation of enamel with a high degree of mineralization.[^202^](#_ENREF_202) A study by Moradian-Oldak *et al.*[^216^](#_ENREF_216) demonstrated that the occlusion of undegraded amelogenin inside enamel crystals of mice occurred due to the lack of matrix metalloprotease-20 (MMP-20) during amelogenesis. Due to amelogenin occlusion, the enamel crystals in MMP-20 null mice had uncommon sizes, morphologies, and crystallinities.

4.2. Requirements for biomineralization-inspired materials for enamel remineralization

Conventional substitutional materials of enamel include amalgams, resins and ceramics, which are significantly distinct from native enamel in terms of chemical composition and crystal microstructures.[^8^](#_ENREF_8)^,^[^217-219^](#_ENREF_217) Therefore, the performance and appearance of these materials are not as good as those of natural enamel. Besides, secondary caries usually arise due to the unsatisfactory fitting and poor adhesion to enamel of these filling materials.[^8^](#_ENREF_8)^,^[^220^](#_ENREF_220) Furthermore, weakly bonded interface may lead to the shedding of restorative materials, which means the failure of restoration and requires a second surgery. *In situ* regeneration of HAP on the etched enamel surface is a promising method due to the formation of enamel-like crystals.[^221^](#_ENREF_221) Hitherto, several methods have been applied for the regeneration of enamel, such as electrolytic deposition, hydrothermal synthesis, and the use of surfactants and hydrogen peroxide.[^222-228^](#_ENREF_222) Unfortunately, these methods are implemented under stringent conditions, which are not suitable for clinical applications.[^217^](#_ENREF_217) Despite extensive use in clinics, excessive intake of fluoride causes dental fluorosis during enamel development,[^229^](#_ENREF_229) and enhancement of fluoride resistance in microorganisms.[^230^](#_ENREF_230)

Current restoration materials and technologies are still far away from the regeneration of natural enamel. To minimize this gap, researchers resort to the biomineralization of enamel for inspiration of the design and construction of materials for enamel remineralization.[^3^](#_ENREF_3)^,^[^6^](#_ENREF_6)^,^[^8^](#_ENREF_8) Despite numerous reports on biomineralization-inspired materials, some important challenges remain to be solved. Herein, we propose several key requirements for these materials. (1) Good biocompatibility. Since these materials contact with the oral cavity and may enter the digestive system, amounts of toxic substances involved in these materials should be controlled at a safe level.[^231^](#_ENREF_231)^,^[^232^](#_ENREF_232) (2) The newly regenerated enamel layer should be tightly bonded with the original enamel without obvious boundaries, which avoids the fracture at the repaired interfaces and repeated restoration processes.[^227^](#_ENREF_227)^,^[^233^](#_ENREF_233) (3) The newly regenerated enamel layer should have similar structures with native enamel to ensure the mechanical properties of the repaired enamel.[^234^](#_ENREF_234)^,^[^235^](#_ENREF_235) (4) The remineralization process should be completed in a short time in physiological environments, avoiding harsh operating conditions and excessive application time.[^236^](#_ENREF_236)

4.3. Biomineralization-inspired materials for enamel remineralization

4.3.1. Amelogenin and amelogenin-inspired peptides

Amelogenin and its cleavage products account for > 90% of the extracellular organic matrix of forming enamel.[^237^](#_ENREF_237) Generally, amelogenin can be divided into three major domains: a hydrophobic tyrosine-rich N-terminus, a hydrophobic central proline-rich domain, and a hydrophilic and negatively charged C-terminus.[^5^](#_ENREF_5)^,^[^238^](#_ENREF_238)^,^[^239^](#_ENREF_239) Due to the sequence and nature of its compositional amino acids, amelogenin is intrinsically disordered[^19^](#_ENREF_19)^,^[^240^](#_ENREF_240)^,^[^241^](#_ENREF_241) and hydrophobic on the whole.[^237^](#_ENREF_237)^,^[^242^](#_ENREF_242) Therefore, amelogenin tends to construct an environment-fitted structure.[^8^](#_ENREF_8) Through intermolecular hydrophobic interactions, amelogenin self-assembles into assemblies with various quaternary structures *in vitro*, including oligomers, nanospheres, nanochains, nanoribbons, and microribbons, under different conditions (Fig. 5(I)).[^19^](#_ENREF_19)^,^[^213^](#_ENREF_213)^,^[^214^](#_ENREF_214)^,^[^243-245^](#_ENREF_243) More importantly, these supramolecular nanospheres of amelogenin are similar to the self-assembled periodic structures observed in naturally formed enamel,[^246^](#_ENREF_246)^,^[^247^](#_ENREF_247) which are believed to be fundamental block units of the developing enamel extracellular matrix[^248^](#_ENREF_248) that play important roles in guiding apatite growth, along with their further assembled structures.[^3^](#_ENREF_3)^,^[^5^](#_ENREF_5)^,^[^213^](#_ENREF_213)^,^[^214^](#_ENREF_214)^,^[^249^](#_ENREF_249) Indeed, several mineralization-related traits of amelogenin have been confirmed by *in vitro* studies, including the abilities to stabilize amorphous calcium phosphate (ACP), regulate crystal polymorphism, and promote parallel crystal organization.[^212^](#_ENREF_212)^,^[^214^](#_ENREF_214)^,^[^250-254^](#_ENREF_250)

Since amelogenin is of pivotal importance to apatite crystal formation during enamel development, its ability to regulate apatite mineralization has been harnessed to fabricate biomaterials with enamel-like nano- and micro- structures in many *in vitro* studies.[^8^](#_ENREF_8)^,^[^215^](#_ENREF_215)^,^[^221^](#_ENREF_221) One of the most facile strategies is to introduce recombinant amelogenin into mineralization solutions, amid which the recombinant amelogenin is able to modulate the growth of apatite crystals on certain substrates.[^255-258^](#_ENREF_255) For example, Moradian-Oldak *et al.*[^256^](#_ENREF_256) synthesized an enamel-mimicking nanocomposite coating consisting of full-length recombinant porcine amelogenin (rP172) and calcium phosphate on the surface of silicon wafers. It was observed that rP172 could self-assemble into nanochains with an increase in solution pH, resulting in the arrangement of parallel bundles of calcium phosphate crystal. It was suggested that the formation of parallel rod-like crystal bundles was attributed to the supramolecular self-assembly of rP172 nanospheres into nanochains.

Another strategy to rebuild enamel-like structures is to fabricate amelogenin-containing hydrogels,[^259-263^](#_ENREF_259) which are able to regulate crystal growth by releasing amelogenin. A typical example[^263^](#_ENREF_263) is amelogenin-containing chitosan (CS-AMEL) hydrogel. CS-AMEL hydrogel was used to cover etched enamel, and it was suggested that the loaded amelogenin was capable of stabilizing CaP clusters and organize them into chains through its supramolecular assembly, eventually leading to enamel-like co-aligned crystals fused with original enamel crystals. After mineralization with CS-AMEL hydrogel for seven days, a tightly bound homogeneous new layer was observed on the etched enamel surface (Fig. 5(II)). The new layer consisted of many parallel nanocrystals aligned preferentially along the longitudinal direction. Moreover, the needle-like crystals of the new layer were bundled together into a basic organizational unit resembling the crystallites of natural enamel. In addition, the CS-AMEL hydrogel efficiently inhibited bacterial growth due to the presence of chitosan. However, the newly grown layer had inferior mechanical properties than those of healthy enamel, which was partly due to the occlusion of undegraded organic materials inside the formed crystals.[^8^](#_ENREF_8) Hence, in order to fabricate biomaterials with composition and properties that closely resemble the natural enamel, crystal growth modulated by amelogenin can be coupled with the proteolytic cleavage of amelogenin itself.[^264^](#_ENREF_264) For this purpose, MMP-20 was added into CS-AMEL hydrogel to prevent amelogenin occlusion in forming crystals during remineralization of etched enamel surface.[^265^](#_ENREF_265) Amelogenin could be degraded by MMP-20, and MMP-20-containing hydrogel-treated sample exhibited a higher degree of orientation and crystallinity than the samples treated without MMP-20. More importantly, the mechanical properties of the induced HAP layer increased remarkably by introducing MMP-20. This study provides a novel idea for improving the mechanical properties of repaired enamel by removing the organic substances involved during repairing process.

The unique ability of full-length amelogenin to self-assemble, control crystal spacing, and bind to the crystal surface, as well as its solubility are believed to stem from several separate functional domains, including the hydrophobic N-terminus, the central polyproline repeating domain, and the hydrophilic C-terminus.[^221^](#_ENREF_221)^,^[^266^](#_ENREF_266) This means that a single peptide fragment of amelogenin may have the potential to realize partial properties and functions of full-length amelogenin. Based on this concept, peptides inspired by amelogenin have been used as substitutes for native amelogenin to regulate enamel remineralization *in vitro*. In practical operations, peptides offer several advantages over amelogenin, such as reduced cost, enhanced design flexibility, and easier penetration into the enamel interior.[^233^](#_ENREF_233)^,^[^267^](#_ENREF_267)

Leucine-rich amelogenin peptide (LRAP) is the most abundant alternative splicing product of amelogenin during enamel development.[^202^](#_ENREF_202)^,^[^268^](#_ENREF_268) LRAP conserves the N-terminal and C-terminal sequences of native amelogenin,[^221^](#_ENREF_221)^,^[^269^](#_ENREF_269) which are crucial to the enamel growth regulating effects of LRAP that have been confirmed in mouse models.[^270^](#_ENREF_270) A study by Norcy *et al.*[^268^](#_ENREF_268) demonstrated that LRAP succeeded in self-assembly into nanospheres and regulated mineralization of calcium phosphate, indicating significant similarities between LRAP and full-length amelogenin. In addition, the C-terminus and N-terminus of amelogenin in non-phosphorylated LRAP were capable of inducing the transformation of ACP to aligned bundles of needle-like crystals. Several bioinspired approaches for enamel remineralization have been conducted by implementing LRAP-mediated mineralization.[^267^](#_ENREF_267)^,^[^269^](#_ENREF_269)^,^[^271^](#_ENREF_271)^,^[^272^](#_ENREF_272) For example, Kwak *et al.*[^271^](#_ENREF_271) achieved the remineralization of acid-etched enamel using LRAP in the presence of pyrophosphate. Moradian-Oldak *et al.*[^267^](#_ENREF_267) evaluated the enamel repair efficiency of a CS-LRAP hydrogel and compared it with the previously reported CS-AMEL hydrogel. The demineralized enamel treated with CS-LRAP hydrogel showed much faster rates of crystal nucleation and growth than the full-length counterpart, probably due to the higher hydrophilicity of LRAP.

Another strategy to design and synthesize bioinspired alternatives of amelogenin is to construct appropriate functional peptides by comparing, selecting and mimicking the specific amino acid sequences of amelogenin. Sarikaya *et al.*[^273^](#_ENREF_273) designed a 15-amino acid (15-AA) peptide named shorten human amelogenin-derived peptide 5 (shADP5), which was generated using a bioinformatic scoring matrix. Both 7-AA and 12-AA long segments of full-length amelogenin shared high similarity scores with experimentally selected 7-AA and 12-AA long HAP binding peptides. Then, the overlapping regions between the high-similarity segments were determined as putative strong binding regions, and subsequently sifted and refined to design shADP5. shADP5 resulted in the formation of a considerably thick (10 μm) dense layer of HAP crystals on demineralized enamel after mineralization for 1 h.

Using this strategy, novel peptides consisting of recombined peptide domains of amelogenin can be achieved. For instance, Moradian-Oldak *et al.*[^233^](#_ENREF_233) synthesized 26-residue and 32-residue peptides, namely P26 and P32, respectively. P26 was composed of the last twelve residues of C-terminus and the inner fourteen residues of N-terminus, while P32 had two more polyproline repeating regions (PVH/PMQ) from the intermediate hydrophobic core of native amelogenin. P26 and P32 self-assembled into nanospheres and regulate apatite nucleation *in* *vitro*. In addition to the improved preferential orientation towards regenerated HAP crystals, P26 and P32 were capable of inducing multilayered aprismatic crystals after repeated treatment, forming seamless interfaces with the underlying native enamel.

QP5, a 22-residue peptide composed of five tandem glutamine-proline-X (Gln-Pro-X) repeating units followed by a 7-residue hydrophilic segment, is another amelogenin-inspired peptide that was first reported by Lv *et al*.[^274^](#_ENREF_274) The construction of QP5 was based on the highly repetitive Gln-Pro-X sequence identified in native amelogenin. QP5 exhibited promotive effects on enamel caries remineralization. QP5 has been widely developed and exhibits huge application potential for enamel caries treatment.[^275-280^](#_ENREF_275)

4.3.2. Peptides inspired by salivary acquired pellicle

Salivary acquired pellicle (SAP) is a permanently coated thin layer on the surface of oral tissues, which consists of adsorbed proteins and glycoproteins.[^281^](#_ENREF_281) Among the many proteins contained in SAP, phosphoproteins such as statherin, histatin, and proline-rich proteins exhibit a high affinity towards HAP and are among the first proteins absorbed onto the HAP surface from glandular saliva.[^282^](#_ENREF_282)^,^[^283^](#_ENREF_283) Statherin is a calcium-binding protein composed of 43 amino acid residues rich in acidic proline and tyrosine, and it is able to prevent spontaneous precipitation in a saturated solution of calcium phosphate.[^284^](#_ENREF_284)^,^[^285^](#_ENREF_285) The highly charged N-terminal fragment (the first 15 residues with a sequence of DpSpSEEKFLRRIGRFG, SN15) of statherin exhibited stronger adsorption affinity toward HAP than whole statherin and its other fragments; in addition, the replacement of phosphoserines (pS) with aspartic acid (D) residues had no effect on its binding affinity and crystal growth inhibition properties.[^286^](#_ENREF_286)

Inspired by this, several small peptides have been designed and applied, including DDDDEEKFLRRIGRFG (SN_A_15),[^287^](#_ENREF_287)^,^[^288^](#_ENREF_288) DpSpSEEKC,[^289^](#_ENREF_289) DDDEEK,[^290^](#_ENREF_290)^,^[^291^](#_ENREF_291) and DDDEEKC.[^292-297^](#_ENREF_292) Although two phosphorylated serine residues (pSpS) are replaced, it should be noted that the latter two simpler peptides also possess strong binding affinity towards HAP because of the conservation of the first six highly charged residues. For example, our group[^293^](#_ENREF_293) synthesized a peptide-7, DDDEEKC, which was intended to simulate the six binding amino acids of the N-terminus of statherin. Peptide-7 had a strong affinity towards HAP, as confirmed by the adsorption experiments showing that most of peptide-7 remained on the HAP surface after seven days of desorption. Owing to this strong binding affinity, peptide-7 induced the formation of a compact crystal layer when applied to acid-etched enamel. More importantly, the regenerated crystals induced by peptide-7 had similar properties to those obtained by fluoride treatment. *In vivo* investigations in rats demonstrated that the remineralization capability of peptide-7 was comparable to that of fluoride, indicating that peptide-7 may be utilized as a non-fluoride mineralizing agent. In another study, the cysteine of peptide-7 was utilized as a linkage to connect oligomeric procyanidins, which have bacteriostatic effects.[^297^](#_ENREF_297) Therefore, the whole material was able to simultaneously resist bacteria and induce enamel remineralization. Based on the strong binding affinity of SAP-inspired peptides towards HAP, future studies may focus on combining this HAP-binding capability with other functional properties, such as anti-fouling property, to expand the application of SAP-inspired peptides.

4.3.3. Self-assembling peptides

Biomineralization is generally believed to be related to the acidic proteins in the *β*-sheet conformation. Based on this concept, some peptide assemblies with predefined secondary structures have been utilized as crystallization templates for several inorganic minerals.[^298^](#_ENREF_298) Furthermore, researchers have exploited bioinspired peptides, capable of self-assembling into scaffold-like structures mimicking the abilities of extracellular matrix proteins to regulate HAP nucleation and growth.[^299^](#_ENREF_299)

P_11_-4 is an extensively studied self-assembling small peptide. P_11_-4 in solution presents a pH-responsive self-assembly behavior, mainly through intermolecular hydrogen bonds generated from the peptide backbone.[^300^](#_ENREF_300)^,^[^301^](#_ENREF_301) Kirkham *et al.*[^302^](#_ENREF_302) treated caries-like lesions with P_11_-4 solution, and the treated samples were subjected to pH-cycling experiments, during which the exposure to acid rapidly drove the self-assembly of P_11_-4. After five days of remineralization, it was observed that P_11_-4 treatment remarkably increased the net mineral deposition on previous lesions because of the dual functions of P_11_-4 in promoting remineralization and inhibiting demineralization. The mechanism by which self-assembled P_11_-4 had promotive effects on mineralization of carious enamel was studied.[^301^](#_ENREF_301) P_11_-4 penetrated the subsurface lesion and then self-assembled into aggregates inside the whole lesion, where the assembled P_11_-4 acted as a scaffold to enhance HAP nucleation *de novo*. Due to its proven efficiency in reversing incipient occlusal and proximal carious lesions, P_11_-4 has become commercially available in the form of toothpaste.[^129^](#_ENREF_129)^,^[^303^](#_ENREF_303)^,^[^304^](#_ENREF_304)

Another type of self-assembling peptide is peptide amphiphiles, which are typically constructed by incorporating a hydrophobic alkyl chain with a peptide. Peptide amphiphiles combine the bioactive functions of peptides with the physiochemical traits of surfactants.[^305^](#_ENREF_305) The assembly of peptide amphiphiles is a combination of several major energy contributions, which may include hydrophobic interactions, hydrogen bonds and electrostatic interactions. The self-assembly mechanisms of peptide amphiphiles have been described in a review by Cui *et al*.[^305^](#_ENREF_305) Although self-assembled peptide amphiphile nanofibers have been demonstrated to be capable of directing mineralization of HAP,[^306^](#_ENREF_306) there are few reports on enamel remineralization utilizing self-assembling peptide amphiphiles. Chu *et al.* group[^307^](#_ENREF_307) designed and synthesized an anionic self-assembling peptide amphiphile composed of a stearic acid-derivative and a hydrophilic peptide tail derived from the C-terminal amelogenin. The peptide amphiphile successfully self-assembled into nanofibers with a well-defined helical twist. ACP nanoparticles were found to be located along the self-assembled nanofibers when alternatively immersed in solutions of calcium ions and phosphate ions. When applied for enamel remineralization, the presence of the peptide amphiphile led to higher packing density of the newly formed crystal layer.

4.3.4. Other proteins and peptides

Although dentin phosphoprotein (DPP) is not present in tooth enamel, its ability to affect the nucleation and growth of HAP can be utilized for enamel remineralization.[^136^](#_ENREF_136) The numerous aspartic acid–serine–serine (DSS) repeating motifs present in DPP are believed to have a remarkably strong binding affinity towards calcium ions and HAP.[^136^](#_ENREF_136)^,^[^308^](#_ENREF_308)^,^[^309^](#_ENREF_309) Inspired by this, peptides consisting of multiple repetitive DSS segments have been designed to inherit the functions of full-length DPP, and some of them have been applied for enamel remineralization.[^310-315^](#_ENREF_310) Hsu *et al.*[^311^](#_ENREF_311) utilized 8DSS to promote mineral deposition for the remineralization of acid-etched enamel for the first time. The surface roughness, hardness, and elastic modulus of demineralized enamel were all significantly improved compared with those of the samples without 8DSS treatment. Two possible and simultaneous roles during the remineralization process played by 8DSS has been proposed: (1) the binding of 8DSS to demineralized enamel prevents the loss of calcium and phosphate ions; (2) 8DSS recruits free calcium and phosphate ions from simulated body fluid proactively, facilitating mineral deposition on the enamel surface. Recently, the effects of 8DSS peptide on the remineralization of enamel caries have been evaluated in a rat model,[^313^](#_ENREF_313) and the results indicated that 8DSS rescued enamel demineralization and promoted enamel remineralization. This *in vivo* study on 8DSS peptide has laid the foundation for its clinical applications in the future. Another peptide, triplet repeats of asparagine–serine–serine (3NSS), which is very similar to DSS peptides, is obtained by replacing aspartic acid with asparagine.[^316^](#_ENREF_316)^,^[^317^](#_ENREF_317) NSS peptides are considered to be derivatives of DSS peptides because there is only a minor difference between the functional groups of asparagine (–CONH_2_) and aspartic acid (–COOH). Chung *et al.*[^316^](#_ENREF_316) reported that 3NSS was able to promote the remineralization of acid-etched enamel, which had the same working mechanisms with 8DSS. These works suggest that short peptides with repetitive sequences have significant promotive effects on mineralization, which is due to the repetitive and consecutive arrangements of functional residues, such as aspartic acid and serine. In this way, the development and application of other similar short peptides with repetitive sequences can be expected.

Yang *et al.*[^318^](#_ENREF_318) reported that lysozyme was able to undergo a fast transition into *β*-sheet-rich amyloid-like aggregates through the reduction induced by tris(2-carboxyethyl)phosphine (TCEP). During the reduction process, the *α*-helix structures in lysozyme were unlocked and the unfolded lysozyme monomers aggregated into oligomeric nanoparticles through *β*-sheet stacking, and eventually phase-transited lysozyme (PTL) was established *via* oligomer aggregates. PTL has been developed as a robust, multifunctional, biocompatible, and colorless transparent coating.[^319^](#_ENREF_319)^,^[^320^](#_ENREF_320) Recently, based on a previous study, Yang *et al.* recognized a similarity between PTL and N-terminal amelogenin,[^236^](#_ENREF_236) which is believed to be indispensable for the self-assembly of amelogenin into amyloid-like aggregation *via* *β*-sheet stacking.[^321^](#_ENREF_321)^,^[^322^](#_ENREF_322) Furthermore, a synthesized peptide, C-AMG, derived from amelogenin C-terminus, was introduced to control HAP orientation. Thus, PTL/C-AMG, as a single amelogenin-inspired matrix, was expected to fulfill the functions of both the N-terminus and C-terminus of amelogenin in facilitating the formation of enamel-like crystals (Fig. 6a). PTL/C-AMG was capable of inducing regularly arranged enamel-like crystals with identical orientations, successfully realizing the exact texture of native enamel. In addition, the newly grown layer formed by PTL/C-AMG showed excellent mechanical properties. *In vivo* remineralization experiments in the oral cavity of rats demonstrated that PTL/C-AMG had a better effect in regenerating enamel-like crystals than that of fluoride (Fig. 6: b-e). This study suggests the significance of amyloid-like protein in inducing mineralization and proves a simple and potential approach for treating dental caries.

Tuftelin is an acidic non-amelogenin protein found in tooth enamel, which is believed to be involved in dental enamel mineralization.[^323^](#_ENREF_323) A common domain that provides binding sites for calcium ions has been identified in the tuftelin proteins of human and several other mammals.[^324^](#_ENREF_324) Inspired by this, Ding *et al.*[^325^](#_ENREF_325) synthesized a tuftelin-derived peptide (TDP), which simulated the calcium binding domain of tuftelin. TDP was demonstrated to be capable of attracting calcium and phosphate ions, and significantly promoting the remineralization of initial carious lesions. Despite the initiative work of using tuftelin-inspired peptide for enamel remineralization, the mechanism of promotion of mineralization and the role of calcium-binding domain are not fully understood, and further studies are required about these issues.

4.3.5. Poly(amido amine) dendrimers

Studies have revealed that PAMAM dendrimers can control the shape and size of HAP *via* different surface groups, generations and concentrations.[^326-330^](#_ENREF_326) Subsequently, the self-assembly of PAMAM dendrimers or dendrons was observed to be similar to that of amelogenin.[^148^](#_ENREF_148) In our previous work,[^149^](#_ENREF_149) a carboxyl-terminated PAMAM was found to be prone to hierarchically self-assemble into nanospheres, nanochains, microfibers, and macroscopic aggregates. In a subsequent study by our group,[^331^](#_ENREF_331) phosphorylated dendronized PAMAMs also demonstrated its self-assembly into ribbons or fibrils and induction of the deposition of HAP. These studies offer a new paradigm for simulating natural amelogenin from its structures and self-assembly behaviors by using synthetic dendrimers.

Several studies have focused on the regeneration of enamel facilitated by PAMAM-type dendrimers,[^218^](#_ENREF_218)^,^[^231^](#_ENREF_231)^,^[^332-335^](#_ENREF_332) whose intrinsic similarities with amelogenin are supposed to be the theoretical foundation for mimicking the functions of amelogenin.[^153^](#_ENREF_153) For instance, our group[^218^](#_ENREF_218) synthesized a carboxyl-terminated PAMAM–alendronate conjugate (ALN–PAMAM–COOH) to specifically bind on HAP through the strong interaction between ALN and the HAP surface. ALN–PAMAM–COOH could absorb tightly on the enamel and facilitate nanorod-like crystals formation with high uniformity on acid-etched enamel. The structure of the newly grown crystals was analogous to native enamel; besides, the microhardness was mostly recovered after mineralization. In addition, *in vivo* experiments also indicated the excellent effects of ALN–PAMAM–COOH on inducing enamel remineralization in the oral cavity of rats. In a subsequent study,[^231^](#_ENREF_231) a phosphate-terminated dendrimer (PAMAM–PO_3_H_2_) was obtained by modifying a fourth-generation PAMAM with dimethyl phosphate. After mineralization, a fresh layer with a thickness of 11.23 μm was observed on acid-etched enamel treated by PAMAM–PO_3_H_2_, in contrast, the thickness of PAMAM–COOH treated sample was only 6.02 μm. These studies suggest the great potentials of PAMAM dendrimers in restoring acid-etched enamel, which can be furtherly expanded *via* introducing novel chemical modifications to the terminal functional groups in future researches.

4.3.6. Calcium phosphate-based systems

Calcium ions and phosphate ions are the fundamental units of enamel crystals. Various forms of calcium phosphate, including ACP, HAP, and calcium phosphate ion clusters, have been designed and incorporated into different systems for bioinspired regeneration of enamel.

ACP is believed to be an indispensable transient metastable precursor in biomineralization.[^211^](#_ENREF_211) During enamel biomineralization, acidic proteins can serve as nucleators or inhibitors to control the formation, stabilization and transformation of ACP.[^336^](#_ENREF_336) Based on this, Zhang *et al.*[^336-338^](#_ENREF_336) used acidic macromolecules to stabilize ACP, fabricating bioinspired composite systems dedicated to enamel remineralization. For instance, phosphorylated chitosan (Pchi) was synthesized and utilized as an analogue of phosphorylated proteins to stabilize ACP, leading to the formation of nanocomplexes of Pchi-ACP. The obtained Pchi-ACP showed a significant promotive effect on the remineralization of enamel subsurface lesions.[^336^](#_ENREF_336) However, the introduction of acidic macromolecules into repaired enamel may be detrimental to its mechanical strength, since natural enamel is a highly mineralized tissue with only tiny amounts of organic substances. Alternative strategies can focus on utilizing degradable macromolecules to stabilize ACP.

Because of its chemical similarities with native enamel, synthetic HAP is a candidate for the repair of demineralized enamel.[^228^](#_ENREF_228)^,^[^339^](#_ENREF_339) However, the actual structure of native enamel is too complex to be replicated by synthetic HAP, which differs from enamel crystal in dimensions, morphologies, and orientations, leading to its unsatisfactory effects as a repairing agent of enamel. Inspired by the nanosized building blocks of enamel, Tang *et al.*[^340^](#_ENREF_340) prepared 20 nm HAP as an analogue of the basic building blocks of enamel. The structure of the acid-etched enamel was reinforced by the 20 nm HAP and the hardness remained almost unchanged. More importantly, these promotive effects could not be observed when applying conventional 100 nm HAP or ~20 nm ACP. In another bioinspired approach, Tang *et al.*[^341^](#_ENREF_341) combined 20 nm HAP with glutamic acid to regenerate enamel-like structures under physiological conditions. Glutamic acid molecules were selectively absorbed onto (001) faces, resulting in oriented growth of the repaired layer. However, the size of HAP nanoparticles is considerably larger than that of calcium and phosphate ions, which may lead to apparent interfaces between the newly formed crystal layer and the etched enamel surface, thus deteriorating the mechanical strength of the repaired enamel.

Despite the achievements of ACP in bioinspired mineralization of tooth enamel, two major deficiencies still exist when attempting to thoroughly replicate the structure and mechanical strength of native enamel. First, a foreign ACP phase is unable to initiate epitaxial growth of enamel due to the oversized ACP nanoparticles;[^340^](#_ENREF_340) Second, some irremovable polymeric additives applied to stabilize ACP weaken the mechanical strength of the repaired enamel. Evidence has shown some biological processes of continuous epitaxial construction, during which the earlier-formed crystalline phase is covered by amorphous phase, resulting in an integrated crystalline-amorphous interface at the growth frontier.[^39^](#_ENREF_39)^,^[^342-344^](#_ENREF_342) Inspired by the crystalline-amorphous frontier in native biomineralization, Tang *et al.*[^234^](#_ENREF_234) designed a novel material based on calcium phosphate ion clusters (CPICs) utilized to fabricate an amorphous layer for the induction of epitaxial growth of enamel apatite crystals. They innovatively utilized a small molecule, triethylamine (TEA) to stabilize CPICs in ethanol[^345^](#_ENREF_345), forming ultra-small clusters (Fig. 7a) that could remain stable for two days without aggregation or size change. After treating the HAP substrate with CPIC ethanol solution, a mimetic biomineralization frontier composed of bulk ACP could be fabricated with ethanol evaporation, during this process, TEA was removed and the formation of ACP was initiated. Subsequently, the coated continuous ACP layer transformed to HAP crystals in the way of epitaxial growth due to the integration of the two phases (Fig. 7b). In contrast, the lack of a structurally continuous interface and epitaxial growth was observed when using ACP nanoparticles. The CPIC material exhibited remarkable effects on repairing acid-etched enamel, as indicated by the very similar morphological texture and mechanical strength between the repaired layer and native enamel (Fig. 7c-e). More importantly, compared with other methods of remineralization in solutions, the CPIC significantly shortens the time required for enamel repair because it contains a large amount of calcium and phosphate sources. This bioinspired tactic of creating a mineralization frontier for epitaxial growth provides a new paradigm for the regeneration of hard tissues.

4.3.7. Amorphous zirconium dioxide ceramics

After enamel biomineralization, part of the amorphous phase is maintained as intergranular phase, rendering enamel with superior mechanical properties and acid-resistance.[^207^](#_ENREF_207)^,^[^227^](#_ENREF_227)^,^[^346^](#_ENREF_346) Inspired by the unique structure of the remaining amorphous phase, which is characterized by the absence of grain boundaries and dislocations, and isotropy, Wei *et al.*[^227^](#_ENREF_227) developed a method to grow amorphous zirconium dioxide (ZrO_2_) ceramics *in situ* for enamel repair. Through sequential nucleation, deposition and growth, a homogeneous layer of ZrO_2_ was fabricated on the acid-etched enamel. The repaired enamel showed comparable mechanical properties to native enamel. In addition, owing to its high hydrophilicity, the amorphous ZrO_2_ layer exhibited excellent antibacterial adhesion and proliferation capability. However, it should be pointed out that this *in situ* method of growth of amorphous ZrO_2_ requires a high temperature treatment process (80℃, 12 h), which largely limits its clinical application. Nevertheless, this study demonstrates the possibility of restoring enamel by amorphous ceramics, which may allow the amorphous–crystalline interface design more applications.

5. Conclusions and perspectives

The hard tissues of vertebrates are biologically generated through biomineralization. Studying the underlying pathways and mechanisms of mineral formation during biomineralization will contribute to the development of novel strategies and materials for hard tissue repair. Researchers are inspired by the identified phenomena and mechanisms of biomineralization, such as the regulatory effects of biomacromolecules on minerals’ nucleation and growth. A plethora of bioinspired materials have been artificially engineered to simulate biomineralization processes for hard tissue repair. We reviewed recent studies on biomineralization-inspired ideas, as well as characteristics and repair effects of these materials.

These advances have shown significant advantages by choosing a bioinspired route. Many of these approaches or materials utilize synthetic analogs to replace natural biomacromolecules, which remarkably reduces the cost and increases the designability. In addition, the achievements of hard tissue-like materials or the promotion of *in situ* regeneration *via* bioprocess-inspired strategies are green and feasible, avoiding the complex procedures and harsh conditions required when directly mimicking the intricate structures or properties of natural hard tissues. More importantly, the development of novel materials and *in vitro* models may supplement the current mechanisms of biomineralization.

It is worth mentioning that some of these bioinspired materials have been commercialized and approved for clinical application in hard tissue repair. For instance, mineralized collagen with a bone-like nanostructure has been utilized in the treatment of bone defects caused by traumatic injury, tumors, surgical wounds *etc*.[^106^](#_ENREF_106)^,^[^107^](#_ENREF_107)^,^[^347^](#_ENREF_347) Mineralized collagen exhibits several functional advantages over traditional synthetic materials, including better biomechanics, osteoconductive and osteoinductive properties, and biodegradation. More importantly, the clinical efficacy of mineralized collagen mixed with bone marrow closely resembles autologous bone, which is currently the gold standard treatment for large bone defects.[^116^](#_ENREF_116)

Despite considerable advances of bioinspired materials for hard tissue repair, most of these efforts are at the proof-of-concept state.[^7^](#_ENREF_7)^,^[^125^](#_ENREF_125) Several unmet needs that may be obstacles for further scientific progress and clinical translation are listed here. (1) The existing bioinspired materials are limited to simulating only the nanostructure, which lacks the regulation of hierarchical structures at larger scales.[^8^](#_ENREF_8)^,^[^32^](#_ENREF_32)^,^[^57^](#_ENREF_57) (2) The evaluation of key biological properties (*e.g.*, cytotoxicity and biodegradability) of some bioinspired materials and their repair effects (*e.g.*, the mechanical properties of newly formed tissues) is incomplete.[^3^](#_ENREF_3)^,^[^10^](#_ENREF_10)^,^[^129^](#_ENREF_129) (3) The repaired tissues are not as good as their natural counterparts (*e.g.*, the inferior binding force between new layer and original surface), and the repair efficiency is low (*e.g.*, long times required for enamel and dentin remineralization).[^8^](#_ENREF_8)^,^[^19^](#_ENREF_19)^,^[^126^](#_ENREF_126) (4) The manner and environment in which some bioinspired materials are utilized are not suitable for clinical practice (*e.g.*, the liquid environment required for enamel and dentin remineralization in many experiments).[^126^](#_ENREF_126)^,^[^129^](#_ENREF_129)

Several perspectives on future work in this field are suggested in the following points. (1) Since numerous fundamental issues regarding biomineralization are still poorly understood,[^9^](#_ENREF_9)^,^[^17^](#_ENREF_17) the outcomes of the basic principles of biomineralization will be of significant importance in conceiving new tactics and materials for hard tissue repair. (2) Structural construction of mineralized tissues goes through cascaded processes with the involvement of various biomolecules and inorganic ions.[^3^](#_ENREF_3)^,^[^7^](#_ENREF_7) Therefore, to reproduce the structure and properties of native hard tissues at multiple scales, one may not be limited to single materials or fabrication methods. (3) The restoration of enamel and dentin in the oral cavity takes place in the presence of bacteria. Thus, antibacterial functions of bioinspired materials are required during biomineralization-inspired repair.[^158^](#_ENREF_158)^,^[^297^](#_ENREF_297)^,^[^348^](#_ENREF_348)^,^[^349^](#_ENREF_349) (4) The combined use of biomineralization-inspired materials with growth factors, cells, or drugs can be considered to achieve better repair effects (*e.g.*, simultaneous vascularization or nerve regeneration when repairing bone).[^350^](#_ENREF_350)^,^[^351^](#_ENREF_351) (5) Developing novel intrafibrillar mineralization-feasible organic matrices as collagen alternatives is a potential future direction to avoid the immunogenicity caused by allogenic and xenogenic collagen products.[^10^](#_ENREF_10)

In summary, learning from nature is an eternal theme. In the coming decades, more knowledge about biomineralization will be acquired with advances in nanoscience, molecular biology, and mineral crystallography. Biomineralization-inspired materials will contribute substantially to hard tissue repair in the future.

Acknowledgements

This work was supported by the National Natural Science Foundation of China (Grant Nos. 51925304 and 51903175) and the Postdoctoral Science Foundation of China (Grant No. 2019M663503).

Additional information

**Competing interests:** The authors declare no competing interests.

References

1. Omelon, S. J. & Grynpas, M. D. Relationships between polyphosphate chemistry, biochemistry and apatite biomineralization. *Chem. Rev.* **108**, 4694-4715 (2008).

2. Kunz, W. & Kellermeier, M. Beyond biomineralization. *Science* **323**, 344 (2009).

3. Ding, C., Chen, Z. & Li, J. From molecules to macrostructures: Recent development of bioinspired hard tissue repair. *Biomater. Sci.* **5**, 1435-1449 (2017).

4. Sommerdijk, N. A. J. M. & With, G. d. Biomimetic CaCO_3_ mineralization using designer molecules and interfaces. *Chem. Rev.* **108**, 4499-4550 (2008).

5. Palmer, L. C., Newcomb, C. J., Kaltz, S. R., Spoerke, E. D. & Stupp, S. I. Biomimetic systems for hydroxyapatite mineralization inspired by bone and enamel. *Chem. Rev.* **108**, 4754-4783 (2008).

6. Xu, X., Chen, X. & Li, J. Natural protein bioinspired materials for regeneration of hard tissues. *J. Mater. Chem. B* **8**, 2199-2215 (2020).

7. Koons, G. L., Diba, M. & Mikos, A. G. Materials design for bone-tissue engineering. *Nat. Rev. Mater.* **5**, 584-603 (2020).

8. Ruan, Q. & Moradian-Oldak, J. Amelogenin and enamel biomimetics. *J. Mater. Chem. B* **3**, 3112-3129 (2015).

9. Yao, S. *et al.* Biomineralization: From material tactics to biological strategy. *Adv. Mater.* **29**, 1605903 (2017).

10. de Melo Pereira, D. & Habibovic, P. Biomineralization-inspired material design for bone regeneration. *Adv. Healthc. Mater.* **7**, 1800700 (2018).

11. Tertuliano, O. A. & Greer, J. R. The nanocomposite nature of bone drives its strength and damage resistance. *Nat. Mater.* **15**, 1195-1202 (2016).

12. Elsharkawy, S. *et al.* Protein disorder–order interplay to guide the growth of hierarchical mineralized structures. *Nat. Commun.* **9**, 2145 (2018).

13. Xie, J. *et al.* Bioprocess-inspired fabrication of materials with new structures and functions. *Prog. Mater Sci.* **105**, 100571 (2019).

14. Mu, Z. *et al.* Pressure-driven fusion of amorphous particles into integrated monoliths. *Science* **372**, 1466 (2021).

15. Dey, A., de With, G. & Sommerdijk, N. A. J. M. *In situ* techniques in biomimetic mineralization studies of calcium carbonate. *Chem. Soc. Rev.* **39**, 397-409 (2010).

16. Crookes-Goodson, W. J., Slocik, J. M. & Naik, R. R. Bio-directed synthesis and assembly of nanomaterials. *Chem. Soc. Rev.* **37**, 2403-2412 (2008).

17. Reznikov, N., Steele, J. A. M., Fratzl, P. & Stevens, M. M. A materials science vision of extracellular matrix mineralization. *Nat. Rev. Mater.* **1**, 16041 (2016).

18. Addadi, L. & Weiner, S. Interactions between acidic proteins and crystals: Stereochemical requirements in biomineralization. *Proc. Natl. Acad. Sci. U. S. A.* **82**, 4110 (1985).

19. Sharma, V., Srinivasan, A., Nikolajeff, F. & Kumar, S. Biomineralization process in hard tissues: The interaction complexity within protein and inorganic counterparts. *Acta Biomater.* **120**, 20-37 (2021).

20. Li, H., Xin, H. L., Muller, D. A. & Estroff, L. A. Visualizing the 3D internal structure of calcite single crystals grown in agarose hydrogels. *Science* **326**, 1244 (2009).

21. Zou, Z. *et al.* A hydrated crystalline calcium carbonate phase: Calcium carbonate hemihydrate. *Science* **363**, 396 (2019).

22. Mann, S. Molecular recognition in biomineralization. *Nature* **332**, 119-124 (1988).

23. Bai, Y. *et al.* Protein nanoribbons template enamel mineralization. *Proc. Natl. Acad. Sci. U. S. A.* **117**, 19201 (2020).

24. Veis, A. A window on biomineralization. *Science* **307**, 1419 (2005).

25. Wald, T. *et al.* Intrinsically disordered proteins drive enamel formation *via* an evolutionarily conserved self-assembly motif. *Proc. Natl. Acad. Sci. U. S. A.* **114**, E1641 (2017).

26. Mao, L. *et al.* Synthetic nacre by predesigned matrix-directed mineralization. *Science* **354**, 107 (2016).

27. Dickerson, M. B., Sandhage, K. H. & Naik, R. R. Protein- and peptide-directed syntheses of inorganic materials. *Chem. Rev.* **108**, 4935-4978 (2008).

28. Evans, J. S. “Tuning in” to mollusk shell nacre- and prismatic-associated protein terminal sequences. Implications for biomineralization and the construction of high performance inorganic−organic composites. *Chem. Rev.* **108**, 4455-4462 (2008).

29. Krajina, B. A., Proctor, A. C., Schoen, A. P., Spakowitz, A. J. & Heilshorn, S. C. Biotemplated synthesis of inorganic materials: An emerging paradigm for nanomaterial synthesis inspired by nature. *Prog. Mater Sci.* **91**, 1-23 (2018).

30. Lopes, D., Martins-Cruz, C., Oliveira, M. B. & Mano, J. F. Bone physiology as inspiration for tissue regenerative therapies. *Biomaterials* **185**, 240-275 (2018).

31. Huang, W. *et al.* Multiscale toughening mechanisms in biological materials and bioinspired designs. *Adv. Mater.* **31**, 1901561 (2019).

32. Wegst, U. G. K., Bai, H., Saiz, E., Tomsia, A. P. & Ritchie, R. O. Bioinspired structural materials. *Nat. Mater.* **14**, 23-36 (2015).

33. Bonucci, E. Bone mineralization. *Front. Biosci., Landmark Ed.* **17**, 100-128 (2012).

34. Murshed, M. Mechanism of Bone Mineralization. *Cold Spring Harb. Perspect. Med.* **8**, a031229 (2018).

35. Kim, D., Lee, B., Thomopoulos, S. & Jun, Y.-S. The role of confined collagen geometry in decreasing nucleation energy barriers to intrafibrillar mineralization. *Nat. Commun.* **9**, 962 (2018).

36. George, A. & Veis, A. Phosphorylated proteins and control over apatite nucleation, crystal growth, and inhibition. *Chem. Rev.* **108**, 4670-4693 (2008).

37. Yang, W., Meyers, M. A. & Ritchie, R. O. Structural architectures with toughening mechanisms in nature: A review of the materials science of type-I collagenous materials. *Prog. Mater Sci.* **103**, 425-483 (2019).

38. Gupta, H. S. *et al.* Cooperative deformation of mineral and collagen in bone at the nanoscale. *Proc. Natl. Acad. Sci. U. S. A.* **103**, 17741 (2006).

39. Mahamid, J., Sharir, A., Addadi, L. & Weiner, S. Amorphous calcium phosphate is a major component of the forming fin bones of zebrafish: Indications for an amorphous precursor phase. *Proc. Natl. Acad. Sci. U. S. A.* **105**, 12748 (2008).

40. Mahamid, J. *et al.* Mapping amorphous calcium phosphate transformation into crystalline mineral from the cell to the bone in zebrafish fin rays. *Proc. Natl. Acad. Sci. U. S. A.* **107**, 6316 (2010).

41. Kerschnitzki, M. *et al.* Bone mineralization pathways during the rapid growth of embryonic chicken long bones. *J. Struct. Biol.* **195**, 82-92 (2016).

42. Olszta, M. J. *et al.* Bone structure and formation: A new perspective. *Mater. Sci. Eng., R* **58**, 77-116 (2007).

43. Lotsari, A., Rajasekharan, A. K., Halvarsson, M. & Andersson, M. Transformation of amorphous calcium phosphate to bone-like apatite. *Nat. Commun.* **9**, 4170 (2018).

44. Gower, L. B. Biomimetic model systems for investigating the amorphous precursor pathway and its role in biomineralization. *Chem. Rev.* **108**, 4551-4627 (2008).

45. Jehannin, M., Rao, A. & Cölfen, H. New horizons of nonclassical crystallization. *J. Am. Chem. Soc.* **141**, 10120-10136 (2019).

46. De Yoreo, J. J. *et al.* Crystallization by particle attachment in synthetic, biogenic, and geologic environments. *Science* **349**, aaa6760 (2015).

47. Athanasiadou, D. & Carneiro, K. M. M. DNA nanostructures as templates for biomineralization. *Nat. Rev. Chem.* **5**, 93-108 (2021).

48. Meldrum, F. C. & Cölfen, H. Controlling mineral morphologies and structures in biological and synthetic systems. *Chem. Rev.* **108**, 4332-4432 (2008).

49. Jiao, K. *et al.* Complementarity and uncertainty in intrafibrillar mineralization of collagen. *Adv. Funct. Mater.* **26**, 6858-6875 (2016).

50. Niu, L. *et al.* Collagen intrafibrillar mineralization as a result of the balance between osmotic equilibrium and electroneutrality. *Nat. Mater.* **16**, 370-378 (2017).

51. Oosterlaken, B. M., Vena, M. P. & de With, G. *In vitro* mineralization of collagen. *Adv. Mater.* **33**, 2004418 (2021).

52. Yu, L. & Wei, M. Biomineralization of collagen-based materials for hard tissue repair. *Int. J. Mol. Sci.* **22**, 944 (2021).

53. Lenton, S., Wang, Q., Nylander, T., Teixeira, S. & Holt, C. Structural biology of calcium phosphate nanoclusters sequestered by phosphoproteins. *Crystals* **10**, 755 (2020).

54. Olszta, M. J. *et al.* Bone structure and formation: A new perspective. *Mater. Sci. Eng., R* **58**, 77-116 (2007).

55. Deshpande, A. S. & Beniash, E. Bioinspired synthesis of mineralized collagen fibrils. *Cryst. Growth Des.* **8**, 3084-3090 (2008).

56. Tay, F. R. & Pashley, D. H. Guided tissue remineralisation of partially demineralised human dentine. *Biomaterials* **29**, 1127-1137 (2008).

57. Liu, Y., Luo, D. & Wang, T. Hierarchical structures of bone and bioinspired bone tissue engineering. *Small* **12**, 4611-4632 (2016).

58. Huang, X. *et al.* Hollow mesoporous zirconia delivery system for biomineralization precursors. *Acta Biomater.* **67**, 366-377 (2018).

59. Du, T. *et al.* Highly aligned hierarchical intrafibrillar mineralization of collagen induced by periodic fluid shear stress. *J. Mater. Chem. B* **8**, 2562-2572 (2020).

60. Niu, X. *et al.* Shear-mediated orientational mineralization of bone apatite on collagen fibrils. *J. Mater. Chem. B* **5**, 9141-9147 (2017).

61. Wang, Y. *et al.* The predominant role of collagen in the nucleation, growth, structure and orientation of bone apatite. *Nat. Mater.* **11**, 724-733 (2012).

62. Cui, F., Li, Y. & Ge, J. Self-assembly of mineralized collagen composites. *Mater. Sci. Eng., R* **57**, 1-27 (2007).

63. Zhang, W., Liao, S. & Cui, F. Hierarchical self-assembly of nano-fibrils in mineralized collagen. *Chem. Mater.* **15**, 3221-3226 (2003).

64. Ma, Y. *et al.* Involvement of prenucleation clusters in calcium phosphate mineralization of collagen. *Acta Biomater.* **120**, 213-223 (2021).

65. Song, Q. *et al.* Contribution of biomimetic collagen-ligand interaction to intrafibrillar mineralization. *Sci. Adv.* **5**, eaav9075 (2019).

66. Wu, S., Liu, X., Yeung, K. W. K., Liu, C. & Yang, X. Biomimetic porous scaffolds for bone tissue engineering. *Mater. Sci. Eng., R* **80**, 1-36 (2014).

67. Zhang, Y. *et al.* Polymer fiber scaffolds for bone and cartilage tissue engineering. *Adv. Funct. Mater.* **29**, 1903279 (2019).

68. Yi, H., Ur Rehman, F., Zhao, C., Liu, B. & He, N. Recent advances in nano scaffolds for bone repair. *Bone Res.* **4**, 16050 (2016).

69. Griffanti, G. & Nazhat, S. N. Dense fibrillar collagen-based hydrogels as functional osteoid-mimicking scaffolds. *Int. Mater. Rev.* **65**, 502-521 (2020).

70. Ho-Shui-Ling, A. *et al.* Bone regeneration strategies: Engineered scaffolds, bioactive molecules and stem cells current stage and future perspectives. *Biomaterials* **180**, 143-162 (2018).

71. Garot, C., Bettega, G. & Picart, C. Additive manufacturing of material scaffolds for bone regeneration: Toward application in the clinics. *Adv. Funct. Mater.* **31**, 2006967 (2021).

72. Pina, S., Oliveira, J. M. & Reis, R. L. Natural-based nanocomposites for bone tissue engineering and regenerative medicine: A review. *Adv. Mater.* **27**, 1143-1169 (2015).

73. Zhang, D., Wu, X., Chen, J. & Lin, K. The development of collagen based composite scaffolds for bone regeneration. *Bioact. Mater.* **3**, 129-138 (2018).

74. Li, Z., Du, T., Ruan, C. & Niu, X. Bioinspired mineralized collagen scaffolds for bone tissue engineering. *Bioact. Mater.* **6**, 1491-1511 (2021).

75. Du, M., Chen, J., Liu, K., Xing, H. & Song, C. Recent advances in biomedical engineering of nano-hydroxyapatite including dentistry, cancer treatment and bone repair. *Compos. B Eng.* **215**, 108790 (2021).

76. Du, Y., Guo, J. L., Wang, J., Mikos, A. G. & Zhang, S. Hierarchically designed bone scaffolds: From internal cues to external stimuli. *Biomaterials* **218**, 119334 (2019).

77. He, W., Rajasekharan, A. K., Tehrani-Bagha, A. R. & Andersson, M. Mesoscopically ordered bone-mimetic nanocomposites. *Adv. Mater.* **27**, 2260-2264 (2015).

78. Liu, Y. *et al.* Thermodynamically controlled self-assembly of hierarchically staggered architecture as an osteoinductive alternative to bone autografts. *Adv. Funct. Mater.* **29**, 1806445 (2019).

79. Liu, Y. *et al.* Hierarchically Staggered Nanostructure of Mineralized Collagen as a Bone-Grafting Scaffold. *Adv. Mater.* **28**, 8740-8748 (2016).

80. Yu, L. *et al.* Intrafibrillar mineralized collagen–hydroxyapatite-based scaffolds for bone regeneration. *ACS Appl. Mater. Interfaces* **12**, 18235-18249 (2020).

81. Thula, T. T. *et al.* *In vitro* mineralization of dense collagen substrates: A biomimetic approach toward the development of bone-graft materials. *Acta Biomater.* **7**, 3158-3169 (2011).

82. Thrivikraman, G. *et al.* Rapid fabrication of vascularized and innervated cell-laden bone models with biomimetic intrafibrillar collagen mineralization. *Nat. Commun.* **10**, 3520 (2019).

83. Milazzo, M., Jung, G. S., Danti, S. & Buehler, M. J. Mechanics of mineralized collagen fibrils upon transient loads. *ACS Nano* **14**, 8307-8316 (2020).

84. Liu, Y. *et al.* Intrafibrillar collagen mineralization produced by biomimetic hierarchical nanoapatite assembly. *Adv. Mater.* **23**, 975-980 (2011).

85. Jee, S. S., Thula, T. T. & Gower, L. B. Development of bone-like composites *via* the polymer-induced liquid-precursor (PILP) process. Part 1: Influence of polymer molecular weight. *Acta Biomater.* **6**, 3676-3686 (2010).

86. Zhang, J. *et al.* Ionic colloidal molding as a biomimetic scaffolding strategy for uniform bone tissue regeneration. *Adv. Mater.* **29**, 1605546 (2017).

87. Olszta, M. J., Douglas, E. P. & Gower, L. B. Intrafibrillar mineralization of collagen using a liquid-phase mineral precursor. *Mater. Res. Soc. Symp. Proc.* **774**, 127 (2003).

88. Olszta, M. J., Douglas, E. P. & Gower, L. B. Scanning electron microscopic analysis of the mineralization of type I collagen *via* a polymer-induced liquid-precursor (PILP) process. *Calcif. Tissue Int.* **72**, 583-591 (2003).

89. Liu, Y. *et al.* Hierarchical and non-hierarchical mineralisation of collagen. *Biomaterials* **32**, 1291-1300 (2011).

90. Lausch, A. J., Chong, L. C., Uludag, H. & Sone, E. D. Multiphasic collagen scaffolds for engineered tissue interfaces. *Adv. Funct. Mater.* **28**, 1804730 (2018).

91. Niu, L. *et al.* Infiltration of silica inside fibrillar collagen. *Angew. Chem. Int. Ed.* **50**, 11688-11691 (2011).

92. Niu, L. *et al.* Multiphase intrafibrillar mineralization of collagen. *Angew. Chem. Int. Ed.* **52**, 5762-5766 (2013).

93. Niu, L. *et al.* Biomimetic silicification of demineralized hierarchical collagenous tissues. *Biomacromolecules* **14**, 1661-1668 (2013).

94. Sun, J. *et al.* Intrafibrillar silicified collagen scaffold promotes *in situ* bone regeneration by activating the monocyte p38 signaling pathway. *Acta Biomater.* **67**, 354-365 (2018).

95. Sun, J. *et al.* Intrafibrillar silicified collagen scaffold modulates monocyte to promote cell homing, angiogenesis and bone regeneration. *Biomaterials* **113**, 203-216 (2017).

96. Jiao, K. *et al.* Biphasic silica/apatite co-mineralized collagen scaffolds stimulate osteogenesis and inhibit RANKL-mediated osteoclastogenesis. *Acta Biomater.* **19**, 23-32 (2015).

97. Niu, L. *et al.* Intrafibrillar silicification of collagen scaffolds for sustained release of stem cell homing chemokine in hard tissue regeneration. *FASEB J.* **26**, 4517-4529 (2012).

98. Zhou, B. *et al.* Adopting the principles of collagen biomineralization for intrafibrillar infiltration of yttria-stabilized zirconia into three-dimensional collagen scaffolds. *Adv. Funct. Mater.* **24**, 1895-1903 (2014).

99. Wang, R. *et al.* Synthesis of nanophase hydroxyapatite/collagen composite. *J. Mater. Sci. Lett.* **14**, 490-492 (1995).

100. Bradt, J., Mertig, M., Teresiak, A. & Pompe, W. Biomimetic mineralization of collagen by combined fibril assembly and calcium phosphate formation. *Chem. Mater.* **11**, 2694-2701 (1999).

101. Cui, Y., Cui, H. & Wang, X. in Mineralized Collagen Bone Graft Substitutes (eds Wang, X., Qiu, Z., & Cui, H.) Ch. 2 (Elsevier Ltd., 2019).

102. Wang, Y. *et al.* Investigations of the initial stage of recombinant human-like collagen mineralization. *Mater. Sci. Eng., C* **26**, 635-638 (2006).

103. Shi, X. *et al.* The observed difference of RAW264.7 macrophage phenotype on mineralized collagen and hydroxyapatite. *Biomed. Mater.* **13**, 041001 (2018).

104. Liu, F. *et al.* Comparison of rabbit rib defect regeneration with and without graft. *J. Mater. Sci.: Mater. Med.* **28**, 2 (2016).

105. Wang, S. *et al.* A high-strength mineralized collagen bone scaffold for large-sized cranial bone defect repair in sheep. *Regener. Biomater.* **5**, 283-292 (2018).

106. Pan, Y., Yang, G., Li, Z., Shi, Z. & Sun, Z. Clinical observation of biomimetic mineralized collagen artificial bone putty for bone reconstruction of calcaneus fracture. *Regener. Biomater.* **5**, 61-67 (2018).

107. Gao, C. *et al.* Clinical observation of mineralized collagen bone grafting after curettage of benign bone tumors. *Regener. Biomater.* **7**, 567-575 (2020).

108. Luo, K. *et al.* Poly(methyl methacrylate) bone cement composited with mineralized collagen for osteoporotic vertebral compression fractures in extremely old patients. *Regener. Biomater.* **7**, 29-34 (2020).

109. Liao, S., Cui, F., Zhang, W. & Feng, Q. Hierarchically biomimetic bone scaffold materials: Nano-HA/collagen/PLA composite. *J. Biomed. Mater. Res. B* **69B**, 158-165 (2004).

110. Wang, S. *et al.* Tuning pore features of mineralized collagen/PCL scaffolds for cranial bone regeneration in a rat model. *Mater. Sci. Eng., C* **106**, 110186 (2020).

111. Zhang, S., Cui, F., Liao, S., Zhu, Y. & Han, L. Synthesis and biocompatibility of porous nano-hydroxyapatite/collagen/alginate composite. *J. Mater. Sci.: Mater. Med.* **14**, 641-645 (2003).

112. Zhu, J. *et al.* Mineralized collagen modified polymethyl methacrylate bone cement for osteoporotic compression vertebral fracture at 1-year follow-up. *Spine* **44**, 827-838 (2019).

113. Chen, Z. *et al.* Degradability of injectable calcium sulfate/mineralized collagen-based bone repair material and its effect on bone tissue regeneration. *Mater. Sci. Eng., C* **45**, 94-102 (2014).

114. Zhang, X. *et al.* *In vitro* and *in vivo* enhancement of osteogenic capacity in a synthetic BMP-2 derived peptide-coated mineralized collagen composite. *J. Tissue Eng. Regener. Med.* **10**, 99-107 (2016).

115. Liu, X. *et al.* Repairing goat tibia segmental bone defect using scaffold cultured with mesenchymal stem cells. *J. Biomed. Mater. Res. B* **94B**, 44-52 (2010).

116. Song, T. *et al.* in Mineralized Collagen Bone Graft Substitutes (eds Wang, X., Qiu, Z., & Cui, H.) Ch. 5 (Elsevier Ltd., 2019).

117. Ai, W., Hu, Y., He, Z., Song, T. & Wang, Z. in Mineralized Collagen Bone Graft Substitutes (eds Wang, X., Qiu, Z., & Cui, H.) Ch. 4 (Elsevier Ltd., 2019).

118. Wang, Z., Cui, Y. & Qiu, Z. in Mineralized Collagen Bone Graft Substitutes (eds Wang, X., Qiu, Z., & Cui, H.) Ch. 3 (Elsevier Ltd., 2019).

119. Li, Y., Chen, X., Fok, A., Rodriguez-Cabello, J. C. & Aparicio, C. Biomimetic mineralization of recombinamer-based hydrogels toward controlled morphologies and high mineral density. *ACS Appl. Mater. Interfaces* **7**, 25784-25792 (2015).

120. Li, Y., Rodriguez-Cabello, J. C. & Aparicio, C. Intrafibrillar mineralization of self-assembled elastin-like recombinamer fibrils. *ACS Appl. Mater. Interfaces* **9**, 5838-5846 (2017).

121. Qi, Y., Cheng, Z., Ye, Z., Zhu, H. & Aparicio, C. Bioinspired mineralization with hydroxyapatite and hierarchical naturally aligned nanofibrillar cellulose. *ACS Appl. Mater. Interfaces* **11**, 27598-27604 (2019).

122. Yu, Y. *et al.* Biomimetic mineralized organic–inorganic hybrid macrofiber with spider silk-like supertoughness. *Adv. Funct. Mater.* **30**, 1908556 (2020).

123. Yao, S. *et al.* Osteoporotic bone recovery by a highly bone-inductive calcium phosphate polymer-induced liquid-precursor. *Adv. Sci.* **6**, 1900683 (2019).

124. Cao, C. Y., Mei, M. L., Li, Q., Lo, E. C. M. & Chu, C. H. Methods for biomimetic remineralization of human dentine: A systematic review. *Int. J. Mol. Sci.* **16**, 4615-4627 (2015).

125. Niu, L. *et al.* Biomimetic remineralization of dentin. *Dent. Mater.* **30**, 77-96 (2014).

126. Zhong, B. *et al.* Contemporary research findings on dentine remineralization. *J. Tissue Eng. Regener. Med.* **9**, 1004-1016 (2015).

127. Shahmoradi, M., Bertassoni, L. E., Elfallah, H. M. & Swain, M. in Advances in Calcium Phosphate Biomaterials (ed Ben-Nissan, B.) Ch. 17 (Springer, 2014).

128. Bleicher, F., Richard, B., Thivichon-Prince, B., Farges, J. C. & Carrouel, F. in Stem Cell Biology and Tissue Engineering in Dental Sciences (eds Vishwakarma, A., Sharpe, P., Shi, S., & Ramalingam, M.) Ch. 30 (Elsevier Inc., 2015).

129. El Gezawi, M., Wölfle, U. C., Haridy, R., Fliefel, R. & Kaisarly, D. Remineralization, regeneration, and repair of natural tooth structure: Influences on the future of restorative dentistry practice. *ACS Biomater. Sci. Eng.* **5**, 4899-4919 (2019).

130. Prasad, M., Butler, W. T. & Qin, C. Dentin sialophosphoprotein in biomineralization. *Connect. Tissue Res.* **51**, 404-417 (2010).

131. Liang, K. *et al.* 8DSS-promoted remineralization of demineralized dentin *in vitro*. *J. Mater. Chem. B* **3**, 6763-6772 (2015).

132. Li, J. *et al.* Bioinspired intrafibrillar mineralization of human dentine by PAMAM dendrimer. *Biomaterials* **34**, 6738-6747 (2013).

133. Shao, C. *et al.* Citrate improves collagen mineralization *via* interface wetting: A physicochemical understanding of biomineralization control. *Adv. Mater.* **30**, 1704876 (2018).

134. Li, C., Lu, D., Deng, J., Zhang, X. & Yang, P. Amyloid-Like rapid surface modification for antifouling and in-depth remineralization of dentine tubules to treat dental hypersensitivity. *Adv. Mater.* **31**, 1903973 (2019).

135. Milan, A. M., Sugars, R. V., Embery, G. & Waddington, R. J. Adsorption and interactions of dentine phosphoprotein with hydroxyapatite and collagen. *Eur. J. Oral Sci.* **114**, 223-231 (2006).

136. Yarbrough, D. K. *et al.* Specific binding and mineralization of calcified surfaces by small peptides. *Calcif. Tissue Int.* **86**, 58-66 (2010).

137. Liang, K. *et al.* 8DSS peptide induced effective dentinal tubule occlusion *in vitro*. *Dent. Mater.* **34**, 629-640 (2018).

138. Cao, Y. *et al.* A novel oligopeptide simulating dentine matrix protein 1 for biomimetic mineralization of dentine. *Clin. Oral. Investig.* **18**, 873-881 (2014).

139. He, G., Dahl, T., Veis, A. & George, A. Nucleation of apatite crystals *in vitro* by self-assembled dentin matrix protein 1. *Nat. Mater.* **2**, 552-558 (2003).

140. Padovano, J. D. *et al.* DMP1-derived peptides promote remineralization of human dentin. *J. Dent. Res.* **94**, 608-614 (2015).

141. Ling, Z. *et al.* Effects of oligopeptide simulating DMP-1/mineral trioxide aggregate/agarose hydrogel biomimetic mineralisation model for the treatment of dentine hypersensitivity. *J. Mater. Chem. B* **7**, 5825-5833 (2019).

142. Wang, Q., Wang, X., Tian, L., Cheng, Z. & Cui, F. *In situ* remineralizaiton of partially demineralized human dentine mediated by a biomimetic non-collagen peptide. *Soft Matter* **7**, 9673-9680 (2011).

143. Deshpande, A. S., Fang, P., Simmer, J. P., Margolis, H. C. & Beniash, E. Amelogenin-collagen interactions regulate calcium phosphate mineralization *in vitro*. *J. Biol. Chem.* **285**, 19277-19287 (2010).

144. Mukherjee, K., Visakan, G., Phark, J. H. & Moradian-Oldak, J. Enhancing collagen mineralization with amelogenin peptide: Toward the restoration of dentin. *ACS Biomater. Sci. Eng.* **6**, 2251-2262 (2020).

145. Wang, Q. *et al.* A novel amphiphilic oligopeptide induced the intrafibrillar mineralisation *via* interacting with collagen and minerals. *J. Mater. Chem. B* **8**, 2350-2362 (2020).

146. Duncan, R. & Izzo, L. Dendrimer biocompatibility and toxicity. *Adv. Drug Deliv. Rev.* **57**, 2215-2237 (2005).

147. Nimesh, S. in Gene Therapy (ed Nimesh, S.) Ch. 13 (Woodhead Publishing Ltd., 2013).

148. Yang, S., He, H., Wang, L., Jia, X. & Feng, H. Oriented crystallization of hydroxyapatite by the biomimetic amelogenin nanospheres from self-assemblies of amphiphilic dendrons. *Chem. Commun.* **47**, 10100-10102 (2011).

149. Yang, J. *et al.* Staged self-assembly of PAMAM dendrimers into macroscopic aggregates with a microribbon structure similar to that of amelogenin. *Soft Matter* **9**, 7553-7559 (2013).

150. Esfand, R. & Tomalia, D. A. Poly(amidoamine) (PAMAM) dendrimers: From biomimicry to drug delivery and biomedical applications. *Drug Discov. Today* **6**, 427-436 (2001).

151. Naka, K., Kobayashi, A. & Chujo, Y. Effect of anionic 4.5-generation polyamidoamine dendrimer on the formation of calcium carbonate polymorphs. *Bull. Chem. Soc. Jpn.* **75**, 2541-2546 (2002).

152. Khopade, A. J., Khopade, S. & Jain, N. K. Development of hemoglobin aquasomes from spherical hydroxyapatite cores precipitated in the presence of half-generation poly(amidoamine) dendrimer. *Int. J. Pharm.* **241**, 145-154 (2002).

153. Yang, X., Shang, H., Ding, C. & Li, J. Recent developments and applications of bioinspired dendritic polymers. *Polym. Chem.* **6**, 668-680 (2015).

154. Tao, S. *et al.* The remineralization effectiveness of PAMAM dendrimer with different terminal groups on demineralized dentin *in vitro*. *RSC Adv.* **7**, 54947-54955 (2017).

155. Zhou, Y. *et al.* Triclosan-loaded poly(amido amine) dendrimer for simultaneous treatment and remineralization of human dentine. *Colloids Surf., B* **115**, 237-243 (2014).

156. Xie, F., Wei, X., Li, Q. & Zhou, T. *In vivo* analyses of the effects of polyamidoamine dendrimer on dentin biomineralization and dentinal tubules occlusion. *Dent. Mater. J.* **35**, 104-111 (2016).

157. Zhang, H. *et al.* Effective dentin restorative material based on phosphate-terminated dendrimer as artificial protein. *Colloids Surf., B* **128**, 304-314 (2015).

158. Zhu, B. *et al.* One-step phosphorylated poly(amide-amine) dendrimer loaded with apigenin for simultaneous remineralization and antibacterial of dentine. *Colloids Surf., B* **172**, 760-768 (2018).

159. Wang, T., Yang, S., Wang, L. & Feng, H. Use of multifunctional phosphorylated PAMAM dendrimers for dentin biomimetic remineralization and dentinal tubule occlusion. *RSC Adv.* **5**, 11136-11144 (2015).

160. Jia, R., Lu, Y., Yang, C., Luo, X. & Han, Y. Effect of generation 4.0 polyamidoamine dendrimer on the mineralization of demineralized dentinal tubules *in vitro*. *Arch. Oral Biol.* **59**, 1085-1093 (2014).

161. Liang, K. *et al.* Biomimetic mineralization of collagen fibrils induced by amine-terminated PAMAM dendrimers—PAMAM dendrimers for remineralization. *J. Biomater. Sci., Polym. Ed.* **26**, 963-974 (2015).

162. Liang, K. *et al.* Remineralization of demineralized dentin induced by amine-terminated PAMAM dendrimer. *Macromol. Mater. Eng.* **300**, 107-117 (2015).

163. Gao, Y. *et al.* Effect and stability of poly(amido amine)-induced biomineralization on dentinal tubule occlusion. *Materials* **10**, 384 (2017).

164. Wang, T., Yang, S., Wang, L. & Feng, H. Use of poly (amidoamine) dendrimer for dentinal tubule occlusion: A preliminary study. *PLoS One* **10**, e0124735 (2015).

165. Liang, K. *et al.* Effective dentinal tubule occlusion induced by polyhydroxy-terminated PAMAM dendrimer *in vitro*. *RSC Adv.* **4**, 43496-43503 (2014).

166. Liang, K. *et al.* Poly (amido amine) and nano-calcium phosphate bonding agent to remineralize tooth dentin in cyclic artificial saliva/lactic acid. *Mater. Sci. Eng., C* **72**, 7-17 (2017).

167. Xiao, S. *et al.* Combining bioactive multifunctional dental composite with PAMAM for root dentin remineralization. *Materials* **10**, 89 (2017).

168. Liang, K. *et al.* Poly(amido amine) and rechargeable adhesive containing calcium phosphate nanoparticles for long-term dentin remineralization. *J. Dent.* **85**, 47-56 (2019).

169. Liang, K. *et al.* Poly (amido amine) dendrimer and dental adhesive with calcium phosphate nanoparticles remineralized dentin in lactic acid. *J. Biomed. Mater. Res. B* **106**, 2414-2424 (2018).

170. Liang, K. *et al.* Long-term dentin remineralization by poly(amido amine) and rechargeable calcium phosphate nanocomposite after fluid challenges. *Dent. Mater.* **34**, 607-618 (2018).

171. Liang, K. *et al.* Poly(amido amine) and calcium phosphate nanocomposite remineralization of dentin in acidic solution without calcium phosphate ions. *Dent. Mater.* **33**, 818-829 (2017).

172. Liang, K. *et al.* Dentin remineralization in acid challenge environment *via* PAMAM and calcium phosphate composite. *Dent. Mater.* **32**, 1429-1440 (2016).

173. Lin, X. *et al.* Fabrication and characterization of dendrimer-functionalized nano-hydroxyapatite and its application in dentin tubule occlusion. *J. Biomater. Sci., Polym. Ed.* **28**, 846-863 (2017).

174. Bae, J. *et al.* Effects of poly(amidoamine) dendrimer-coated mesoporous bioactive glass nanoparticles on dentin remineralization. *Nanomaterials* **9**, 591 (2019).

175. Liang, K. *et al.* Dental remineralization *via* poly(amido amine) and restorative materials containing calcium phosphate nanoparticles. *Int. J. Oral. Sci.* **11**, 15 (2019).

176. Nudelman, F. *et al.* The role of collagen in bone apatite formation in the presence of hydroxyapatite nucleation inhibitors. *Nat. Mater.* **9**, 1004-1009 (2010).

177. Burwell, A. K. *et al.* Functional remineralization of dentin lesions using polymer-induced liquid-precursor process. *PLoS One* **7**, e38852 (2012).

178. Chen, Y. *et al.* Hierarchical structure and mechanical properties of remineralized dentin. *J. Mech. Behav. Biomed. Mater.* **40**, 297-306 (2014).

179. Wang, J. *et al.* Remineralization of dentin collagen by meta-stabilized amorphous calcium phosphate. *CrystEngComm* **15**, 6151-6158 (2013).

180. Chen, R. *et al.* Biomimetic remineralization of artificial caries dentin lesion using Ca/P-PILP. *Dent. Mater.* **36**, 1397-1406 (2020).

181. Sun, J. *et al.* Biomimetic promotion of dentin remineralization using _L_-glutamic acid: Inspiration from biomineralization proteins. *J. Mater. Chem. B* **2**, 4544-4553 (2014).

182. Zhao, L. *et al.* Effect of aspartic acid on the crystallization kinetics of ACP and dentin remineralization. *J. Mech. Behav. Biomed. Mater.* **115**, 104226 (2021).

183. Chen, C. *et al.* Glutaraldehyde-induced remineralization improves the mechanical properties and biostability of dentin collagen. *Mater. Sci. Eng., C* **67**, 657-665 (2016).

184. He, H. *et al.* Promotion effect of immobilized chondroitin sulfate on intrafibrillar mineralization of collagen. *Carbohydr. Polym.* **229**, 115547 (2020).

185. Qu, Y. *et al.* Polydopamine promotes dentin remineralization *via* interfacial control. *ACS Biomater. Sci. Eng.* **6**, 3327-3334 (2020).

186. Luo, X. *et al.* Translation of a solution-based biomineralization concept into a carrier-based delivery system *via* the use of expanded-pore mesoporous silica. *Acta Biomater.* **31**, 378-387 (2016).

187. Zhang, W. *et al.* Biomimetic intrafibrillar mineralization of type I collagen with intermediate precursors-loaded mesoporous carriers. *Sci. Rep.* **5**, 11199 (2015).

188. Wang, Z. *et al.* A novel fluorescent adhesive-assisted biomimetic mineralization. *Nanoscale* **10**, 18980-18987 (2018).

189. Wu, Z. *et al.* Self-etch adhesive as a carrier for ACP nanoprecursors to deliver biomimetic remineralization. *ACS Appl. Mater. Interfaces* **9**, 17710-17717 (2017).

190. Mai, S. *et al.* Phosphoric acid esters cannot replace polyvinylphosphonic acid as phosphoprotein analogs in biomimetic remineralization of resin-bonded dentin. *Dent. Mater.* **25**, 1230-1239 (2009).

191. Gu, L. *et al.* Changes in stiffness of resin-infiltrated demineralized dentin after remineralization by a bottom-up biomimetic approach. *Acta Biomater.* **6**, 1453-1461 (2010).

192. Kim, Y. K. *et al.* Biomimetic remineralization as a progressive dehydration mechanism of collagen matrices—Implications in the aging of resin–dentin bonds. *Acta Biomater.* **6**, 3729-3739 (2010).

193. Kim, J. *et al.* Functional biomimetic analogs help remineralize apatite-depleted demineralized resin-infiltrated dentin *via* a bottom–up approach. *Acta Biomater.* **6**, 2740-2750 (2010).

194. Ryou, H. *et al.* Effect of biomimetic remineralization on the dynamic nanomechanical properties of dentin hybrid layers. *J. Dent. Res.* **90**, 1122-1128 (2011).

195. Gu, L. *et al.* A chemical phosphorylation-inspired design for type I collagen biomimetic remineralization. *Dent. Mater.* **26**, 1077-1089 (2010).

196. Liu, Y. *et al.* The use of sodium trimetaphosphate as a biomimetic analog of matrix phosphoproteins for remineralization of artificial caries-like dentin. *Dent. Mater.* **27**, 465-477 (2011).

197. Gu, L. *et al.* Biomimetic analogs for collagen biomineralization. *J. Dent. Res.* **90**, 82-87 (2010).

198. Qi, Y. *et al.* Remineralization of artificial dentinal caries lesions by biomimetically modified mineral trioxide aggregate. *Acta Biomater.* **8**, 836-842 (2012).

199. Zheng, B. *et al.* Phosphorylated chitosan to promote biomimetic mineralization of type I collagen as a strategy for dentin repair and bone tissue engineering. *New J. Chem.* **43**, 2002-2010 (2019).

200. Cantaert, B. *et al.* Think positive: Phase separation enables a positively charged additive to induce dramatic changes in calcium carbonate morphology. *Adv. Funct. Mater.* **22**, 907-915 (2012).

201. Yang, H. *et al.* Biodegradable mesoporous delivery system for biomineralization precursors. *Int J Nanomedicine* **12**, 839-854 (2017).

202. Gil de Bona, A. & Bidlack, F. Tooth enamel and its dynamic protein matrix. *Int. J. Mol. Sci.* **21**, 4458 (2020).

203. Pandya, M. *et al.* Posttranslational amelogenin processing and changes in matrix assembly during enamel development. *Front. Physiol.* **8**, 790-790 (2017).

204. Gopinathan, G. *et al.* The expanded amelogenin polyproline region preferentially binds to apatite versus carbonate and promotes apatite crystal elongation. *Front. Physiol.* **5**, 430 (2014).

205. Lacruz, R. S., Habelitz, S., Wright, J. T. & Paine, M. L. Dental enamel formation and implications for oral health and disease. *Physiol. Rev.* **97**, 939-993 (2017).

206. La Fontaine, A. *et al.* Atomic-scale compositional mapping reveals Mg-rich amorphous calcium phosphate in human dental enamel. *Sci. Adv.* **2**, e1601145 (2016).

207. Gordon, L. M. *et al.* Amorphous intergranular phases control the properties of rodent tooth enamel. *Science* **347**, 746 (2015).

208. Beniash, E. *et al.* The hidden structure of human enamel. *Nat. Commun.* **10**, 4383 (2019).

209. Yamakoshi, Y. Porcine amelogenin: Alternative splicing, proteolytic processing, protein-protein interactions, and possible functions. *J. Oral Biosci.* **53**, 275-283 (2011).

210. Fincham, A. G., Moradian-Oldak, J. & Simmer, J. P. The structural biology of the developing dental enamel matrix. *J. Struct. Biol.* **126**, 270-299 (1999).

211. Beniash, E., Metzler, R. A., Lam, R. S. K. & Gilbert, P. U. P. A. Transient amorphous calcium phosphate in forming enamel. *J. Struct. Biol.* **166**, 133-143 (2009).

212. Yang, X. *et al.* How amelogenin orchestrates the organization of hierarchical elongated microstructures of apatite. *J. Phys. Chem. B* **114**, 2293-2300 (2010).

213. Du, C., Falini, G., Fermani, S., Abbott, C. & Moradian-Oldak, J. Supramolecular assembly of amelogenin nanospheres into birefringent microribbons. *Science* **307**, 1450 (2005).

214. Fang, P., Conway, J. F., Margolis, H. C., Simmer, J. P. & Beniash, E. Hierarchical self-assembly of amelogenin and the regulation of biomineralization at the nanoscale. *Proc. Natl. Acad. Sci. U. S. A.* **108**, 14097 (2011).

215. Moradian-Oldak, J. Protein-mediated enamel mineralization. *Front. Biosci.* **17**, 1996-2023 (2012).

216. Prajapati, S., Tao, J., Ruan, Q., De Yoreo, J. J. & Moradian-Oldak, J. Matrix metalloproteinase-20 mediates dental enamel biomineralization by preventing protein occlusion inside apatite crystals. *Biomaterials* **75**, 260-270 (2016).

217. Cao, C. Y., Mei, M. L., Li, Q., Lo, E. C. M. & Chu, C. H. Methods for biomimetic mineralisation of human enamel: A systematic review. *Materials* **8**, 2873-2886 (2015).

218. Wu, D. *et al.* Hydroxyapatite-anchored dendrimer for *in situ* remineralization of human tooth enamel. *Biomaterials* **34**, 5036-5047 (2013).

219. Zhou, Y., Zhou, Y., Gao, L., Wu, C. & Chang, J. Synthesis of artificial dental enamel by an elastin-like polypeptide assisted biomimetic approach. *J. Mater. Chem. B* **6**, 844-853 (2018).

220. Totiam, P., González-Cabezas, C., Fontana, M. R. & Zero, D. T. A new *in vitro* model to study the relationship of gap size and secondary caries. *Caries Res.* **41**, 467-473 (2007).

221. Pandya, M. & Diekwisch, T. G. H. Enamel biomimetics—fiction or future of dentistry. *Int. J. Oral. Sci.* **11**, 8 (2019).

222. Chen, H. *et al.* Acellular synthesis of a human enamel-like microstructure. *Adv. Mater.* **18**, 1846-1851 (2006).

223. Yin, Y., Yun, S., Fang, J. & Chen, H. Chemical regeneration of human tooth enamel under near-physiological conditions. *Chem. Commun.*, 5892-5894 (2009).

224. Fowler, C. E., Li, M., Mann, S. & Margolis, H. C. Influence of surfactant assembly on the formation of calcium phosphate materials—A model for dental enamel formation. *J. Mater. Chem.* **15**, 3317-3325 (2005).

225. Xie, R., Feng, Z., Li, S. & Xu, B. EDTA-assisted self-assembly of fluoride-substituted hydroxyapatite coating on enamel substrate. *Cryst. Growth Des.* **11**, 5206-5214 (2011).

226. Ye, W. & Wang, X. Ribbon-like and rod-like hydroxyapatite crystals deposited on titanium surface with electrochemical method. *Mater. Lett.* **61**, 4062-4065 (2007).

227. Wei, Y. *et al.* Enamel repair with amorphous ceramics. *Adv. Mater.* **32**, 1907067 (2020).

228. Yamagishi, K. *et al.* A synthetic enamel for rapid tooth repair. *Nature* **433**, 819-819 (2005).

229. Aulestia, F. J. *et al.* Fluoride exposure alters Ca^2+^ signaling and mitochondrial function in enamel cells. *Sci. Signal.* **13**, eaay0086 (2020).

230. Liao, Y. *et al.* Identification and functional analysis of genome mutations in a fluoride-resistant *Streptococcus mutans* strain. *PLoS One* **10**, e0122630 (2015).

231. Chen, M. *et al.* Modulated regeneration of acid-etched human tooth enamel by a functionalized dendrimer that is an analog of amelogenin. *Acta Biomater.* **10**, 4437-4446 (2014).

232. Besinis, A., De Peralta, T., Tredwin, C. J. & Handy, R. D. Review of nanomaterials in dentistry: Interactions with the oral microenvironment, clinical applications, hazards, and benefits. *ACS Nano* **9**, 2255-2289 (2015).

233. Mukherjee, K. *et al.* Peptide-based bioinspired approach to regrowing multilayered aprismatic enamel. *ACS Omega* **3**, 2546-2557 (2018).

234. Shao, C. *et al.* Repair of tooth enamel by a biomimetic mineralization frontier ensuring epitaxial growth. *Sci. Adv.* **5**, eaaw9569 (2019).

235. Sowmya, S., Bumgardener, J. D., Chennazhi, K. P., Nair, S. V. & Jayakumar, R. Role of nanostructured biopolymers and bioceramics in enamel, dentin and periodontal tissue regeneration. *Prog. Polym. Sci.* **38**, 1748-1772 (2013).

236. Wang, D. *et al.* Controlling enamel remineralization by amyloid-like amelogenin mimics. *Adv. Mater.* **32**, 2002080 (2020).

237. Margolis, H. C., Beniash, E. & Fowler, C. E. Role of macromolecular assembly of enamel matrix proteins in enamel formation. *J. Dent. Res.* **85**, 775-793 (2006).

238. Bekshe Lokappa, S. *et al.* Interactions of amelogenin with phospholipids. *Biopolymers* **103**, 96-108 (2015).

239. Wang, Y. *et al.* in Bioinspired Materials Science and Engineering (eds Yang, G., Xiao, L., & Lamboni, L.) Ch. 18 (John Wiley & Sons, Inc., 2018).

240. Dunker, A. K., Silman, I., Uversky, V. N. & Sussman, J. L. Function and structure of inherently disordered proteins. *Curr. Opin. Struct. Biol.* **18**, 756-764 (2008).

241. Delak, K. *et al.* The tooth enamel protein, porcine amelogenin, is an intrinsically disordered protein with an extended molecular configuration in the monomeric form. *Biochemistry* **48**, 2272-2281 (2009).

242. Simmer, J. P. *et al.* Isolation and characterization of a mouse amelogenin expressed in *Escherichia coli*. *Calcif. Tissue Int.* **54**, 312-319 (1994).

243. Bromley, K. M. *et al.* Dissecting amelogenin protein nanospheres: Characterization of metastable oligomers. *J. Biol. Chem.* **286**, 34643-34653 (2011).

244. Wen, H., Fincham, A. G. & Moradian-Oldak, J. Progressive accretion of amelogenin molecules during nanospheres assembly revealed by atomic force microscopy. *Matrix Biol.* **20**, 387-395 (2001).

245. Martinez-Avila, O. *et al.* Self-assembly of filamentous amelogenin requires calcium and phosphate: From dimers *via* nanoribbons to fibrils. *Biomacromolecules* **13**, 3494-3502 (2012).

246. Moradian-Oldak, J. The emergence of "nanospheres" as basic structural components adopted by amelogenin. *J. Dent. Res.* **86**, 487-490 (2007).

247. Moradian-Oldak, J. & Goldberg, M. Amelogenin supra-molecular assembly *in vitro* compared with the architecture of the forming enamel matrix. *Cells Tissues Organs* **181**, 202-218 (2005).

248. Fincham, A. G. *et al.* Evidence for amelogenin "nanospheres" as functional components of secretory-stage enamel matrix. *J. Struct. Biol.* **115**, 50-59 (1995).

249. Moradian-Oldak, J., Tan, J. & Fincham, A. G. Interaction of amelogenin with hydroxyapatite crystals: An adherence effect through amelogenin molecular self-association. *Biopolymers* **46**, 225-238 (1998).

250. Kwak, S. *et al.* Regulation of calcium phosphate formation by amelogenins under physiological conditions. *Eur. J. Oral Sci.* **119**, 103-111 (2011).

251. Wang, L., Guan, X., Du, C., Moradian-Oldak, J. & Nancollas, G. H. Amelogenin promotes the formation of elongated apatite microstructures in a controlled crystallization system. *J. Phys. Chem. C* **111**, 6398-6404 (2007).

252. Uskoković, V., Li, W. & Habelitz, S. Amelogenin as a promoter of nucleation and crystal growth of apatite. *J. Cryst. Growth* **316**, 106-117 (2011).

253. Tarasevich, B. J. *et al.* The nucleation and growth of calcium phosphate by amelogenin. *J. Cryst. Growth* **304**, 407-415 (2007).

254. Hannig, M. & Hannig, C. Nanomaterials in preventive dentistry. *Nat. Nanotechnol.* **5**, 565-569 (2010).

255. Uskoković, V., Li, W. & Habelitz, S. Biomimetic precipitation of uniaxially grown calcium phosphate crystals from full-length human amelogenin sols. *J. Bionic Eng.* **8**, 114-121 (2011).

256. Fan, Y., Sun, Z., Wang, R., Abbott, C. & Moradian-Oldak, J. Enamel inspired nanocomposite fabrication through amelogenin supramolecular assembly. *Biomaterials* **28**, 3034-3042 (2007).

257. Habelitz, S. *et al.* Amelogenin-guided crystal growth on fluoroapatite glass-ceramics. *J. Dent. Res.* **83**, 698-702 (2004).

258. Wen, H., Moradian-Oldak, J. & Fincham, A. G. Modulation of apatite crystal growth on Bioglass® by recombinant amelogenin. *Biomaterials* **20**, 1717-1725 (1999).

259. Fan, Y. *et al.* Novel amelogenin-releasing hydrogel for remineralization of enamel artificial caries. *J. Bioact. Compat. Polym.* **27**, 585-603 (2012).

260. Ruan, Q. *et al.* Efficacy of amelogenin-chitosan hydrogel in biomimetic repair of human enamel in pH-cycling systems. *J. Biomed. Eng. Inform.* **2**, 119-128 (2016).

261. Ruan, Q. & Moradian-Oldak, J. Development of amelogenin-chitosan hydrogel for *in vitro* enamel regrowth with a dense interface. *J. Visualized Exp.*, e51606 (2014).

262. Ruan, Q., Siddiqah, N., Li, X., Nutt, S. & Moradian-Oldak, J. Amelogenin–chitosan matrix for human enamel regrowth: Effects of viscosity and supersaturation degree. *Connect. Tissue Res.* **55**, 150-154 (2014).

263. Ruan, Q., Zhang, Y., Yang, X., Nutt, S. & Moradian-Oldak, J. An amelogenin–chitosan matrix promotes assembly of an enamel-like layer with a dense interface. *Acta Biomater.* **9**, 7289-7297 (2013).

264. Uskoković, V. *et al.* Hydrolysis of amelogenin by matrix metalloprotease-20 accelerates mineralization *in vitro*. *Arch. Oral Biol.* **56**, 1548-1559 (2011).

265. Prajapati, S., Ruan, Q., Mukherjee, K., Nutt, S. & Moradian-Oldak, J. The presence of MMP-20 reinforces biomimetic enamel regrowth. *J. Dent. Res.* **97**, 84-90 (2017).

266. Dissanayake, S. S. M., Ekambaram, M., Li, K. C., Harris, P. W. R. & Brimble, M. A. Identification of key functional motifs of native amelogenin protein for dental enamel remineralisation. *Molecules* **25**, 4214 (2020).

267. Mukherjee, K., Ruan, Q., Liberman, D., White, S. & Moradian-Oldak, J. Repairing human tooth enamel with leucine-rich amelogenin peptide–chitosan hydrogel. *J. Mater. Res.* **31**, 1-8 (2016).

268. Le Norcy, E. *et al.* Leucine-rich amelogenin peptides regulate mineralization *in vitro*. *J. Dent. Res.* **90**, 1091-1097 (2011).

269. Shafiei, F. *et al.* Leucine-rich amelogenin peptide (LRAP) as a surface primer for biomimetic remineralization of superficial enamel defects: An *in vitro* study. *Scanning* **37**, 179-185 (2015).

270. Ravindranath, R. M. H., Devarajan, A. & Bringas, P. Enamel formation *in vitro* in mouse molar explants exposed to amelogenin polypeptides: ATMP and LRAP on enamel development. *Arch. Oral Biol.* **52**, 1161-1171 (2007).

271. Kwak, S. Y., Litman, A., Margolis, H. C., Yamakoshi, Y. & Simmer, J. P. Biomimetic enamel regeneration mediated by leucine-rich amelogenin peptide. *J. Dent. Res.* **96**, 524-530 (2017).

272. Hossein, B. G. *et al.* Study on the influence of leucine-rich amelogenin peptide (LRAP) on the remineralization of enamel defects *via* micro-focus X-ray computed tomography and nanoindentation. *Biomed. Mater.* **10**, 035007 (2015).

273. Dogan, S. *et al.* Biomimetic tooth repair: Amelogenin-derived peptide enables *in vitro* remineralization of human enamel. *ACS Biomater. Sci. Eng.* **4**, 1788-1796 (2018).

274. Lv, X. *et al.* Potential of an amelogenin based peptide in promoting reminerlization of initial enamel caries. *Arch. Oral Biol.* **60**, 1482-1487 (2015).

275. Ding, L. *et al.* Remineralization of enamel caries by an amelogenin-derived peptide and fluoride *in vitro*. *Regener. Biomater.* **7**, 283-292 (2020).

276. Ren, Q. *et al.* Anti-biofilm and remineralization effects of chitosan hydrogel containing amelogenin-derived peptide on initial caries lesions. *Regener. Biomater.* **5**, 69-76 (2018).

277. Li, D. *et al.* Remineralization of initial enamel caries *in vitro* using a novel peptide based on amelogenin. *Front. Mater. Sci.* **9**, 293-302 (2015).

278. Li, Z. *et al.* Comparing the efficacy of hydroxyapatite nucleation regulated by amino acids, poly-amino acids and an amelogenin-derived peptide. *CrystEngComm* **22**, 3814-3823 (2020).

279. Ren, Q. *et al.* Chitosan hydrogel containing amelogenin-derived peptide: Inhibition of cariogenic bacteria and promotion of remineralization of initial caries lesions. *Arch. Oral Biol.* **100**, 42-48 (2019).

280. Han, S. *et al.* Promotion of enamel caries remineralization by an amelogenin-derived peptide in a rat model. *Arch. Oral Biol.* **73**, 66-71 (2017).

281. Kolenbrander, P. E., Palmer, R. J., Periasamy, S. & Jakubovics, N. S. Oral multispecies biofilm development and the key role of cell–cell distance. *Nat. Rev. Microbiol.* **8**, 471-480 (2010).

282. Hannig, M. & Joiner, A. in The Teeth and Their Environment (ed Duckworth, R. M.) Ch. 2 (Karger, 2006).

283. Hay, D. I. The interaction of human parotid salivary proteins with hydroxyapatite. *Arch. Oral Biol.* **18**, 1517-1529 (1973).

284. Hay, D. I., Smith, D. J., Schluckebier, S. K. & Moreno, E. C. Basic biological sciences relationship between concentration of human salivary statherin and inhibition of calcium phosphate precipitation in stimulated human parotid saliva. *J. Dent. Res.* **63**, 857-863 (1984).

285. Johnsson, M., Richardson, C. F., Bergey, E. J., Levine, M. J. & Nancollas, G. H. The effects of human salivary cystatins and statherin on hydroxyapatite crystallization. *Arch. Oral Biol.* **36**, 631-636 (1991).

286. Raj, P. A., Johnsson, M., Levine, M. J. & Nancollas, G. H. Salivary statherin. Dependence on sequence, charge, hydrogen bonding potency, and helical conformation for adsorption to hydroxyapatite and inhibition of mineralization. *J. Biol. Chem.* **267**, 5968-5976 (1992).

287. Li, Y. *et al.* Hybrid nanotopographical surfaces obtained by biomimetic mineralization of statherin-inspired elastin-like recombinamers. *Adv. Healthc. Mater.* **3**, 1638-1647 (2014).

288. Shuturminska, K. *et al.* Elastin-like protein, with statherin derived peptide, controls fluorapatite formation and morphology. *Front. Physiol.* **8**, 368 (2017).

289. Yang, Y. *et al.* Salivary acquired pellicle-inspired DpSpSEEKC peptide for the restoration of demineralized tooth enamel. *Biomed. Mater.* **12**, 025007 (2017).

290. Yang, X. *et al.* A universal and ultrastable mineralization coating bioinspired from biofilms. *Adv. Funct. Mater.* **28**, 1802730 (2018).

291. Yang, X. *et al.* Bioinspired from mussel and salivary acquired pellicle: A universal dual-functional polypeptide coating for implant materials. *Mater. Today Chem.* **14**, 100205 (2019).

292. Gou, Y. *et al.* Bio-inspired peptide decorated dendrimers for a robust antibacterial coating on hydroxyapatite. *Polym. Chem.* **8**, 4264-4279 (2017).

293. Liu, Y. *et al.* Bioinspired heptapeptides as functionalized mineralization inducers with enhanced hydroxyapatite affinity. *J. Mater. Chem. B* **6**, 1984-1994 (2018).

294. Yang, X. *et al.* Bioinspired from salivary acquired pellicle: A multifunctional coating for biominerals. *Chem. Mater.* **29**, 5663-5670 (2017).

295. Yang, X. *et al.* Antibacterial and anti-biofouling coating on hydroxyapatite surface based on peptide-modified tannic acid. *Colloids Surf., B* **160**, 136-143 (2017).

296. Yang, X. *et al.* Bioinspired peptide-decorated tannic acid for *in situ* remineralization of tooth enamel: *in vitro* and *in vivo* evaluation. *ACS Biomater. Sci. Eng.* **3**, 3553-3562 (2017).

297. Zhang, S. *et al.* Effective *in situ* repair and bacteriostatic material of tooth enamel based on salivary acquired pellicle inspired oligomeric procyanidins. *Polym. Chem.* **7**, 6761-6769 (2016).

298. Segman-Magidovich, S., Grisaru, H., Gitli, T., Levi-Kalisman, Y. & Rapaport, H. Matrices of acidic *β*-sheet peptides as templates for calcium phosphate mineralization. *Adv. Mater.* **20**, 2156-2161 (2008).

299. Firth, A., Aggeli, A., Burke, J. L., Yang, X. & Kirkham, J. Biomimetic self-assembling peptides as injectable scaffolds for hard tissue engineering. *Nanomedicine* **1**, 189-199 (2006).

300. Aggeli, A. *et al.* pH as a trigger of peptide *β*-sheet self-assembly and reversible switching between nematic and isotropic phases. *J. Am. Chem. Soc.* **125**, 9619-9628 (2003).

301. Kind, L. *et al.* Biomimetic remineralization of carious lesions by self-assembling peptide. *J. Dent. Res.* **96**, 790-797 (2017).

302. Kirkham, J. *et al.* Self-assembling peptide scaffolds promote enamel remineralization. *J. Dent. Res.* **86**, 426-430 (2007).

303. Philip, N. State of the art enamel remineralization systems: The next frontier in caries management. *Caries Res.* **53**, 284-295 (2019).

304. Brunton, P. A. *et al.* Treatment of early caries lesions using biomimetic self-assembling peptides—a clinical safety trial. *Br. Dent. J.* **215**, E6 (2013).

305. Cui, H., Webber, M. J. & Stupp, S. I. Self-assembly of peptide amphiphiles: From molecules to nanostructures to biomaterials. *Biopolymers* **94**, 1-18 (2010).

306. Hartgerink, J. D., Beniash, E. & Stupp, S. I. Self-assembly and mineralization of peptide-amphiphile nanofibers. *Science* **294**, 1684 (2001).

307. Li, Q. *et al.* A novel self-assembled oligopeptide amphiphile for biomimetic mineralization of enamel. *BMC Biotechnol.* **14**, 32 (2014).

308. George, A. *et al.* The carboxyl-terminal domain of phosphophoryn contains unique extended triplet amino acid repeat sequences forming ordered carboxyl-phosphate interaction ridges that may be essential in the biomineralization process. *J. Biol. Chem.* **271**, 32869-32873 (1996).

309. Chang, S. *et al.* Synthesis of a potentially bioactive, hydroxyapatite-nucleating molecule. *Calcif. Tissue Int.* **78**, 55-61 (2006).

310. Yang, Y. *et al.* Synergistic inhibition of enamel demineralization by peptide 8DSS and fluoride. *Caries Res.* **50**, 32-39 (2016).

311. Hsu, C., Chung, H., Yang, J., Shi, W. & Wu, B. Influence of 8DSS peptide on nano-mechanical behavior of human enamel. *J. Dent. Res.* **90**, 88-92 (2010).

312. Yang, Y. *et al.* 8DSS-promoted remineralization of initial enamel caries *in vitro*. *J. Dent. Res.* **93**, 520-524 (2014).

313. Zheng, W. *et al.* The effects of 8DSS peptide on remineralization in a rat model of enamel caries evaluated by two nondestructive techniques. *J. Appl. Biomater. Funct. Mater.* **17**, 2280800019827798 (2019).

314. Hsu, C., Chung, H., Yang, J., Shi, W. & Wu, B. Influences of ionic concentration on nanomechanical behaviors for remineralized enamel. *J. Mech. Behav. Biomed. Mater.* **4**, 1982-1989 (2011).

315. Chung, H., Li, C. & Hsu, C. Characterization of the effects of 3DSS peptide on remineralized enamel in artificial saliva. *J. Mech. Behav. Biomed. Mater.* **6**, 74-79 (2012).

316. Chung, H. & Li, C. Microstructure and nanomechanical properties of enamel remineralized with asparagine–serine–serine peptide. *Mater. Sci. Eng., C* **33**, 969-973 (2013).

317. Chung, H. & Huang, K. Effects of peptide concentration on remineralization of eroded enamel. *J. Mech. Behav. Biomed. Mater.* **28**, 213-221 (2013).

318. Li, C., Xu, L., Zuo, Y. Y. & Yang, P. Tuning protein assembly pathways through superfast amyloid-like aggregation. *Biomater. Sci.* **6**, 836-841 (2018).

319. Wang, D. *et al.* 2D protein supramolecular nanofilm with exceptionally large area and emergent functions. *Adv. Mater.* **28**, 7414-7423 (2016).

320. Liu, R. *et al.* One-step assembly of a biomimetic biopolymer coating for particle surface engineering. *Adv. Mater.* **30**, 1802851 (2018).

321. Carneiro, K. M. M. *et al.* Amyloid-like ribbons of amelogenins in enamel mineralization. *Sci. Rep.* **6**, 23105 (2016).

322. Zhang, J., Wang, J., Ma, C. & Lu, J. Hydroxyapatite formation coexists with amyloid-like self-assembly of human amelogenin. *Int. J. Mol. Sci.* **21**, 2946 (2020).

323. Deutsch, D. *et al.* Tuftelin—aspects of protein and gene structure. *Eur. J. Oral Sci.* **106**, 315-323 (1998).

324. Deutsch, D. *et al.* The human tuftelin gene and the expression of tuftelin in mineralizing and nonmineralizing tissues. *Connect. Tissue Res.* **43**, 425-434 (2002).

325. Ding, L. *et al.* Tuftelin-derived peptide facilitates remineralization of initial enamel caries *in vitro*. *J. Biomed. Mater. Res. B* **108**, 3261-3269 (2020).

326. Yan, S. *et al.* Effect of anionic PAMAM with amido groups starburst dendrimers on the crystallization of Ca_10_(PO_4_)_6_(OH)_2_ by hydrothermal method. *Mater. Chem. Phys.* **99**, 164-169 (2006).

327. Xie, L. *et al.* Effects of glutamic acid shelled PAMAM dendrimers on the crystallization of calcium phosphate in diffusion systems. *Polym. Bull.* **66**, 119-132 (2011).

328. Zhang, F. *et al.* Hydrothermal synthesis of hydroxyapatite nanorods in the presence of anionic starburst dendrimer. *Mater. Lett.* **59**, 1422-1425 (2005).

329. Zhou, Z., Zhou, P., Yang, S., Yu, X. & Yang, L. Controllable synthesis of hydroxyapatite nanocrystals *via* a dendrimer-assisted hydrothermal process. *Mater. Res. Bull.* **42**, 1611-1618 (2007).

330. Chen, H., Banaszak Holl, M., Orr, B. G., Majoros, I. & Clarkson, B. H. Interaction of dendrimers (artificial proteins) with biological hydroxyapatite crystals. *J. Dent. Res.* **82**, 443-448 (2003).

331. Xin, J. *et al.* Phosphorylated dendronized poly(amido amine)s as protein analogues for directing hydroxylapatite biomineralization. *Chem. Commun.* **50**, 6491-6493 (2014).

332. Chen, L. *et al.* Regeneration of biomimetic hydroxyapatite on etched human enamel by anionic PAMAM template *in vitro*. *Arch. Oral Biol.* **58**, 975-980 (2013).

333. Chen, L., Yuan, H., Tang, B., Liang, K. & Li, J. Biomimetic remineralization of human enamel in the presence of polyamidoamine dendrimers *in vitro*. *Caries Res.* **49**, 282-290 (2015).

334. Fan, M. *et al.* Remineralization effectiveness of the PAMAM dendrimer with different terminal groups on artificial initial enamel caries *in vitro*. *Dent. Mater.* **36**, 210-220 (2020).

335. Gao, Y. *et al.* Enamel remineralization *via* poly(amido amine) and adhesive resin containing calcium phosphate nanoparticles. *J. Dent.* **92**, 103262 (2020).

336. Zhang, X. *et al.* Biomimetic remineralization of demineralized enamel with nano-complexes of phosphorylated chitosan and amorphous calcium phosphate. *J. Mater. Sci.: Mater. Med.* **25**, 2619-2628 (2014).

337. Xiao, Z. *et al.* Rapid biomimetic remineralization of the demineralized enamel surface using nano-particles of amorphous calcium phosphate guided by chimaeric peptides. *Dent. Mater.* **33**, 1217-1228 (2017).

338. Wang, H. *et al.* Oriented and ordered biomimetic remineralization of the surface of demineralized dental enamel using HAP@ACP nanoparticles guided by glycine. *Sci. Rep.* **7**, 40701 (2017).

339. Onuma, K., Yamagishi, K. & Oyane, A. Nucleation and growth of hydroxyapatite nanocrystals for nondestructive repair of early caries lesions. *J. Cryst. Growth* **282**, 199-207 (2005).

340. Li, L. *et al.* Repair of enamel by using hydroxyapatite nanoparticles as the building blocks. *J. Mater. Chem.* **18**, 4079-4084 (2008).

341. Li, L. *et al.* Bio-inspired enamel repair *via* Glu-directed assembly of apatite nanoparticles: An approach to biomaterials with optimal characteristics. *Adv. Mater.* **23**, 4695-4701 (2011).

342. Nassif, N. *et al.* Amorphous layer around aragonite platelets in nacre. *Proc. Natl. Acad. Sci. U. S. A.* **102**, 12653 (2005).

343. DeVol, R. T. *et al.* Nanoscale transforming mineral phases in fresh nacre. *J. Am. Chem. Soc.* **137**, 13325-13333 (2015).

344. Mass, T. *et al.* Amorphous calcium carbonate particles form coral skeletons. *Proc. Natl. Acad. Sci. U. S. A.* **114**, E7670 (2017).

345. Liu, Z. *et al.* Crosslinking ionic oligomers as conformable precursors to calcium carbonate. *Nature* **574**, 394-398 (2019).

346. DeRocher, K. A. *et al.* Chemical gradients in human enamel crystallites. *Nature* **583**, 66-71 (2020).

347. Qiu, Z. *et al.* Mineralized collagen: Rationale, current status, and clinical applications. *Materials* **8**, 4733-4750 (2015).

348. Ye, Z. *et al.* Biomimetic mineralized hybrid scaffolds with antimicrobial peptides. *Bioact. Mater.* **6**, 2250-2260 (2021).

349. Liu, H. *et al.* Doping bioactive elements into a collagen scaffold based on synchronous self-assembly/mineralization for bone tissue engineering. *Bioact. Mater.* **5**, 844-858 (2020).

350. Wan, Q. *et al.* Simultaneous regeneration of bone and nerves through materials and architectural design: Are we there yet? *Adv. Funct. Mater.* **30**, 2003542 (2020).

351. Yin, S., Zhang, W., Zhang, Z. & Jiang, X. Recent advances in scaffold design and material for vascularized tissue-engineered bone regeneration. *Adv. Healthc. Mater.* **8**, 1801433 (2019).

Fig. 1

Schematic of biomineralization-inspired materials for hard tissue repair. The formation processes and mechanisms of human hard tissues, including bones and teeth, have inspired the design and construction of various materials for hard tissue repair. These materials mainly include recombinant proteins, synthetic peptides, dendrimers, polyelectrolytes-stabilized mineral precursors, mineralized scaffolds, and inorganic materials. The applications of these materials are categorized into three parts to be introduced in this review: bone repair, dentin remineralization, and enamel remineralization.

**Fig. 2** Schematics, characterizations, and *in vivo* effects of intrafibrillarly mineralized collagen scaffolds. Structural schematics of (a1) native bone, (b1) HIMC, (c1) NIMC, and (d1) EMC, respectively. TEM images of (a2) native bone, (b2) HIMC, (c2) NIMC, and (d2) EMC, respectively. The HIMC shows bone-like periodical nanoarchitectures, whereas the NIMC shows no periodicity. In the EMC, HAP clusters deposit randomly outside collagen fibrils. (e) Micro-CT images and bone volume of different scaffolds at 12 weeks post-transplantation in rat mandibles. The HIMC shows similar bone repairing effects with the DCBM. (f) H&E and Masson stainings, and semiquantitative analysis of regenerated bones in rat mandibles. The HIMC and the DCBM groups show abundant neo-bone formation and regeneration of osteoblasts and bone marrow, which are more than those of the NIMC group. Reproduced with permission from ref. [78](#_ENREF_78) 2019 Wiley-VCH.

**Fig. 3** *In vivo* experiments of CaP-PILP on osteoporotic mouse. (a) Photographs of percutaneously injecting 30 μL of CaP-PILP into the osteoporotic tibia of mouse showing its mini-invasively injectability. (b-m) 2D, 3D micro-CT, and H&E staining of osteoporotic bone after the injection of CaP-PILP at (b, f, j) 0 weeks, (c, g, k) 4 weeks, (d, h, l) 8 weeks, and (e, i, m) 12 weeks. The CaP-PILP-recovered bone shows a significant new bone generation over time and it reaches the summit at 8 weeks. Scale bars: (b–e) 100 μm; (f–i) 300 μm; and (j–m) 200 μm. Reproduced from ref. 123 2019 Yao *et al*.

Fig. 4

Schematics and characterizations of biomineralization-inspired materials for dentin remineralization. (I) (a) TEM image of P26-collagen self-assembly. The assembled P26 peptides are dispersed nanospheres around the collagen fibril. (b) TEM image of collagen mineralization with P26 shows intrafibrillar mineralization and the inserted selected-area electron diffraction (SAED) image indicates the presence of HAP. Cross-sectional scanning electron microscopy (SEM) images of dentin after remineralization: (c, d) Control, and (e, f) with P26. By comparison, P26-treated dentin shows more apparent remineralization inside the dentinal tubules, which is reflected by a distinct string-of-beads morphology of the collagen fibrils. (II) (a-d) TEM images of ACP on collagen fibrils pretreated with different concentrations of citrate; (a) 0. (b) 25 × 10^–3^ _M_. (c) 50 × 10^–3^ _M_. (d) 100 × 10^–3^ _M_. These images indicate that citrate facilitates the infiltration of ACP into collagen fibrils. (e) Schematic of collagen mineralization *via* citrate pretreatment. Citrate decreases the contact angle and improves the wetting of ACP on collagen fibrils, and further promotes the degree of intrafibrillar mineralization. TEM images of remineralized dentin (f) without treatment and (g) with 100 × 10^–3^ _M_ citrate treatment. By contrast, citrate significantly promotes dentin remineralization. Scale bars: 50 nm (a-d), and 1 μm (f) and (g). (I) was reproduced with permission from ref. 145 2020 American Chemical Society. (II) was reproduced with permission from ref. 134 2018 Wiley-VCH

Fig. 5

Schematics and characterizations of amelogenin for enamel remineralization. (I) (a) Schematic of the formation of oligomer and nanosphere at different pH values. With the increase of pH, the amelogenin residues with positive charges are gradually deprotonated, thus the weak hydrophobic interactions lead to the formation of the nanospheres. (b) TEM images of the linear arrays of amelogenin nanospheres. (c) SEM image of a mature amelogenin ribbon showing well-defined edges. (II) (a) SEM images of native enamel, in which the arrows show the enamel orientation. (b-d) SEM images of newly formed crystal layer after the remineralization treatment with CS-AMEL hydrogel for 1 week. (b) Red rectangles 1 and 2 in the inserted image are selected regions in (b) and (c). White arrows exhibit the orientation of newly formed crystal layer. (c) The new layer is closely combined to the enamel surface. (d) Red arrows show the typical bundle of parallelly aligned crystals inside the new layer. The inserted image shows the homogenous surface of the new layer. (I) was reproduced with permissions from ref. 244 2011 American Society for Biochemistry and Molecular Biology, and ref. 214 2005 American Association for the Advancement of Science. (II) was reproduced with permission from ref. 264 2013 Elsevier.

**Fig. 6** Schematic and *in vivo* experiments of PTL/C-AMG for enamel remineralization. (a) Schematic showing the similarity between amelogenin and PTL/C-AMG matrix in regulating the transition of ACP into HAP on enamel. (b) Schematic and photographs showing the process of fixing demineralized enamel slices in rat’s oral cavity. SEM images of (c) the untreated demineralized enamel, (d) demineralized enamel treated with fluoride, and (e) PTL/C-AMG film-treated demineralized enamel, after 2 weeks of remineralization in oral cavity. (c2), (d2), and (e2) are the corresponding high-magnification images of (c1), (d1), and (e1), respectively. The untreated group shows only incompact and irregular minerals. The fluoride group exhibits hollow cracks and irregular crystals. The PTL/C-AMG film group shows a “fish-scale” morphology that is similar to native enamel, and the newly formed crystals are highly oriented. Reproduced with permission from ref. 237 2020 Wiley-VCH

Fig. 7

Schematics and characterizations of CPICs for enamel remineralization. (a) Schematic of ACP formation: ACP is formed when the stabilizer (TEA) is removed from CPICs. (b) Schematic of the epitaxial growth of crystalline HAP. The coating CPICs solution transforms into an amorphous frontier on the HAP surface, and then transforms into HAP. (c) SEM image exhibiting CPICs-repaired enamel and acid-etched enamel. The repaired enamel shows a similar morphology to that of native enamel. (d) 3D atomic force microscopy (AFM) image of repaired enamel indicates the formation of a new HAP layer. (e) High-magnification SEM image of the red circle in (c), which shows the similar morphological texture between the repaired and native enamel. Scale bars: 20 μm (c), and 2 μm (e). Reproduced with permission from ref. 235 2019 American Association for the Advancement of Science.
