# Supplementary material for: Advances in biomineralization-inspired materials for hard tissue repair
Source: Int J Oral Sci. 2021 Dec 7;13:42. doi: 10.1038/s41368-021-00147-z (PMC8651686; doi:10.1038/s41368-021-00147-z)
Supplement: Supplementary file 5 — Permission of Figure 3 [file 41368_2021_147_MOESM5_ESM.pdf]

Copyright Clearance Center RightsLink®

Help Email Support

Publisher: John Wiley and Sons

© 2019 The Authors. Published by WILEY-VCH Verlag GmbH & Co. KGaA, Weinheim

Open Access Article

This is an open access article distributed under the terms of the Creative Commons CC BY license, which permits unrestricted use, distribution, and reproduction in any medium, provided the original work is properly cited.

You are not required to obtain permission to reuse this article.

For an understanding of what is meant by the terms of the Creative Commons License, please refer to Wiley's Open Access Terms and Conditions.

Permission is not required for this type of reuse.

Wiley offers a professional reprint service for high quality reproduction of articles from over 1400 scientific and medical journals. Wiley's reprint service offers:

• Peer reviewed research or reviews
• Tailored collections of articles
• A professional high quality finish
• Glossy journal style color covers
• Company or brand customisation
• Language translations
• Prompt turnaround times and delivery directly to your office, warehouse or congress.

Please contact our Reprints department for a quotation. Email corporatesaleseurope@wiley.com or corporatesalesusa@wiley.com or corporatesalesDE@wiley.com.

© 2021 Copyright - All Rights Reserved | Copyright Clearance Center, Inc. | Privacy statement | Terms and Conditions
Comments? We would like to hear from you. E-mail us at customercare@copyright.com
