# Supplementary material for: Advances in biomineralization-inspired materials for hard tissue repair
Source: Int J Oral Sci. 2021 Dec 7;13:42. doi: 10.1038/s41368-021-00147-z (PMC8651686; doi:10.1038/s41368-021-00147-z)
Supplement: Supplementary file 12 — Permission of Figure 7 [file 41368_2021_147_MOESM12_ESM.pdf]

Gmail **Shuxian Tang <tangshuxian1998@gmail.com>**

TERMS & CONDITIONS - SCI ADVANCES FIGURES - SHAO ET AL 2019 - JOURNAL USE

1 message

permissions <permissions@aaas.org> Sun, Jan 24, 2021 at 10:52 PM
To: Shuxian Tang <tangshuxian1998@gmail.com>

Re: Use of AAAS *Science Advances* journal figures in a new journal article you are writing

Dear Shuxian, Tang:

Thank you very much for your request and for your interest in the *Science Advances* material identified in your email. My apologies for the delay, we've had a high volume of requests and it is taking me more time to respond. If you can still include the material, we are pleased to grant permission subject to the terms & conditions listed below. For clarity, this permission covers use of the *Science Advances* material in a commercial journal.

Please let me know if you have any questions or if anything more is needed.

Kind regards,

Liz

(Ms.) Elizabeth Sandler

Rights & Permissions

Science family of journals/AAAS

1200 New York Ave. NW

Washington, DC 20005

permissions@aaas.org

****

Terms and Conditions for using AAAS *Science Advances* journal figures in a new journal article you are writing.

AAAS hereby grants you the non-exclusive worldwide right to publish the figure(s) you've identified in your email below in a new journal article you are writing, subject to the following conditions:

1. Permission does not apply to figures/photos/artwork or any other content or materials included in the *Science Advances* article that are credited to non-AAAS sources. If the requested material is sourced to or references non-AAAS sources, you must obtain authorization from that source and this permission will be null & void.

2. If you are using figure(s)/table(s), permission is granted for use in print and electronic versions of your journal article.

3. The following credit line must be printed along with the AAAS material: "Reprinted/adapted from [INSERT Science Advances REFERENCE CITATION]. © The Authors, some rights reserved; exclusive licensee AAAS. Distributed under a Creative Commons Attribution NonCommercial License 4.0 (CC BY-NC) http://creativecommons.org/licenses/by-nc/4.0/”

4. If any modifications are made to the material, those changes must be identified.

5. Use of the material must not imply any endorsement by the authors or by AAAS and *Science Advances*.

6. No legal terms or technological measures may be applied to the reproduced material that conflicts with CC BY-NC license terms.

7. AAAS must publish the full *Science Advances* article prior to your use of any of its text or figures.

8. By using the AAAS Material identified in your request, you agree to abide by all the terms and conditions herein.

By using the AAAS material identified you agree to hold harmless and indemnify AAAS against any claims arising from your use of any content in your work that is credited to non-AAAS sources.

AAAS makes no representations or warranties as to the accuracy of any information contained in the AAAS material covered by this permission, including any warranties of merchantability or fitness for a particular purpose.

If you have any questions please contact the Permissions Department at permissions@aaas.org. Be sure to include this email thread in your correspondence.

****

Elizabeth Sandler (Ms.)

Rights & Permissions, *Science* family of journals

American Association for the Advancement of Science (AAAS)

1200 New York Avenue

Washington, DC 20005

E: esandler@aaas.org

Tel: +1-202-326-6765

From: Shuxian Tang <tangshuxian1998@gmail.com>
Sent: Saturday, January 2, 2021 4:51 AM
To: permissions <permissions@aaas.org>
Subject: Permission Request

[EXTERNAL EMAIL]

Dear editors,

I am preparing a journal review titled "Advances of biomineralization-inspired materials for hard tissue repair" in **Bone Research**, Springer Nature. In this manuscript, I cited a paper published by the Science Advances. I hope you can grant me the right to use Fig. 1, 2 and 3 in this paper, which will be referenced in my manuscript and will not be used for any commercial purpose.

The required information about the content I wish to use is listed here:

1. AAAS publication title: Science Advances.

2. Article title: Repair of tooth enamel by a biomimetic mineralization frontier ensuring epitaxial growth.

3. Authors' names:Shao, Changyu; Jin, Biao; Mu, Zhao; Lu, Hao; Zhao, Yueqi; Wu, Zhifang; Yan, Lumiao; Zhang, Zhisen; Zhou, Yanchun; Pan, Haihua; Liu, Zhaoming; Tang, Ruikang.

4. Volume number: 5, issue date: 8, page numbers: eaaw9569.

5. Specific figure numbers: Fig. 1, 2 and 3.

The required information about the intended use of the content is listed here:

1. Type of work in which your material will be used: Journal.

2. Title of work in which AAAS material will appear: Biomineralization-inspired materials for enamel remineralization.

3. Title of article: Advances of biomineralization-inspired materials for hard tissue repair.

4. Authors: Shuxian Tang, Zhiyun Dong, Jun Luo, and Jianshu Li.

5. Expected publication date: June, 1, 2021.

6. Publishing company: Springer Nature.

7. The formats my work will be distributed in: Print and electronic.

8. The rights I'm seeking: To reproduce the three required figures in my new work.

My information is listed here:

1. Name: Shuxian, Tang.

2. Institute: Sichuan University.

3. Title: Graduate Student.

4. Mailing address: Sichuan University, No.24 South Section 1, Yihuan Road, Chengdu , China, 610065.

5. Phone number: +86, 18810526056.

Thank you very much!

Sincerely,

Shuxian, Tang
